# Possibilities of using the German Federal States’ permanent soil monitoring program for the monitoring of potential effects of genetically modified organisms (GMO)

**DOI:** 10.1186/s12302-015-0057-2

**Published:** 2015-10-23

**Authors:** Andreas Toschki, Stephan Jänsch, Martina Roß-Nickoll, Jörg Römbke, Wiebke Züghart

**Affiliations:** 1 gaiac Research Institute for Ecosystem Analysis and Assessment, Kackerstr. 10, 52072 Aachen, Germany; 2 ECT Oekotoxikologie GmbH, Böttgerstr. 2-14, 65439 Flörsheim, Germany; 3 RWTH Aachen University, Institute for Environmental Research, Worringer Weg 1, 52074 Aachen, Germany; 4 Federal Agency for Nature Conservation (BfN), Konstantinstr. 110, 53179 Bonn, Germany

**Keywords:** GMO, Permanent soil monitoring, PSM sites, Soil organisms

## Abstract

**Background:**

In the Directive 2001/18/EC on the deliberate release of genetically modified organisms (GMO) into the environment, a monitoring of potential risks is prescribed after their deliberate release or placing on the market. Experience and data of already existing monitoring networks should be included. The present paper summarizes the major findings of a project funded by the Federal Agency for Nature Conservation (Nutzungsmöglichkeiten der Boden—Dauerbeobachtung der Länder für das Monitoring der Umweltwirkungen gentechnisch veränderter Pflanzen. BfN Skripten, Bonn-Bad Godesberg 369, 2014). The full report in german language can be accessed on http://www.bfn.de and is available as Additional file 1. The aim of the project was to check if it is possible to use the German permanent soil monitoring program (PSM) for the monitoring of GMO. Soil organism communities are highly diverse and relevant with respect to the sustainability of soil functions. They are exposed to GMO material directly by feeding or indirectly through food chain interactions. Other impacts are possible due to their close association to soil particles.

**Results:**

The PSM program can be considered as representative with regard to different soil types and ecoregions in Germany, but not for all habitat types relevant for soil organisms. Nevertheless, it is suitable as a basic grid for monitoring the potential effects of GMO on soil invertebrates.

**Conclusions:**

PSM sites should be used to derive reference values, i.e. range of abundance and presence of different relevant species of soil organisms. Based on these references, it is possible to derive threshold values to define the limit of acceptable change or impact. Therefore, a minimum set of sites and minimum set of standardized methods are needed, i.e. characterization of each site, sampling of selected soil organism groups, adequate adaptation of methods for the purpose of monitoring of potential effects of GMO. Finally, and probably most demanding, it is needed to develop a harmonized evaluation concept.

**Electronic supplementary material:**

The online version of this article (doi:10.1186/s12302-015-0057-2) contains supplementary material, which is available to authorized users.

## Background

In the Directive 2001/18/EC on the deliberate release of genetically modified organisms (GMO) into the environment, a monitoring of potential adverse effects, including cumulative long-term effects is prescribed after placing on the market of GMO. The aim of this monitoring is to trace and identify any direct or indirect, immediate, delayed or unforeseen effects on human health or the environment to enable fast action if necessary. Therefore, the Directive divides the monitoring into two principal components (1) case-specific monitoring and (2) general surveillance [2]. The former is focused on a constant check whether the outcome of the risk assessment performed when notifying a GMO is correct. In the latter, the emphasis is to survey those effects in the environment that are unexpected. According to Directive 2001/18/EC, the notifier of the GMO in question is responsible for the execution of the monitoring. Where existing monitoring networks are suitable, experience and data should be included in the monitoring and interpretation process [3]. The natural function of soil as a habitat for soil organisms and thus soil biodiversity are one of the protection goals to be considered in this context. Against this background, the question needed to be answered whether the existing network of permanent soil monitoring (PSM) sites (in German: BDF = Bodendauerbeobachtungsflächen) is suitable for the purpose of monitoring of GMO. The overall aim of this contribution was to assess whether it is possible and sensible to use the PSM program of the German Federal States as part of the monitoring program required by Directive 2001/18/EC on the deliberate release into the environment of GMO [3, 4].

To this end, we discussed the following issues:

1. Relevance of PSM measurement parameters for GMO monitoring: Are the currently investigated site and soil parameters of the German PSM program relevant for the monitoring of potential effects of GMO cultivation?
2. Representativeness of PSM sites: Are the sites of the PSM program and their properties representative regarding the main ecological regions of Germany?
3. Exposure of PSM towards GMO: Have the sites of the PSM program already been exposed to GMO in the past?
4. Practicability: Which basic conditions concerning the monitoring sites have to be considered?

In conclusion, possible adaptions of the federal permanent soil monitoring and/or complementary monitoring modules for the GMO monitoring were formulated.

## Theoretical background

### The German permanent soil monitoring program

**Fig. 1:**
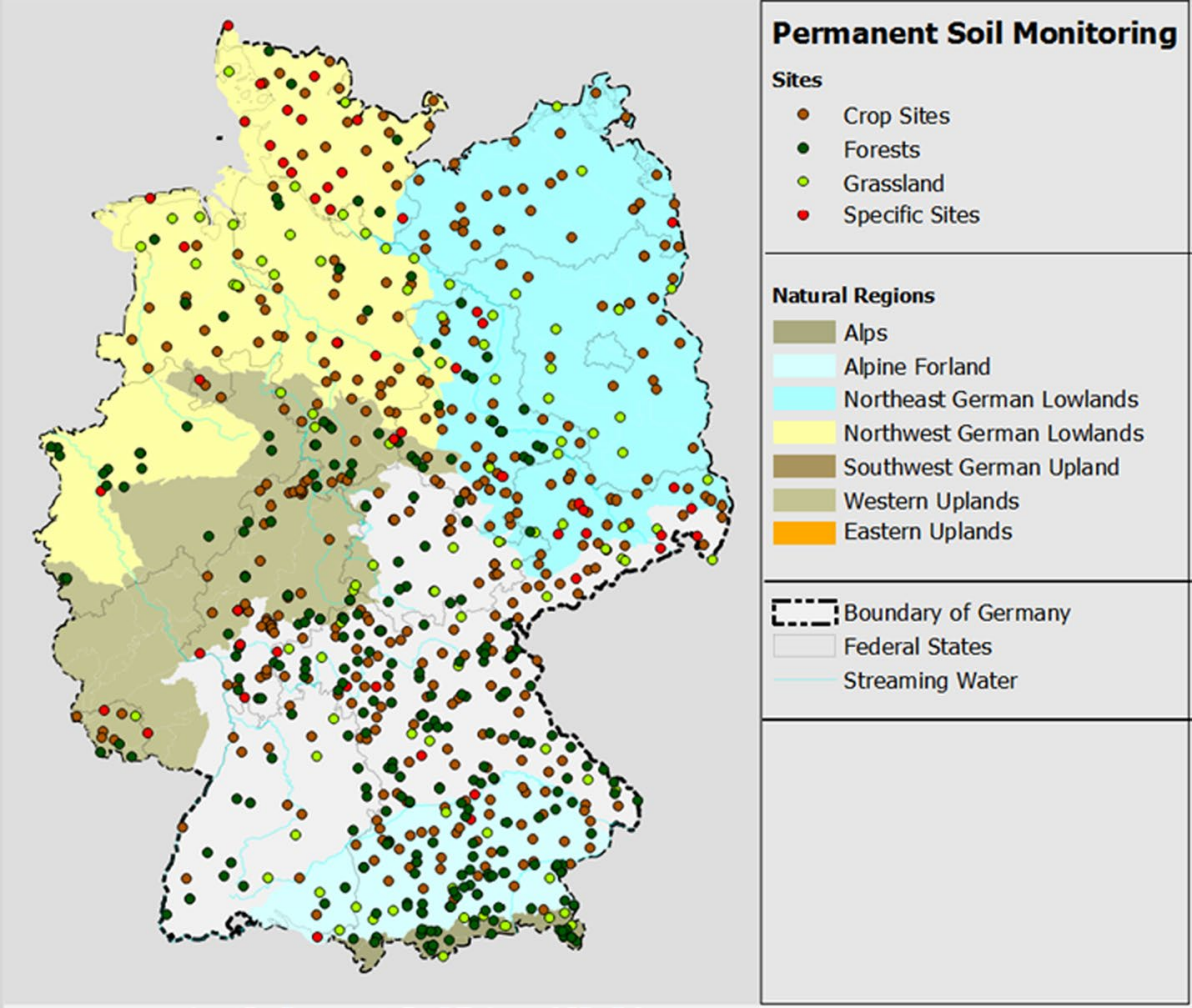
Overview of the permanent soil monitoring in Germany [6]

The aim of the German permanent soil monitoring program is, amongst others, to assess the current condition of soils and their monitoring in the long term to detect harmful soil changes and to project the function of soils [5]. To this end, there are 794 German PSM sites representing the regional soil forms, main land use and special pollution scenarios (Fig. 1). 344 of these are field sites, 146 grassland, 247 forests and the rest are special sites. Bremen, Berlin and Rhineland-Palatinate have no recorded PSM sites at the time. The largest federal state Bavaria harbours the highest share of all federal states (289 sites). Since 1990, the establishment and maintenance of PSM sites are standardized according to ISO guideline 16133 (ISO 2004). Sampling takes place on about 2500 m^2^ large plots that are divided into a core area (100 m^2^) and a surrounding border area for special investigations and containing a soil profile pit. There are mostly “basic” and a few “intensive” PSM sites. On the basic PSM sites, the long-term survey of changes of the soil condition is performed through comprehensive measurement of biological, chemical and physical soil parameters and additional information such as management practices. Depending on the parameter, the measurements are repeated in intervals ranging from 1 to 10 years. On the intensive PSM sites, there are additionally numerous continuous measurement devices, also recording atmospheric deposition and the chemical composition of liquid soil phase to investigate dynamic soil processes whose changes may occur in the short term. Measurement results are consolidated in an IT system (database “BDF—Bodendauerbeobachtungsflächen”) at the German Federal Environmental Agency within the scope of the administrative agreement of federal and state agencies regarding data exchange to enable cross-national evaluations.

### Structure and function of soil organism communities

Soil biota are thought to harbour a large part of the world’s biodiversity and to govern processes that are regarded as globally important components in the cycling of organic matter, energy and nutrients [7]. Rough estimates of the soil biodiversity indicate several thousands of invertebrate species apart from the largely unknown microbial and protozoan diversity. By far the most dominant groups of soil organisms, in terms of numbers and biomass, are the microbial organisms, i.e. bacteria and fungi [8]. Besides these organisms, soil ecosystems generally contain a large variety of animals, such as protozoa (bacterivores, omnivores, predators), nematodes (bacterivores, fungivores, omnivores, herbivores and predators), micro-arthropods such as mites (bacterivores, fungivores, predators) and collembolans (fungivores and predators), enchytraeids and earthworms (both mainly saprophagous). In addition, a high number of macrofauna species (mainly arthropods such as beetles, spiders, diplopods and chilopods or snails) are living in the uppermost soil layers, the soil surface and the litter layer. Anthropogenic activities clearly influence soil biota; most strongly in industrial or urban areas where only very few species can survive. Modern agricultural practices characterized by high levels of inorganic fertilizer additions, the use of pesticides and soil tillage are known to affect the diversity of the soil community, leading to the local loss or extinction of various groups of organisms [9].

With its large diversity and complexity, the soil community has a strong impact on soil processes, and the way in which these processes may vary in time and space. Most noteworthy are:

- decomposition of organic matter, thus regulating the cycling of nutrients;
- fixation of nitrogen from the air, making it available for plants;
- degradation of anthropogenic compounds such as pesticides;
- stabilization of soil aggregates, specifically by building clay-humus complexes;
- improval of soil porosity due to burrowing activities;
- influencing soil pH by nitrification and denitrification;
- being prey for many aboveground organisms.

As these processes also determine nutrient availability for take-up by plants, the belowground decomposer food web interactions also influence aboveground primary productivity and carbon sequestration [10]. In fact, plant productivity appears to increase by a reduced turnover of the microbial biomass due to stabilized carbon content and soil pH. The soil biomass is known to process over 100,000 kg of fresh organic material each year per hectare (25 cm top soil layer) in many agricultural systems. This processing includes the decomposition of dead organic matter by microbes as well as the consumption and production rates in the soil community food web [11–13]. The soil food web is defined as the structure and interactions across and between the communities of soil-living organisms and which are linked by conversions of energy and nutrients as one organism eats another. Therefore, most food web models merely provide a way to connect the dynamics of populations to the dynamics in ecological pathways within the cycling of matter, energy and nutrients [14].

### Exposure of soil organisms towards GMO

Information on the exposure of soil organism towards GMO can be found in literature [15–19]. Furthermore, the exposure of soil organisms has been intensively studied with organic chemicals [20–23]. These sources have been used to compile an overview on the exposure of soil organisms towards GMO.

### Exposure pathways of GMO for soil organisms

The definition and assessment of exposure pathways serve the purpose to estimate, whether or to what extent soil organisms (individual populations of a single species or whole communities) are confronted with any of the following impacts during GMO cultivation [20, 21]:

- GMO-specific active substances (e.g. *Bacillus thuringiensis* Cry proteins) or their metabolites;
- physiologically altered GM plant components (e.g. a modified starch content);
- agricultural management practices that would not be performed to the same extent without GMO cultivation (e.g. an increased application of herbicides).

Thus, the possible exposure pathways can be classified in various ways:

- organism-related approach [22]: direct and indirect exposure towards living or dead GMO materials or by uptake through the food chain; this classification is mainly based on experience with GM maize and the *Bt* toxin.
- GMO-related approach [24]: exposure through transport of GMO materials via pollen dispersal.

### Factors influencing the exposure of soil organisms to GMO

To assess the potential risks of GMO, the genetically modified plant as a whole and not only the isolated genetically modification must be taken into consideration. An affected organism can only react on that part of the GMO (toxin, dead plant material, pollen, etc.) which is bioavailable [20] or bioaccessible [23, 25]. The fraction of potentially harmful GMO material that is reaching the body tissues especially blood or lymph is relevant independently from the respective uptake pathway (e.g. water or food). The pathways of uptake depend on the individual species, while the bioavailability depends on site and soil characteristics. In addition, processes such as the biological degradation of the GMO material have to be taken into account, which are a function of time.

There are additionally also morphological, physiological and behavioural factors which strongly influence the exposure of soil organisms towards GMO [21]. Based on their biology, two groups of soil organisms can be identified which differ strongly in their way to take up chemicals: soft-bodied organisms, i.e. nematodes, enchytraeids, earthworms and hard-bodied organisms, i.e. mainly arthropods such as spiders, mites, collembolans, diplopods, isopods, or chilopods. Arthropods have special organs for water and oxygen uptake, while soft-bodied organisms use the body surface for these purposes. In addition, both groups can take up harmful substances by food. Different feeding types can be differentiated in soil organisms [26–28]: saprophages (feeding on dead organic material), microphages (feeding on bacteria), fungiphages (feeding on fungi), phytophages (feeding on living plants) and zoophages (predators) are the most common. However, evidence increases that many soil organisms are able to use different food resources [29–31]. Based on their mobility or preferences for different soil layers, most soil organism species can be classified into a limited number of ecological (trait) groups, e.g. epigeic, endogeic and anecic groups of earthworms [32].

### Principles of biodiversity monitoring

**Fig. 2:**
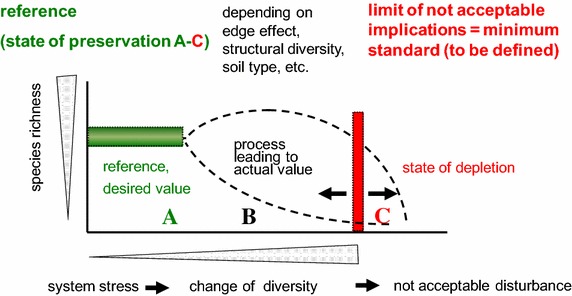
Derivation of threshold values regarding states of preservation (*A*–*C*; [6]) related to system stress (FFH regulation [33])

To facilitate the use of biocoenotical data at the landscape level, a standard frame of reference is needed. A reference system can be described as certain values for the biocoenosis at certain habitat types by evaluating biocoenosis-site-relationships and will ultimately lead to the identification of threshold values with which a significant change of the biocoenosis can be indicated (Fig. 2).

Thus, a reference system for the site-specific diversity of soil organisms consists of:

- reference values: lists of species expected to occur at a certain site with its specific conditions (e.g. climate, soil factors, region);
- a quantification of deviations from these reference values that indicate impacted habitat function.

To develop reference values that link soil and site parameters with the occurrence of soil organisms, the landscape had to be classified into a limited number of “site categories”. The use of the habitat classification concept compiled in the German Red Data Book on endangered habitats [34] ensures the compatibility with other monitoring approaches, nature conservation management, and prospectively also pesticide registration. When analysing different organism groups, correlations between the occurrence of species and the corresponding hierarchical level within the system of habitat types became apparent [6]. Further analysis demonstrated that the composition of communities depends on site properties. A comprehensive ecological assessment of sites requires the integration of different relevant organism groups on the species level thus at the same time covering their function (e.g. organic matter decomposition).

## Methods

### Relevance of PSM measurement parameters for GMO monitoring

Information on the structure and function of soil organism communities in Germany, especially at agricultural sites, was taken from the data base Bo-Info [6]; today part of the database Edaphobase [35, 36]. It contains both site-specific abiotic (e.g. soil properties) as well as biological as, for instance, species lists or data on the abundance of a certain organism group. 1744 sites (including 60 PSM sites) were covered, yielding about 42,473 datasets, 2000 of which are from PSM sites. The latter were contributed by five federal states: Brandenburg, Hamburg, North Rhine-Westphalia, Schleswig–Holstein and Thuringia (Fig. 1).

In parallel, information was compiled when developing a guideline for the monitoring of effects of GMO on soil organisms by the Association of German Engineers (VDI; [27]). This guideline and an explanatory paper on the same subject [28] represent suitable complements to the work described in this paper.

### Representativeness of PSM sites

To evaluate the question whether selected PSM sites can represent German landscapes, soil and site parameters and the occurrence of soil organisms have to be linked in a way that the whole landscape is classified into a limited number of “site categories”. For this, the habitat classification concept, compiled in the “German Red Data Book on endangered habitats”, was used [34, 37] (Table 1). It comprises 44 basic (first level) types with approximately 1000 hierarchically derived subtypes. This concept is already accepted by the German authorities and has been used in the areas of the European Habitats Directive [53], nature conservation management, GMO authorization and prospectively also pesticide registration. Habitat types harbour synecologically specific soil animal communities and additionally integrate the factors (in particular soil type, moisture and nutrient supply) relevant for a differentiation of communities [38, 39].

**Table 1:** Habitat types, derived from the German Red Data Book on endangered habitats [33, 36], used in this study for the establishment of a reference system to evaluate the biological state of the soil

Habitat type number	Description
*33.*	Arable and fallow land (in the following abbreviated ‘arable land’)
33.01	Farmed and fallow land on shallow skeletic calcareous soil
33.02	Farmed and fallow land on shallow skeletic silicaceous residual soil
33.03	Farmed and fallow land on sandy soil
33.04	Farmed and fallow land on loess, loam or clay soil
33.05	Farmed and fallow land on peaty or half-bog soil
*34.*	Natural dry grasslands and grasslands of dry to humid sites (in the following abbreviated ‘grassland’)
34.01	Xeric grassland
34.02	Semi-dry grassland
34.03	Steppic grassland (subcontinental, on deep soil)
34.04	Dry sandy grassland
34.05	Heavy-metal grassland
34.06	Mat-grass swards
34.07	Species-rich grassland on moist sites
34.08	Species-poor intensive grassland on moist sites
34.09	Trampled grass and park lawns
*43.*	Deciduous and mixed woodlands and forest plantations (deciduous share >50 %) (in the following abbreviated ‘deciduous forest’)
43.01	Birch bog woodland
43.02	Carr woodland
43.03	Swamp forest (on minerogenic soil)
43.04	Alluvial forest
43.05	Tidal alluvial forest
43.06	Ravine, boulder-field and scree forests
43.07	Deciduous and mixed forest on damp to moist sites
43.08	Deciduous (mixed) forest on dry or warm dry sites
43.09	Deciduous (mixed) plantations with native tree species
43.10	Deciduous (mixed) plantations with introduced tree species (including subspontaneous colonisations)
*44.*	Coniferous (mixed) woodlands and forest plantations (in the following abbreviated ‘coniferous forest’)
44.01	Bog woodland (coniferous)
44.02	Natural and near-natural dry to intermittently damp pine forest
44.03	Spruce/fir (mixed) forest and spruce (mixed) forest
44.04	Coniferous (mixed) plantations with native tree species
44.05	Coniferous (mixed) plantations with introduced tree species (including subspontaneous colonisations)

Italic types at first hierarchical level. Normal types at second hierarchical level

### Exposure of PSM towards GMO

PSM sites are potentially exposed towards GMO, possibly influencing their usability within a monitoring program. The following exposure scenarios are generally possible:

1. GMO cultivation on the PSM site;
2. the PSM site is located outside the area of GMO influence;
3. no GMO cultivation on the PSM site itself but PSM site lies within the area of direct influence of the GMO.

**Fig. 3:**
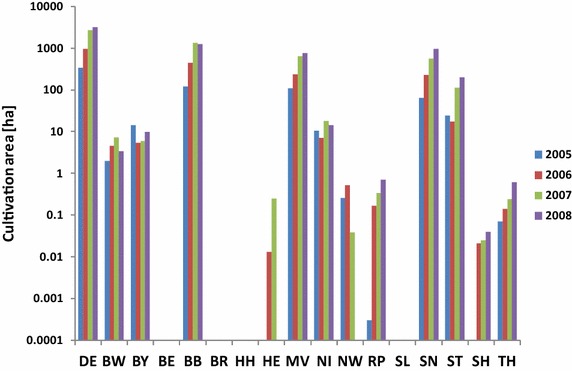
GM maize variety MON810 in Germany and the federal states for the years 2005–2008. Area under cultivation, *DE* Germany, *BW* Baden-Wuerttemberg, *BY* Bavaria, *BE* Berlin, *BB* Brandenburg, *BR* Bremen, *HH* Hamburg, *HE* Hesse, *MV* Mecklenburg-Western Pommerania, *NI* Lower Saxony, *NW* North Rhine-Westphalia, *RP* Rhineland-Palatinate, *SL* Saarland, *SN* Saxony, *ST* Saxony-Anhalt, *SH* Schleswig–Holstein, *TH* Thuringia. Data source: public location register of the German Federal Office of Consumer Protection and Food Safety (BVL)

Generally, it is permitted to cultivate GMO on PSM sites. However, until today GMO cultivation on PSM sites was not recorded. Hence, in the following, only the impact of GMO from “outside” influencing the PSM sites was assessed. Only pollen dispersal was considered as a pathway with a profound impact potential, while other pathways were regarded as being less relevant due to spatially and temporarily limited GMO release and the strong alteration (e.g. decomposition) of GMO material in dung, manure or sewage sludge. Literature data regarding the dispersal of pollen vary from a few metres to several kilometres [40, 41]. To ensure a sustainable use of the PSM program for the monitoring of GMO, the question had to be answered to what extent existing PSM sites had already been exposed towards GMO. The insect resistant maize variety MON810 was chosen as an example of GMO, the only GMO cultured on a broader scale in Germany so far until its cultivation was prohibited in 2009 (Fig. 3). Pollen dispersal radii of 50, 150 and 1000 m were assumed in analogy to different European buffer zone regulations towards conventional fields (e.g. Spain: 50 m, Germany: 150 m; [42]) and recommendations from field trials on maize pollen dispersal [43, 44].

These scenarios were exemplarily investigated for the federal states of Brandenburg, Hesse, Lower Saxony and Schleswig–Holstein. For this purpose, the Integrated Administration and Control System (IACS) data for these federal states that identify agriculturally managed parcels of land were combined with the coordinates of the PSM sites and of the MON810 field sites notified to the Public Location Register of the German Federal Office of Consumer Protection and Food Safety (BVL) in a geographic information system (GIS). The pollen dispersal radii were projected as buffer zones around the MON810 field sites. The resulting maps have a residual uncertainty regarding the exact position of the MON810 field sites but represent a sufficient approximation for this exemplary exercise.

### Practicability

Issues of practicability in the context of GMO monitoring can be divided into two areas: performance of biological soil monitoring in general and GMO-specific issues to be considered in such programs. The first issue will be discussed on the basis of own experiences when assessing biological soil quality at a high number of sites, both within the German PSM program but also European projects. The second issue was compiled after discussing how GMO crops are cultivated in general with farmers and representatives of the responsible agencies.

## Results and discussion

### Relevance of PSM measurement parameters for GMO monitoring

So far the German permanent soil monitoring program is focusing on environmental and soil parameters alone, despite the fact that several biological parameters (including soil organisms) were recommended as part of the standard sampling program [45]. Data on vegetation are only recorded in the federal state of Schleswig–Holstein. For seven federal states, data on soil microbial respiration are regularly available. In five federal states (Brandenburg, Hamburg, North Rhine-Westphalia Schleswig–Holstein and Thuringia), zoological data have been gathered (Table 2). However, even in those states only earthworms and enchytraeids have been sampled so far and usually only 2–3 times over the last 15 years. A uniform classification of all sites according to habitat type, vegetation units, etc. which is essential for ecological comparison purposes, is not applied at all.

**Table 2:** Number of datasets on zoology, vegetation and microbiology within the permanent soil monitoring program of the federal states of Germany

Federal state	No. of sites	Sum of data sets		
		Zoology	Microbiology	Vegetation
Baden-Württemberg	33	0	0	0
Bavaria	289	0	124	0
Berlin	0	0	0	0
Brandenburg	32	3241	178	0
Bremen	0	0	0	0
Hamburg	3	137	0	0
Hesse	68	0	0	0
Lower Saxony	46	0	0	0
Western Pomerania	90	0	635	0
North Rhine-Westfalia	21	743	60	0
Rhine land-Palatinate	0	0	0	0
Saarland	11	0	0	0
Saxony	55	0	0	0
Saxony-Anhalt	78	0	727	0
Schleswig-Holstein	37	1599	1576	2478
Thuringia	32	77	120	0
Sum	795	5797	3420	2478

The data sets originating from PSM sites contain little soil biological data and the data inventory fundamentally lacks comprehensive data for most organism groups:

- Lumbricidae (best data packet): data from 97 PSM sites (of 795), representative for grassland, agricultural sites and forests but allocation of PSM sites to further levels of habitat types only rudimentarily possible, gaps for some federal states;
- Enchytraeidae: data from 60 PSM sites; no regional representativeness (e.g. little data from Eastern Germany, Bavaria or Rhineland-Palatinate);
- Collembola, Oribatida, and other soil invertebrates in general: no data sets from PSM sites;
- Microbes: thus far no suitable data for biodiversity assessment.

In summary, the results indicate that the data basis on the occurrence of the most important soil organism groups is at date not sufficient to be used for a monitoring of GMO.

Additional sampling on representative PSM sites is recommended while all PSM sites need to be classified at least according to a standardized list of habitat types beforehand.

Up to now, there is little experience regarding the assessment of GMO-related effects in the field [39, 46–50]; see also the overview compiled by Theißen and Russel [51]. Based on the experience and considerations presented so far a technical committee of the Association of German Engineers VDI developed a guideline with proposals for identifying organism groups within the scope of GMO monitoring [27, 28]. To assess the biological soil quality, taxa should be selected according to the following criteria:

- important ecological function within the ecosystem, representativeness for a trophic level;
- close association with the mineral soil or the litter layer;
- sufficient species diversity to differentiate between sites;
- good taxonomical and ecological knowledge;
- wide distribution in Central Europe;
- existing standardized sampling methods;
- potential for routine use, e.g. regarding simplified determination methods;
- availability of data from existing monitoring programs.

At least four different taxa should be used that facilitate the inclusion of different trophic (epigeic–endogeic) as well as functional (feeding type) levels. Which taxa are most appropriate depends on the region and the land use type of the site to be monitored. Details are given in [27, 28].

### Representativeness of PSM sites

The establishment of a biodiversity monitoring of GMO requires generating a data basis representative for Germany, to enable the derivation of reference values for relevant habitat types. To assess the representativeness of PSM sites/data for a monitoring of effects of GMOs on the soil biocoenosis, the classification of habitat types according to Riecken et al. [34, 37] was used. The PSM sites are representative regarding different basic soil types [45] and also according to different natural regions and ecoregions in Germany. Considering all ecoregions of Germany only three types, Swabian cuesta, Swabian alps and the Frisian marshlands are not well represented. In these three ecoregions, cultivation of genetically modified plants is highly unlikely. According to Riecken et al. [33, 36], 21 of 44 basic habitat types can be assumed as relevant for soil organism diversity [6]. Almost all PSM sites belong to three following listed relevant habitat types:

**Table:** 

Habitat code	No. of PSM sites	Abbreviated name
33	351	Arable land
34	102	Grassland
43	242	Deciduous forest

There is a generally good representativeness of German PSM sites for arable land. However, this statement is limited to the basic habitat type “arable and fallow land” [34]. Due to their life form, for soil organisms, additional site-specific parameters (soil properties, nutrient supply, moisture, etc.) are relevant for their distribution. These parameters are reflected in the further subdivision of habitat types (2nd and 3rd level). For an allocation of PSM sites to certain habitat types, often detailed data are missing, e.g. regarding bedrock (lime, silicate, sand, etc.) or general nutrient availability (extensive, species rich, intensive, nutrient rich, species poor, etc.). A standardized and detailed data collection should be pursued since the distribution of soil animals shows the strongest correlation at lower levels of site classification [38, 39].

### Exposure of PSM towards GMO

**Fig. 4:**
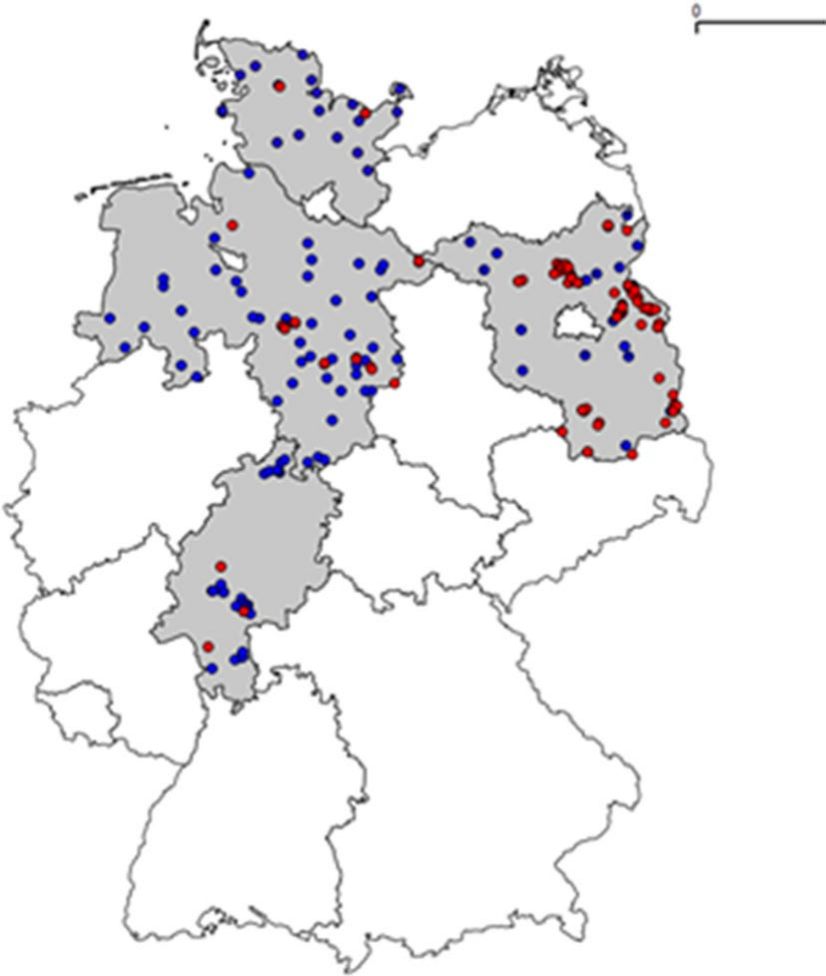
Distribution of PSM sites (*blue dots*) and MON810 field sites (*red dots*) on exemplary federal states (Brandenburg, Hesse, Lower Saxony and Schleswig–Holstein). Data sources: environmental agencies of the federal states for the PSM sites; public location register of the German Federal Office of Consumer Protection and Food Safety (BVL) for the MON810 field sites

In Fig. 4, the distribution of PSM sites and MON810 field sites on the four exemplary federal states of Brandenburg, Hesse, Lower Saxony and Schleswig–Holstein are displayed.

**Fig. 5:**
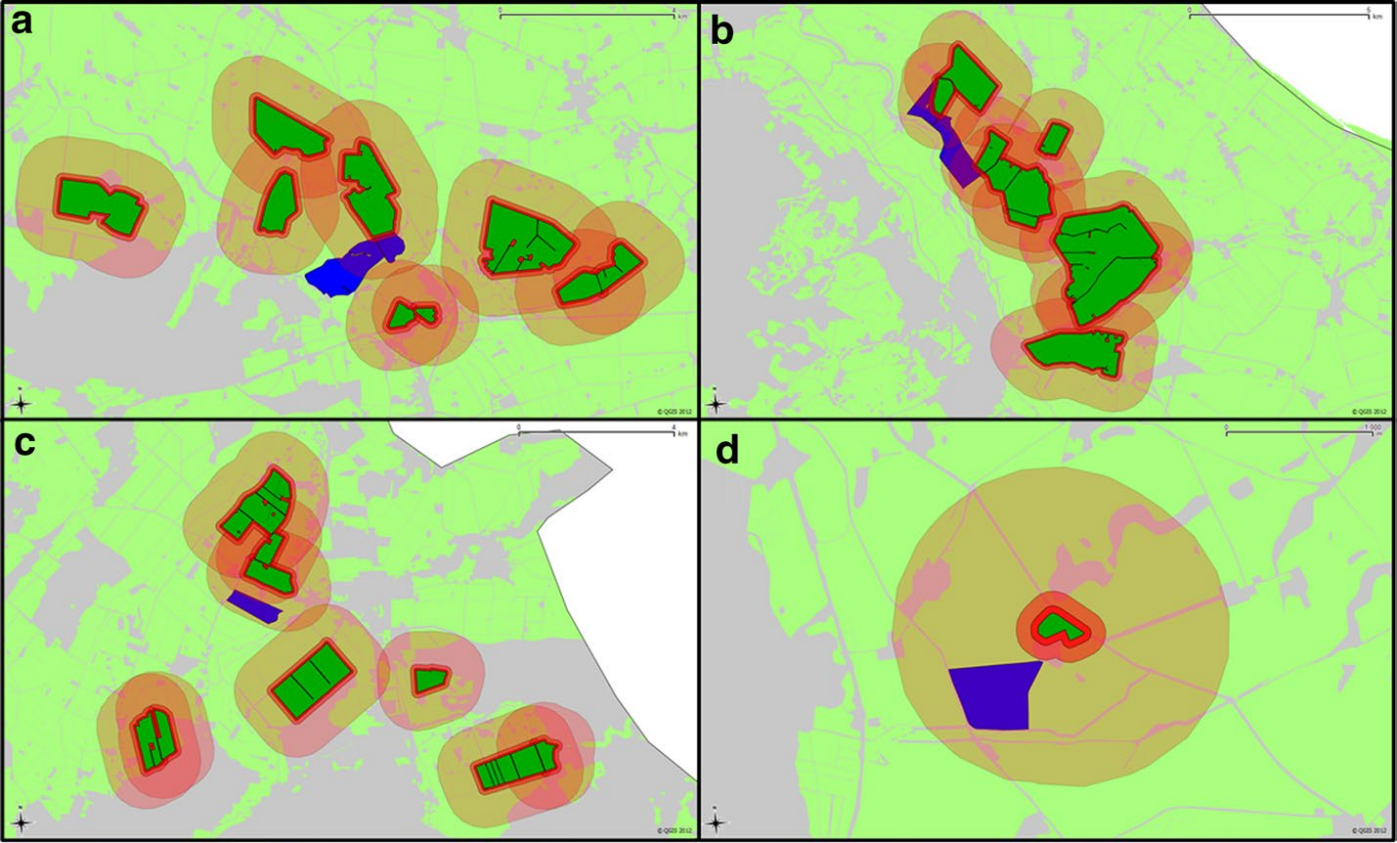
Potential exposure of PSM sites in Brandenburg towards MON810 pollen from 2005 to 2008. *Blue* Parcel of land containing a PSM site. *Dark green* MON810 field sites. *Red buffers* Assumed pollen dispersal radii of 50, 100 and 1000 m. **a** PSM site of Gusow in the administrative district of Maerkisch-Oderland; **b** PSM site of Rathsdorf in the administrative district of Maerkisch-Oderland; **c** PSM site of Neuholland in the administrative district of Oberhavel; **d** PSM site of Augustenfelde in the administrative district of Uckermark. Data sources: environmental agency of Brandenburg for the Integrated Administration and Control System (IACS) data and PSM sites; Public Location Register of the German Federal Office of Consumer Protection and Food Safety (BVL) for the MON810 field sites

Brandenburg is the federal state with the most intensive cultivation of MON810 in Germany in the past (ca. 1350 ha in 2007). In particular, four PSM sites within the administrative districts of Maerkisch-Oberland, Oberhavel and Uckermark were probably already exposed to the adjacent GMO cultivation in the past (Fig. 5) when assuming a pollen dispersal radius of 1000 m. Referring to the total number of 109 PSM sites located in the four considered federal states, this is a share of 3.7 %. Referring to the total number of PSM sites located within Brandenburg (20), this is a percentage of 20 %. In the remaining three exemplary federal states with a much lower MON810 cultivation in the past, none of the PSM sites appear to have already been exposed to MON810 pollen in the past. The cultivation of MON810 on PSM sites could not be observed albeit it is generally permitted. From this example, it can be assumed that with a future intensification of GMO cultivation in Germany, more PSM sites on arable land would likely become exposed towards GMO.

### Practicability

To run a PSM site system in a sustainable way and on a long term, several issues of practicability have to be taken into account and clarified in general.

To be able to use PSM sites as non-influenced reference sites in the future, measures would be needed to prevent these sites from exposure towards GMO. These would need to include prohibition of GMO cultivation on PSM sites and the definition of GMO-free buffer zones around these sites as well as a minimum time lag since the last possible exposure towards GMO. Such spatial and temporal distances would have to be fixed on a scientific basis as much as possible but may vary, e.g. according to GMO crop type and expected exposure pathways. If GMO crops have been previously cultured at this site, it cannot be used as a reference site in the monitoring program, i.e. a historically GMO use has to be excluded definitely. Of course, a site at which GMO cultivation has been practised for several years could serve as a “positive” GMO reference site—but just for one specific GMO. In any case, the sites should have a sufficient size (minimum: 1 ha), and should be easily accessible, which means that the owners have to be integrated in the monitoring program in a contractually embedded long-term approach. Also, the difficulty to ensure a stable land use on each reference PSM site over time has to be realized.

### Conclusion regarding the current status of the German PSM program

The information compiled in this chapter can be summarized as follows: The German PSM is suitable for monitoring potential effects of GMO on soil organism communities, because:

- There is already an existing network of 795 PSM sites in Germany.
- Soil organism communities are an important protection goal and could be exposed to GMO via different pathways.
- A theoretical concept to utilize soil biodiversity data in monitoring concepts is available.
- Basic parameters needed for such a monitoring program (e.g. the characterization of site and soil properties as well as the history of the sites) are already measured at the PSM.
- The PSM is representative for the different biogeographic regions in Germany as well as for the distribution of agricultural land potentially to be used for GMO crops.
- As exemplified by an example site from Brandenburg, it is highly likely that PSM is located in the same area as sites cultivated with GMO crops.
- Finally, issues of practicability do not contradict the use of PSM as reference sites or (less likely) as a positive reference site for the assessment of potential side-effects of GMO crops.

This approach is already proposed for the assessment of soil quality, e.g. in the Netherlands (BISQ), where both structural and functional endpoints are utilized for various organism groups [52]. The development of references on GMO uninfluenced sites is necessary for the assessment of the impact of GMO on soil biocoenoses. If PSM sites are to be used for this purpose, it would be necessary to protect them from exposure towards GMO.

However, when discussing these issues, several shortcomings of the use of PSM have been identified. To overcome these, several modifications of the PSM program have to be performed. They will be listed and discussed in the following chapter.

### Outlook: formulation of possibilities to expand or adapt the federal permanent soil monitoring program and/or complementary monitoring modules for the GMO monitoring

Based on the experiences made in the course of this research, the following recommendations can be given how to adapt the German permanent soil monitoring program for the assessment of potential side-effects of GMO. Therefore, the following recommendations will also be useful for biological soil monitoring in general. For a minimum set of sites, it is recommended to use a grid, based on the distribution of existing PSM sites. The sites should be evenly distributed among all federal states and should be nationally coordinated to ensure a harmonized approach. The major habitat types (arable land, grassland, deciduous and coniferous forests), integrating several subtypes [6, 34, 37], with ten sites each (i.e. roughly 160–200 sites), should be covered and sampled within the course of 5 years. Standardization regarding both, point in time and method of sampling should strongly reduce variability and strengthen data comparability. The sites should be representative regarding the soil factors in those ranges relevant for Germany: pH value, soil texture, surface soil conditions (humus form, litter layer/mineral soil), and geographical regions. Finally, site selection should allow integration into European monitoring programs.

Recommendations of parameters for a minimum soil characterization (all measurements should be performed according to available ISO guidelines or other comparable standards); [14, 45, 53, 54]: pH value (CaCl2, KCl), SOM content, cation exchange capacity, soil dry mass, texture, soil density. With respect to the biological monitoring focus, nitrogen content, C/N ratio, water holding capacity and humus form (especially for forest sites) should also be recorded. Additionally, the following site properties should be recorded: site history (land use, prior samplings), exact geographical location (coordinates), current land use type, climate data (at least: mean annual and monthly air temperature and precipitation; annual course of surface soil temperature), ground-water level, anthropogenic impact (concentrations of common contaminants, e.g. heavy metals, PAH); physical stress (management practice, compaction, fertilization, erosion, etc.).

Recommendations for a methodological standard for biological monitoring comprise the organism groups of Oribatida, Collembola, Lumbricidae, Enchytraeidae and the diversity of microorganisms. This list is in fact an expansion of a list developed for general soil biological monitoring in the EU-project ‘ENVASSO’ [55]. Sampling should be seasonally matched (spring/autumn) and performed according to available ISO guidelines. Vertical distribution between litter and mineral soil layer should be addressed where appropriate, and sampling should be repeated with a frequency of 3–5 years to get a chronological update of possible changes.

The data raised in such an improved biological soil monitoring program can thus be utilized to fill existing data gaps regarding the occurrence of soil organism taxa at different habitat types. Subsequently, the biological soil-quality assessment approach presented above can be subjected to a validation step and then be implemented for routine practical application.

Any deviation from the reference values determined on PSM sites needs to be evaluated according to previously determined threshold values (Fig. 5). So far there is no regulation regarding effects on the soil biocoenosis in the German Federal Soil Protection Act or other laws, but only precautionary, trigger and action values for substances in the German Federal Soil Protection Ordinance (i.e. certain concentrations of single chemicals must not be exceeded in soils with a certain land use [56]).

The usability of data for a nationwide monitoring of GMO requires a standardized collection and management of data among all federal states to facilitate a central evaluation of the results (e.g. landscape- or culture-based [39, 57, 58]). In this context, it has to be noted that:

1. The various federal state PSM programs currently differ in their structure regarding data collection, management and evaluation;
2. The data will become more valuable through long-term, comparable measurements. Hence, continuity regarding data management should be established through a centralized coordination.
3. Uniform and comparable data necessary for the evaluation within a GMO monitoring need to be analysed and discussed;
4. Minimum standards for data flow and management need to be provided.
5. Qualified and independent committees need to be nominated for performing nationwide data evaluation. The competent authority could resort to the already existing working group for the evaluation of PSM data, consisting of representatives from both federal agencies and federal state authorities.
6. The question of the financial contributions (e.g. GMO commercializing companies or agencies) for the preparation and use of nationwide monitoring data must be practically and adequately solved.

## Additional file

10.1186/s12302-015-0057-2 In the Supplement Material section, the complete report of the project in German language is provided.
