# Supplementary material for: Possibilities of using the German Federal States’ permanent soil monitoring program for the monitoring of potential effects of genetically modified organisms (GMO)
Source: Environ Sci Eur. 2015 Oct 23;27(1):26. doi: 10.1186/s12302-015-0057-2 (PMC5044936; doi:10.1186/s12302-015-0057-2)
Supplement: Supplementary file 1 — 10.1186/s12302-015-0057-2 In the Supplement Material section, the complete report of the project in German language is provided. [file 12302_2015_57_MOESM1_ESM.pdf]

Jörg Römbke, Stephan Jänsch, Martina Roß-Nickoll und Andreas Toschki

Nutzungsmöglichkeiten der Boden-Dauerbeobachtung der Länder für das Monitoring der Umweltwirkungen gentechnisch veränderter Pflanzen

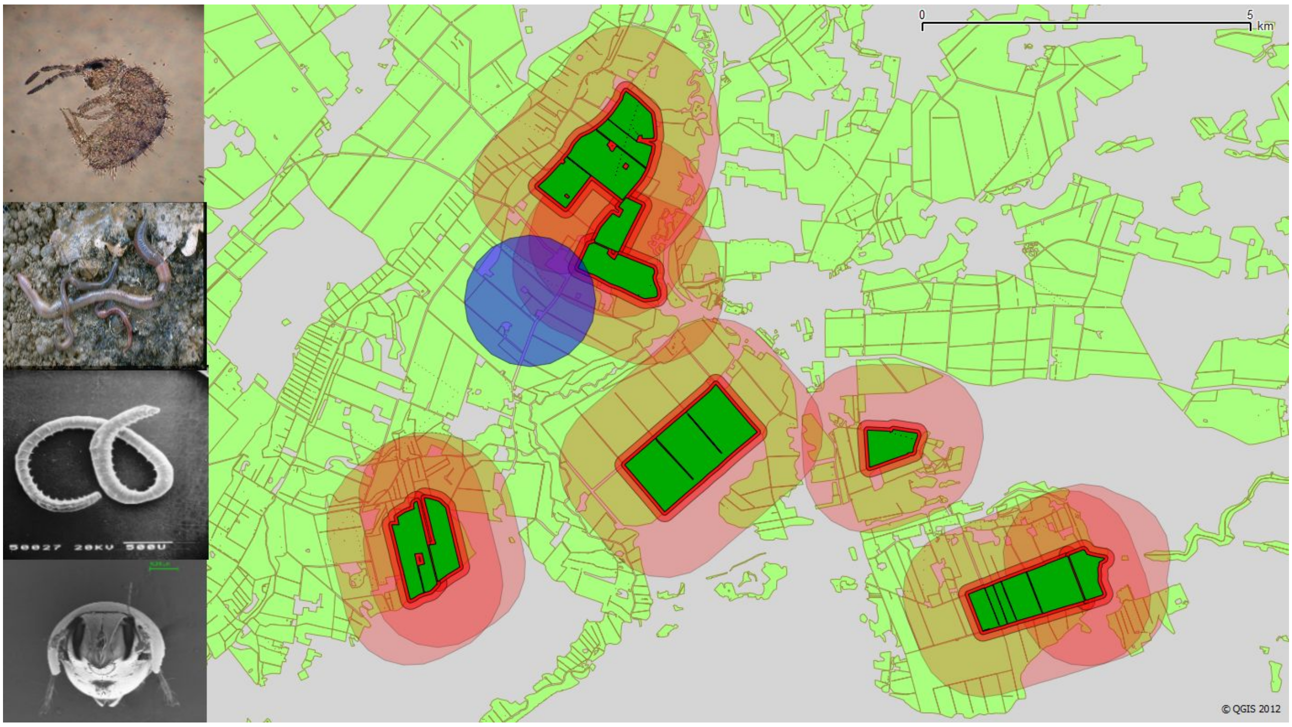

Bundesamt
für Naturschutz

BfN-Skripten 369

2014

Nutzungsmöglichkeiten der Boden-Dauerbeobachtung der Länder für das Monitoring der Umweltwirkungen gentechnisch veränderter Pflanzen

Jörg Römbke
Stephan Jänsch
Martina Roß-Nickoll
Andreas Toschki

Bundesamt
für Naturschutz

Titelbilder: Exposition von Acker-BDF gegenüber MON 810-Anbau und angenommene Pollenausbreitung (ECT); Bildleiste links: Bodenorganismen (RWTH Aachen u. gaiac)

Adressen der Autorin und der Autoren:

Dr. Jörg Römbke, Dr. Stefan Jänsch
ECT Oekotoxikologie GmbH
Böttgerstraße 12-14, 65439 Flörsheim
E-Mail: j-roembke@ect.de
s-jaensch@ect.de

Dr. Martina Roß-Nickoll
RWTH Aachen
Institut für Umweltforschung
Worringer Weg 1, 52074 Aachen
E-Mail: ross@bio5.rwth-aachen.de

Dr. Andreas Toschki
gaiac
Forschungsinstitut für Ökosystemanalyse und -bewertung
Kackerstraße 10, 52072 Aachen
E-Mail: toschki@gaiac.rwth-aachen.de

Fachbetreuung im BfN:
Dr. Wiebke Züghart — Fachgebiet II 1.3 „Monitoring"

Diese Veröffentlichung wird aufgenommen in die Literaturdatenbank **„**DNL-online**"** (www.dnl-online.de).

BfN-Skripten sind nicht im Buchhandel erhältlich. Eine pdf-Version dieser Ausgabe kann unter http://www.bfn.de heruntergeladen werden.

Herausgeber: Bundesamt für Naturschutz
Konstantinstr. 110
53179 Bonn
URL: www.bfn.de

Der institutionelle Herausgeber übernimmt keine Gewähr für die Richtigkeit, die Genauigkeit und Vollständigkeit der Angaben sowie für die Beachtung privater Rechte Dritter. Die in den Beiträgen geäußerten Ansichten und Meinungen müssen nicht mit denen des institutionellen Herausgebers übereinstimmen.

Das Werk einschließlich aller seiner Teile ist urheberrechtlich geschützt. Jede Verwertung außerhalb der engen Grenzen des Urheberrechtsgesetzes ist ohne Zustimmung des institutionellen Herausgebers unzulässig und strafbar.

Nachdruck, auch in Auszügen, nur mit Genehmigung des BfN.

Druck: Druckerei des Bundesministeriums für Umwelt, Naturschutz, Bau und Reaktorsicherheit (BMUB)

Gedruckt auf 100% Altpapier

ISBN 978-3-89624-104-7

Bonn - Bad Godesberg 2014

Inhaltsverzeichnis

Einleitung ... 8

1 Grundlagen zur Exposition von Bodenorganismen (Ergebnis einer Literaturrecherche) ... 9

1.1 Das Konzept der Bioverfügbarkeit ... 9
1.2 Die Exposition beeinflussende Eigenschaften von Bodenorganismen ... 10
1.3 Verhalten von Bodenorganismen ... 11
1.4 Expositionspfade von GVOs ... 11
1.4.1 Einleitung ... 11
1.4.2 Beschreibung der wichtigsten Expositionspfade: Organismen-bezogener Ansatz ... 12
1.4.3 Beschreibung der wichtigsten Expositionswege: GVO-bezogener Ansatz ... 16
1.4.4 Exposition von Boden-Dauerbeobachtungsflächen gegenüber GVP ... 17
1.5 Zusammenstellung der für die Beobachtung von GVO-Wirkungen relevanten Bodenorganismen ... 18

2 Fachliche Bewertung der Möglichkeiten und Grenzen der Nutzung der Boden-Dauerbeobachtung der Länder für das GVO-Monitoring ... 22

2.1 Das Boden-Dauerbeobachtungsprogramm: Einleitung ... 22
2.2 Die Bo-Info Datenbank ... 23
2.3 Repräsentativität der BDF ... 25
2.3.1 Verteilung der BDF nach Bundesländern und Hauptnutzungstypen ... 25
2.3.2 Verteilung der BDF nach Biotoptypen ... 26
2.3.3 Verteilung der BDF nach Boden- und Standorteigenschaften ... 29
2.4 Verwendbarkeit von BDF als Referenz ... 33
2.4.1 Methodik ... 34
2.4.2 Ergebnisse ... 35
2.4.3 Fazit ... 46
2.4.4 Darstellung der zoologischen Erhebungen auf BDF ... 47
2.5 Fazit: Repräsentativität der BDF, speziell in Hinsicht auf deren Nutzung für bodenbiologische Monitoringprogramme ... 49

3 Entwicklung konkreter Erweiterungs- oder Anpassungsmöglichkeiten der Boden-Dauerbeobachtung der Länder und / oder ergänzender Monitoringmodule für das GVO-Monitoring ... 51

3.1 Konzeptionelle Herangehensweise ... 52
3.2 Einzelne Tiergruppen ... 54
3.2.1 Collembolen ... 55
3.2.2 Oribatiden ... 55
3.2.3 Lumbriciden ... 56
3.2.4 Enchytraeen ... 58
3.3 Repräsentativität der im UBA-Vorhaben erfassten Boden-Biodiversität ... 62
3.4 Fazit: Stand der Referenzwertableitung ... 63
3.5 Vorschläge zur Weiterentwicklung des Monitoring ... 64
3.5.1 Empfehlungen für ein Minimalprogramm zum GVO-Monitoring von Bodenorganismen ... 65

3.5.2 Weitere Empfehlungen 70
4 Prüfung der Datenverfügbarkeit sowie von Möglichkeiten der Datenauswertung für das GVO-Monitoring 72
5 Literatur 74
6 Zusammenfassung 82
7 English Summary 94

Abbildungsverzeichnis

Abb. 1: Von der Gesamtkonzentration im Boden bis zur Wirkung 9

Abb .2: Körpergröße der Individuen häufiger Bodentiergruppen und ihre Zuordnung zu trophischen Gruppen...11

Abb. 3: Verteilung des Bt-Toxin (Beispiel Cry 1ab) im Boden.................................12

Abb. 4: Boden-Dauerbeobachtungsflächen (BDF) in den verschiedenen deutschen Naturräumen, differenziert nach Landnutzung. 23

Abb. 5: Relationale Beziehungen verschiedenen Datenblätter in der Bo-Info Datenbank. 24

Abb. 6: Anteile der Nutzungstypen an der Gesamtlandesfläche der jeweiligen Bundesländer. Anteile der Nutzungstypen an den eingerichteten BDF. 26

Abb. 7: Anteile der BDFs an den Biotoptypen 28

Abb. 8: Verteilung der BDF-Standorte auf die als relevant eingestuften Basis-Biotoptypen der Standardliste der Biotoptypen Deutschlands......................29

Abb. 9: Verteilung der BDF-Standorte auf Bodenbasistypen nach KA5. 30

Abb. 10: Darstellung der ökologischen Raumgliederung Deutschlands in 21 Klassen 31

Abb. 11: Verteilung der BDF-Standorte hinsichtlich der ökologischen Raumgliederungseinheiten 32

Abb. 12: Verteilung der BDF-Standorte hinsichtlich der naturräumlichen Großlandschaften 32

Abb. 13: Fläche des MON810-Anbaus in den deutschen Bundesländern 2005-2008. 34

Abb. 14: Beispiel Bundesländer (BL), für die InVeKoS Datensätze angefragt wurden. 36

Abb. 15: Anteil des MON810-Anbaus in Brandenburg an der gesamt Ackerfläche 36

Abb.16: Lage der Acker-BDF und MON810-Anbau in Brandenburg.........................37

Abb. 17: Feldblöcke mit Acker-BDF und mit MON810-Anbau im Landkreis Märkisch-Oderland 2005-2008...38

Abb. 18: Exposition des Feldblocks der Acker-BDF Gusow im Landkreis Märkisch-Oderland gegenüber MON810-Anbau. 38

Abb. 19: Exposition des Feldblocks der Acker-BDF Rathsdorf im Landkreis Märkisch-Oderland gegenüber MON810-Anbau. 39

Abb. 20: Feldblöcke mit Acker-BDF und mit MON810-Anbau im Landkreis Oberhavel 39

Abb. 21 Exposition des Feldblocks der Acker-BDF Neuholland im Landkreis Oberhavel gegenüber MON810-Anbau 40

Abb. 22: Feldblöcke mit Acker-BDF und mit MON810-Anbau im Landkreis Spree-Neiße. 41
Abb. 23: Feldblöcke mit Acker-BDF und mit MON810-Anbau im Landkreis Uckermark 41
Abb. 24: Exposition des Feldblocks der Acker-BDF Augustenfelde im Landkreis Uckermark gegenüber MON810-Anbau 42
Abb. 25: Lage der Acker-BDF und MON810-Anbau in Hessen 43
Abb. 26: Feldblöcke mit Acker-BDF und mit MON810-Anbau im Landkreis Main-Kinzig-Kreis 43
Abb. 27: Lage der Acker-BDF und MON810-Anbau in Niedersachsen 44
Abb. 28: Lage der Acker-BDF und MON810-Anbau in Schleswig-Holstein. 45
Abb. 29: Feldblöcke mit Acker-BDF und mit MON810-Anbau im Landkreis Schleswig-Flensburg 45
Abb. 30: Exposition des Feldblocks der Acker-BDF Schuby im Landkreis Schleswig-Flensburg gegenüber MON810-Anbau 46
Abb. 31: Darstellung der Standorte mit zoologischen Erhebungen für Lumbriciden und Enchytraeen 49
Abb. 32: Standorte zu denen Daten zu Bodenorganismen vorhanden sind....52
Abb. 33: Prinzip zur Ableitung von Schwellenwerten in Bezug auf Referenzzustände 53
Abb. 34: Schematische Darstellung der Biotoptyp spezifischen Biodiversität am Beispiel eines Ackers....54
Abb. 35: Gegenüberstellung von Ergebnissen diverser Parameter an konventionell bewirtschafteten Standorten mit denen einer Referenzfläche. 64
Abb. 36: Entscheidungsbaum zur standardisierten Auswahl von geeigneten Organismen-Gruppen zum GVO Monitoring. 68

Tabellenverzeichnis

Tab. 1: Standardisierte Datensätze hinterlegt in der Bo-Info Datenbank. 25

Tab. 2: Auswahl relevanter Biotoptypen Deutschlands aus der Standard-Biotoptypenliste Deutschlands 27

Tab. 3: Datenherkunft, -genauigkeit und –abfrage zur Darstellung im GIS-Programm 35

Tab. 4: Tabellarische Darstellung der zur Verfügung stehenden Datensätze zur Zoologie, Mikrobiologie und Vegetation. 48

Tab. 5 Mittlere Abundanzen und Artenzahlen der in Bo-Info erfassten Collembolengemeinschaften..55

Tab. 6: Mittlere Abundanzen und Artenzahlen der in Bo-Info erfassten Oribatidengemeinschaften 55

Tab. 7: Potentielle Zeigerarten und Differentialarten der Oribatiden.........................56

Tab. 8: Erwartete Artenzahl und –zusammensetzung sowie die jeweilige mittlere Abundanz, getrennt nach den vier Landnutzungen 57

Tab. 9: Referenzwert-Vorschlag für die Lumbricidengemeinschaft dreier Ackerbiotoptypen.. 58

Tab. 10: Erwartete Artenzahl und –zusammensetzung sowie die jeweilige mittlere Abundanz, getrennt nach den vier Landnutzungen / Hauptbiotoptypen 59

Tab. 11: Erwartete Artenzahl und –zusammensetzung sowie die jeweilige mittlere Abundanz, getrennt nach zwei Acker-Biotoptypen der 2. Ebene 61

Einleitung

Ziel dieses Vorhabens ist die Prüfung der Frage, ob bzw. wie das durch die Bundesländer betriebene Programm der Boden-Dauerbeobachtungsflächen (BDFs) in das durch die Freisetzungsrichtlinie 2001/18/EG (EC 2001) vorgeschriebene Monitoring-Programm zur Überwachung der Umweltwirkungen gentechnisch veränderter Organismen (GVO) eingebunden werden kann (MIDDELHOF et al. 2006; ZÜGHART et al. 2011). Teilweise zeitgleich hat das dieses Vorhaben bearbeitende Konsortium ein durch das Umweltbundesamt gefördertes Projekt durchgeführt, in dem die auf den BDFs erhobenen bodenbiologischen Daten zusammengestellt wurden. Eine darauf aufsetzende Analyse hat das Erreichen der im vorliegenden Vorhaben angestrebten Ziele erleichtert. Damit ist die Grundlage zur Umsetzung folgender Arbeitspakete gegeben:

- AP 1: Erarbeitung von fachlichen Anforderungen an ein aussagefähiges und wissenschaftlich fundiertes Monitoring der Auswirkungen von GVO auf den Lebensraum Boden, speziell an Ackerstandorten
  - ➢ Insbesondere: Übersicht über potenzielle Wirkungspfade beim Anbau von GVO auf den Boden (Literaturrecherche)
- AP 2: Fachliche Bewertung der Möglichkeiten und Grenzen der Nutzung der Boden-Dauerbeobachtung der Länder für das GVO-Monitoring
  - ➢ Konzept und Stand der BDF in den Ländern und Stand der Methodenharmonisierung und Datenhaltung, inklusive der Erarbeitung aussagefähiger Parameter bzw. Indikatoren
- AP 3: Entwicklung konkreter Erweiterungs- oder Anpassungsmöglichkeiten der Boden-Dauerbeobachtung der Länder und/oder ergänzender Monitoringmodule für das GVO-Monitoring
  - ➢ Darstellung von Ergebnissen am Beispiel des bisherigen und potentiellen Anbaus von MON810 in Deutschland
- AP 4: Prüfung der Datenverfügbarkeit sowie von Möglichkeiten der Datenauswertung für das GVO-Monitoring. Entwicklung eines Modells für den Datenfluss und die Datenhaltung
  - ➢ Vorschlag für die Organisation eines Kooperationsmodells

Im Folgenden werden die Ergebnisse des Vorhabens in der Reihenfolge der APs beschrieben.

1 Grundlagen zur Exposition von Bodenorganismen (Ergebnis einer Literaturrecherche)

1.1 Das Konzept der Bioverfügbarkeit

Hinsichtlich der potentiellen Wirkungspfade ist bei gentechnisch veränderten Pflanzen (GVP) vom Konzept der „Gesamtpflanze“ auszugehen; d.h. es wird nicht die jeweilige gentechnische Veränderung isoliert betrachtet sondern die GVP in ihrer Gesamtheit. Aber unabhängig davon, ob es sich um eine Chemikalie, um das Toxin einer genetisch veränderten Pflanze oder um (totes) Material der GVP insgesamt handelt (im Folgenden auch als „Schadstoff i.w.S.“ bezeichnet): stets reagiert ein Organismus nur auf den Anteil, der für diesen Organismus biologisch verfügbar (bioverfügbar) ist. Die bioverfügbaren Anteile der GVP oder Teile davon hängen dabei von den Standort- und Bodeneigenschaften und verschiedenen Prozessen ab, die sich in Abhängigkeit von der Zeit und von den biologischen Rezeptoren verändern. Dieser Zusammenhang ist in der ISO-Richtlinie 17402 zur Bioverfügbarkeit (ISO 2008a) dargestellt (Abb. 1), wobei dort der neutrale Begriff „concentration“ verwendet wird.

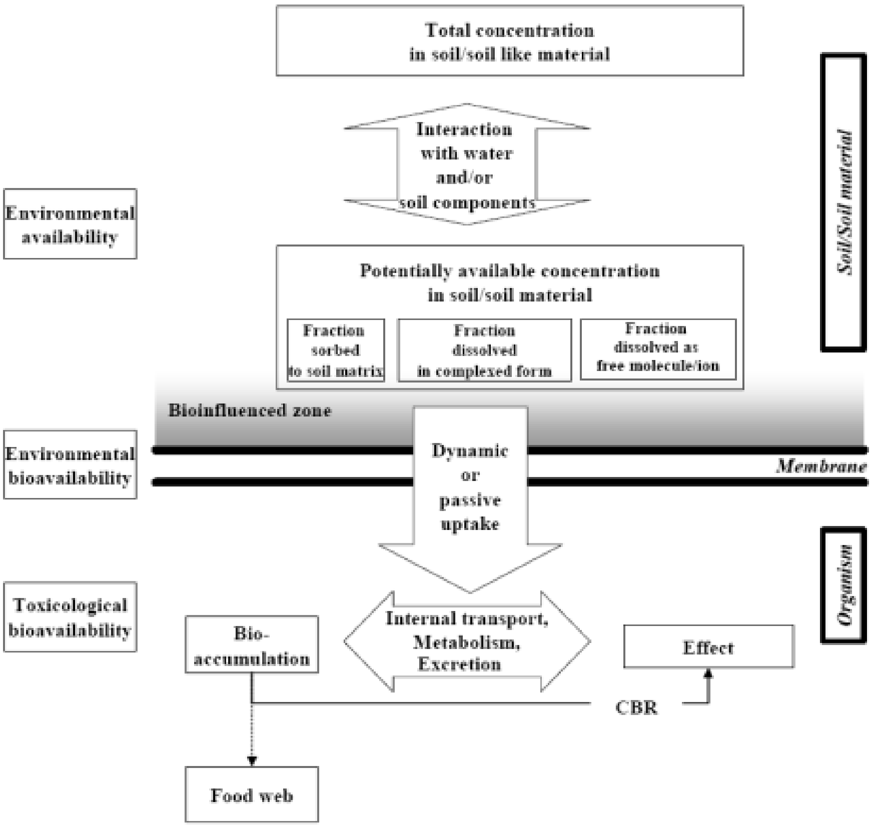

Abb. 1: Von der Gesamtkonzentration im Boden bis zur Wirkung (nach ISO 2008a.)

In Ergänzung zum Konzept der Bioverfügbarkeit wurde in den letzten Jahren der Begriff der „Bioaccessibility“ (eine allgemein verwendete deutsche Übersetzung fehlt bisher) vorgeschlagen (SEMPLE et al. 2004; NAIDU et al. 2008). Als „erreichbare“ Fraktion wird derjenige Anteil eines Schadstoffes i.w.S. bezeichnet, der während der Verdauung in das Köpergewebe gelangt, speziell ins Blut oder die Lymphe. Die erreichbare Fraktion ist da-

bei unabhängig vom jeweiligen Aufnahmepfad via Wasser oder Futter (inklusive der damit zusammen aufgenommenen Bodenpartikel). Obwohl dieser Ansatz für eine Präzisierung des Aufnahmepfads Futter durchaus sinnvoll erscheint, wird er im Folgenden nicht weiter betrachtet, da zum einen für andere Aufnahmepfade solche Präzisierung nicht vorliegt und zum anderen die Bestimmung dieses Anteils sehr artspezifisch ist und somit den Rahmen des hier angestrebten Überblicks übersteigt.

1.2 Die Exposition beeinflussende Eigenschaften von Bodenorganismen

Im Folgenden werden kurz diejenigen morphologischen, physiologischen und das Verhalten betreffenden Eigenschaften diskutiert, die generell die Exposition von Bodenorganismen beeinflussen können (PEIJNENBURG et al. 2012).

Hinsichtlich der Betrachtung der Bioverfügbarkeit werden Bodenorganismen morphologisch in weichhäutige und hartschalige Organismen („soft-bodied & hard-bodied“) eingeteilt. Zur erstgenannten Gruppe gehören Nematoden, Regenwürmer, Enchytraeen sowie einige Insektenlarven während die zweite Gruppe vor allem durch verschiedene Arthropoden (z.B. Spinnen, Milben, Collembolen, Diplopoden, Hundertfüßer sowie Isopoden) repräsentiert wird. Arthropoden verfügen über spezielle Organe zur Aufnahme von Wasser und Sauerstoff, während bei weichhäutigen Tieren diese Stoffe (und teils auch Nährstoffe) vor allem über die Haut in den Körper gelangen. Unabhängig davon können beide Gruppen Schadstoffe i.w.S. auch über das jeweilige Futter aufnehmen.

Innerhalb der Bodenorganismengemeinschaft gibt es eine Vielzahl unterschiedlicher Ernährungsweisen, von denen die Saprophage (d.h. Organismen, die sich von totem organischem Material ernähren), Fungiphage (Pilzfresser), Phytophage (diejenigen, die lebendes Pflanzenmaterial fressen), Zoophage (Räuber) sowie Allesfresser die wichtigsten sind (Abb. 2). Häufiger als noch vor einigen Jahren angenommen ist die Ernährung als Allesfresser: so präferiert die normalerweise als saprophag eingestufte Collembolenart *Folsomia candida* auch Nematoden in Wahlversuchen (CHAMBERLAIN et al. 2006). Gerade bei saprophagen Spezies ist zudem zu beachten, dass sie neben dem toten organischen Material auch die darauf siedelnden Mikroorganismen konsumieren. Des Weiteren ist auch darauf hinzuweisen, dass sich juvenile und adulte Tiere der gleichen Art häufig völlig unterschiedlich ernähren können.

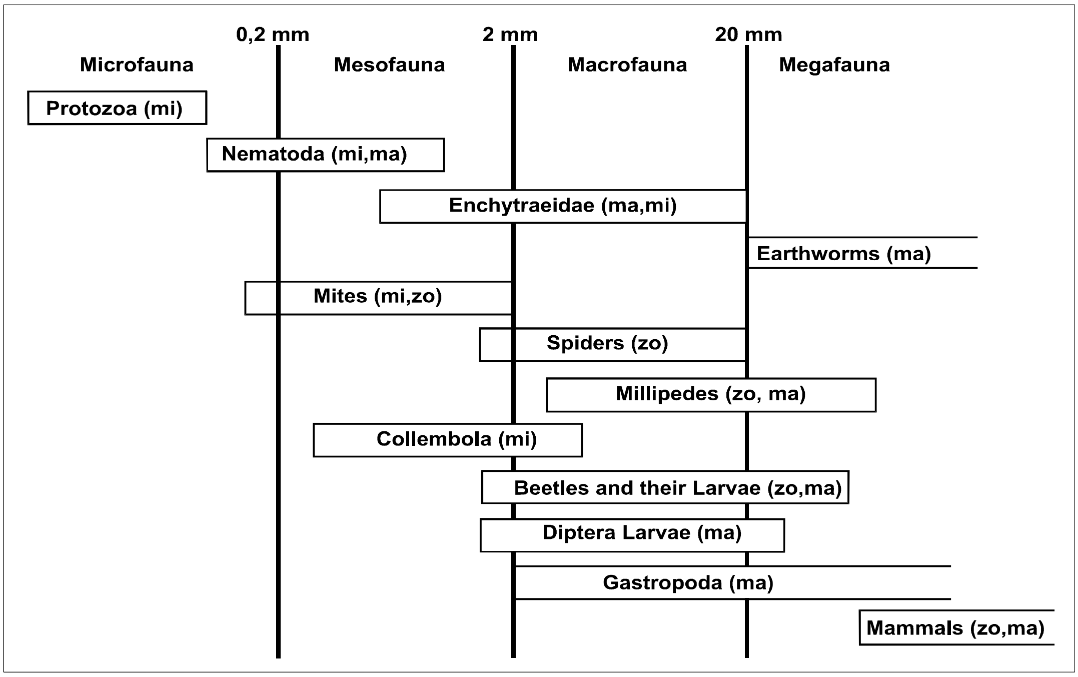

Abb .2: Körpergröße der Individuen häufiger Bodentiergruppen und ihre Zuordnung zu trophischen Gruppen (ma = makrosapro- und makrophytophag, mi = mikrophytophag, zo = zoophag (inkl. nekrophag) (BECK 1993).

1.3 Verhalten von Bodenorganismen

Bei der Darstellung der Exposition von Bodenorganismen gegenüber Schadstoffen i.w.S (z.B. Pestizide, Toxine) wird oft übersehen, dass diese Tiere durch ihr Verhalten die jeweilige Aufnahme des Schadstoffes stark beeinflussen können (RÖMBKE 2008). Ein gutes Beispiel dafür sind Regenwürmer, bei denen sich die Arten auf drei ökologische Gruppen verteilen (Streuschicht- bzw. Mineralschichtbewohner sowie Vertikalgräber; BOUCHÉ 1977), die sich in verschiedenen Bodenschichten aufhalten – und demzufolge in ganz unterschiedlichem Maße exponiert werden. Zudem sind Regenwürmer fähig, aktiv bestimmte Schadstoffe zu vermeiden – eine Beobachtung, die sich inzwischen auch in einem Standardlabortest niedergeschlagen hat (ISO 2008b). Eine ähnliche Situation gibt es für Collembolen, bei denen Verhaltensunterschiede in Gegenwart von Schadstoffen zur Entwicklung eines Standardtests genutzt wurden (NATAL DA LUZ et al. 2008; ISO 2010).

1.4 Expositionspfade von GVOs

1.4.1 Einleitung

Die Definition sowie die Untersuchung von Expositionspfaden dienen dem Zweck, ob bzw. in welchem Ausmaß Bodenorganismen (einzelne Populationen einer Art oder ganze Organismengemeinschaften) während des GVO-Anbaus mit einer der nachfolgend aufgeführten Auswirkungen konfrontiert werden:

- den jeweilig GVO-spezifischen Wirkstoffen (z.B. *Bacillus thuringiensis* cry-Proteine) oder seinen Metaboliten;

- physiologisch GV-veränderten Pflanzenbestandteilen, z.B. einem geänderten Stärkegehalt;
- landwirtschaftlichen Maßnahmen, die ohne GVO-Anbau nicht im gleichen Ausmaß erfolgen würden (z.B. einer erhöhten Herbizidausbringung).

Am Beispiel des Bt-Toxins bzw. der Gesamtpflanzen lassen sich die möglichen Expositionspfade (Abb. 3) aufzeigen. Während die Identifikation von Expositionspfaden in der Realität immer fallspezifisch auf der Grundlage einer Vielzahl verschiedenster Informationen erfolgen muss (vgl. GROSSI-DE-SA et al. 2006), wird im Folgenden versucht, einen generellen Überblick über mögliche Expositionspfade von GVOs für Bodenorganismen zu geben.

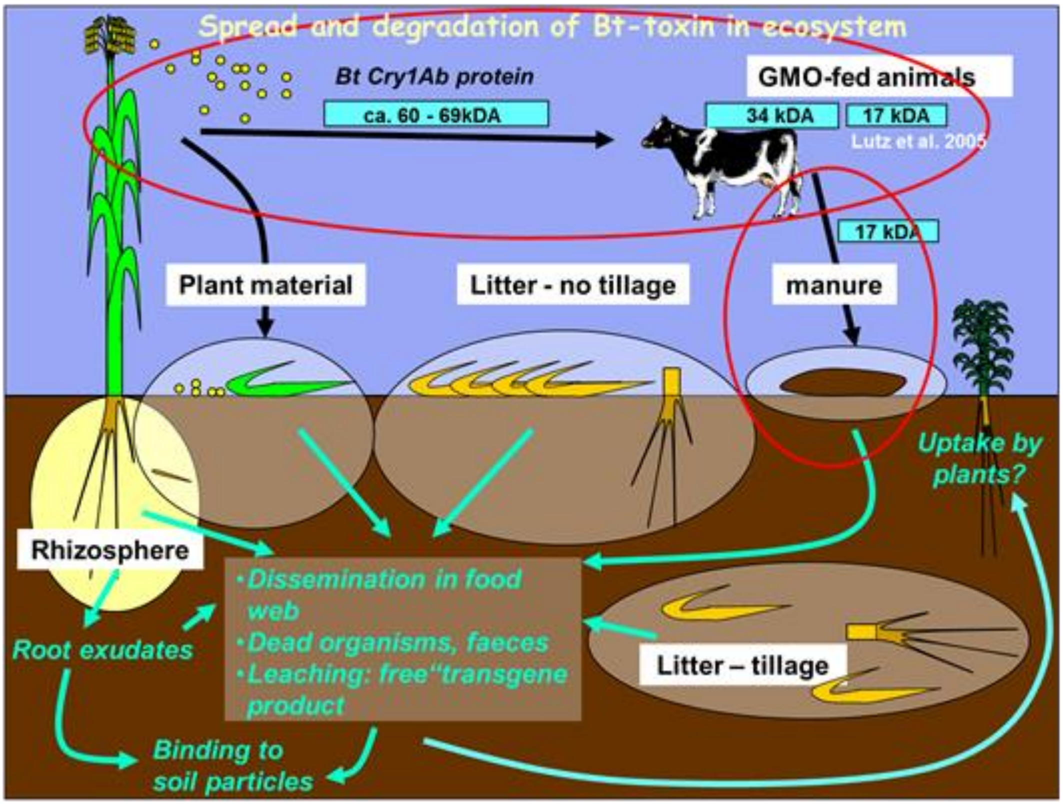

Abb. 3: Verteilung des Bt-Toxin (Beispiel Cry 1ab) im Boden (HILBECK, pers. Mittl..)

1.4.2 Beschreibung der wichtigsten Expositionspfade: Organismen-bezogener Ansatz

Bodenorganismen können bitrophisch durch die GV-Pflanze oder Teile von ihr, einschließlich der darin enthaltenen Transgenprodukte (oft einem Toxin) exponiert werden (HILBECK et al. 2008). Zudem ist eine Exposition auf höheren trophischen Ebenen durch das Transgen-Produkt, dessen Metaboliten, oder die durch das Auftreten dieses Produkts veränderten physiologischen Bedingungen (d.h. veränderten Pflanzeninhaltsstoffen), möglich. Ein Beispiel dafür ist die Weitergabe des Cry3Bb1-Toxins über eine Nahrungskette, bestehend aus Pollen, Collembolen und Carabiden (PRIESNITZ 2011). Auch ist nicht auszuschließen, dass sich GV-Pflanzenteile (Pollen, Nektar, Samen oder totes

organisches Material) oder Transgenprodukte in der Umwelt verbreiten, was zu zeitlich und räumlich veränderten Expositionsszenarien führen kann. Das Transgen könnte auch durch vertikalen Gentransfer (z.B. über Pollen) auf verwandte Pflanzenarten weitergegeben werden, was zu einer deutlichen Ausweitung der Exposition führen könnte: Tiere, die sich von diesen Pflanzen ernähren, würden dann gegenüber dem übertragenen Transgenprodukt exponiert sein. Zudem können Transgenprodukte oder deren Metabolite mit Pflanzeninhaltsstoffen interagieren, was wiederum, z.B. durch eine Veränderung der Qualität des als Nahrung genutzten Pflanzengewebes, Auswirkungen auf Organismen hätte (SAXENA & STOTZKY 2001b; BIRCH et al. 2002; ANDOW & HILBECK 2004). Allerdings muss darauf hingewiesen werden, dass diese komplexeren Expositions- bzw. Wirkungsszenarien bisher selten bei Bodenorganismen untersucht und demzufolge auch nicht nachgewiesen werden konnten. Im Einzelnen werden demnach die folgenden Pfade beschrieben:

- Direkte Exposition der Bodenorganismen gegenüber dem lebenden GVP (Phytophagie);
- Direkte Exposition der Bodenorganismen gegenüber den Wurzelexsudaten der GVP;
- Direkte Exposition von endogäischen Organismen gegenüber dem Transgenprodukt z.B. einem Toxin via Bodenwasser oder adsorbierten Bodenpartikeln;
- Aufnahme von abgestorbenem GVP-Material (Streu, tote Wurzeln, Dung von mit GVP-Material gefütterten Tieren) durch epi- oder endogäische Organismen (Saprophagie);
- Aufnahme von lebendem tierischem Gewebe, das GVP-Material enthält (Zoophagie).

Nach jetzigem Kenntnisstand sind dies die wichtigsten Expositionspfade, wobei allerdings darauf hinzuweisen ist, dass diese Einschätzung vor allem auf den Erfahrungen mit GV-Mais bzw. dem Bt-Toxin beruht. Je nach Entwicklung bzw. Anbau anderer GVOs (speziell der jeweils neuen Eigenschaft (Proteinexpression, Herbizidresistenz usw.) ist diese Liste zu überprüfen. Die bei der genaueren Darstellung dieser fünf Pfade genannten Beispiele werden mit ihren jeweiligen Vor- und Nachteilen genauer in HILBECK et al. (2008) diskutiert. Angaben zur Biologie der einzelnen Organismengruppen wurden, wenn nicht anders angegeben, LAVELLE & SPAIN (2001) entnommen.

Direkte Exposition der Bodenorganismen gegenüber dem lebenden GVP (Phytophagie):

Dieser Expositionspfad ist in der Realität selten, da nur wenige Bodeninvertebraten direkt lebende Pflanzen fressen. Wenn es allerdings doch vorkommt, handelt es sich meist um den Fraß von Insektenlarven an Wurzeln, der auch ökonomisch relevante Ausmaße annehmen kann (z.B. die Schädigung von Eichenbeständen durch das Massenauftreten von Maikäferlarven (z.B. DELB & MATTES 2001)). Wenn dieser Expositionspfad experimentell überprüft wird, ist darauf zu achten, dass das verwendete GV-Material von Pflanzen stammt, die im Freiland zu den bevorzugten Nahrungsquellen des jeweiligen Testorganismus gehören (YU et al. 1997; ROMEIS et al. 2003). Sowohl die Verwendung von normalerweise gemiedenem Material wie auch die von „optimaler“ Nahrung können zu einer Verfälschung der Testergebnisse und damit zu einer Fehleinschätzung dieses Expositionspfades führen.

Direkte Exposition der Bodenorganismen gegenüber den Wurzelexsudaten der GVP:

Zum einen können toxinhaltige Wurzelexsudate Mykorrhiza-Pilze direkt beeinträchtigen (DOLEZEL et al. 2005; MARQUARD & DURKA 2005). Zum anderen stellt die Aufnahme von Wurzelexsudaten ohne Zweifel einen sehr wichtigen Expositionspfad für Bodenorganismen dar, wobei nur wenige Invertebratengruppen diese direkt fressen (z.B. Protozoen, Nematoden). Viel häufiger werden diese Exsudate von Mikroorganismen aufgenommen, die mehr oder weniger direkt mit den betreffenden Pflanzenwurzeln assoziiert sind und dann wiederum den mikrobivoren Invertebraten (speziell wiederum Protozoen und Nematoden, aber auch saprophage Organismen wie Enchytraeen, Collembolen und Bodenmilben z.B. Oribatiden) als Nahrung dienen. Selbst die saprophage Makrofauna kann einen erheblichen Anteil ihres Energiebedarfs über die Mikroorganismen decken, die zusammen mit totem organischem Material aufgenommen werden (siehe unten). Dabei ist die Auftrennung der einzelnen Expositionspfade im hochkomplexen Wurzelumfeld schwierig und bisher nur selten versucht worden (SAXENA & STOTZKY 2001a; VERCESI et al. 2006).

Direkte Exposition von endogäischen Organismen gegenüber einem Transgenprodukt z.B. ein Toxin via Bodenwasser oder adsorbierenden Bodenpartikeln:

Dieser Expositionspfad ist mit Ausnahme der Räuber für alle Bodeninvertebraten relevant, allerdings in sehr unterschiedlichem Maße. Direkt trifft dies für alle weichhäutigen Tiere zu (z.B. Nematoden, Regenwürmer, Enchytraeen, Insektenlarven etc.), da diese über ihre oft mit einer Mukusschicht bedeckte Hautoberfläche im Porenwasser gelöste Stoffe bis zu einer bestimmten, gruppen- bzw. artspezifischen Grenze direkt aufnehmen können. Eine Aufnahme von Stoffen, die bereits an Bodenpartikeln absorbiert sind (entweder organische, häufig sehr komplexe Moleküle wie z.B. Huminsäuren, oder Tonmineralien), kommt dagegen in der Realität eher weniger vor. Dabei ist zu beachten, dass diese Aussagen sehr von den – nicht als konstant anzunehmenden – Bodeneigenschaften, speziell dem pH-Wert oder der Kationenaustauschkapazität abhängen. Bei hartschaligen Tieren findet die Aufnahme aus dem Porenwasser oder von Bodenpartikeln nur an bestimmten Körperstellen statt, z.B. den Intersegmentalfurchen von Asseln oder den Gelenkhäutchen von Insekten oder Spinnentieren.

Die Testung von mikrobiellen Surrogat-Protein, das in eine Bodenprobe eingemischt wird, gehört zu den häufigsten Testansätzen bei der ökotoxikologischen Risikobeurteilung (z.B. von Formulierungen, die das Btk-Toxin enthalten (= *Bacillus thuringiensis kurstaki*)). Besser ist die Verwendung von Bt-Toxin, das aus der entsprechenden GV-Pflanze extrahiert wurde und dann in den Boden eingebracht wird (z.B. AHL GOY et al. 1995). Allerdings kann durch den Extraktionsprozess die Struktur des Proteins und damit dessen Verfügbarkeit verändert werden; d.h. die Exposition würde nicht mehr der realen Freilandsituation entsprechen. Daher ist die Durchführung von Freilandstudien (oder unter zumindest freilandähnlichen Bedingungen wie z.B. in Terrestrischen Modell-Ökosystemen (SCHÄFFER et al. 2010)) zu bevorzugen (vgl. BECK et al. 1999; PAULUS et al. 1999).

Aufnahme von abgestorbenem GV-Material (Streu, tote Wurzeln, Dung von mit GVP-Material gefütterten Tieren) durch epi- oder endogäische Organismen (Saprophagie):

Dieser Expositionspfad ist derjenige, der in den bisher durchgeführten Tests am häufigsten untersucht wurde. So wurden z.B. Regenwurmtests im Labor durchgeführt, in denen GVP-Material direkt in den Boden eingemischt worden war (SAXENA & STOTZKY 2001a; VERCESI et al. 2006). Des Weiteren fanden Untersuchungen zum Verbleib des Bt-Toxins unter realitätsnahen Anbaubedingungen statt (z.B. BAUMGARTE & TEBBE 2005; ICOZ & STOTZKY 2008; ICOZ et al 2008).

Einen Sonderfall stellt die Exposition von Bodenorganismen gegenüber dem Bt-Toxin im Dung von mit GVO-Mais gefütterten Tieren dar, die in der Realität eher selten auftreten dürfte. Dies liegt neben der weitgehenden Umsetzung bei der Darmpassage (GRUBER et al. 2011) nicht zuletzt daran, dass dieser Dung normalerweise eher von darauf spezialisierten Fliegen und -käfern abgebaut wird als von Bodenorganismen (HANSKI & CAMBEFORT 1991; OECD 2010). Letztere greifen unter europäischen Bedingungen erst zu einem sehr späten Zeitpunkt des Abbauprozesses ein (HOLTER 1979). Die Überdauern der durch Dung ausgebrachten Bt-Toxine im Boden ist sehr gering (GRUBER et al. 2012), so dass dieser Expositionspfad zu vernachlässigen ist. Dies gilt auch für die Aufnahme eines Toxins durch andere Pflanzen aus dem Boden, die zwar ebenfalls theoretisch zu einer Exposition von Bodenorganismen führen kann. Allerdings wird diese Möglichkeit schon im Zusammenhang mit der Exposition durch den GVO selbst abgedeckt werden. Zudem fehlen bisher aus der Literatur Belege, dass dieser Expositionspfad zu einer Gefährdung für Bodenorganismen führen könnte.

Aufnahme von lebendem tierischem Gewebe, das GVO-Material enthält (Zoophagie):

Rein zoophage Tiere sind unter den Bodenorganismengruppen nicht sehr häufig (viele bekannte weitgehend obligate Räuber wie Hundertfüßer, Laufkäfer, Kurzflügelkäfer oder Spinnen werden eher zur Makrofauna der Bodenoberfläche gerechnet). Die wichtigsten Räuber im Boden gehören zu verschiedenen Familien der Nematoden sowie den Raubmilben (Gamasina). Im Bodenkompartiment wurden zu diesem Expositionspfad bisher kaum Untersuchungen durchgeführt. Allerdings sind Hinweise zur Relevanz dieses Pfades, d.h. möglichen Wirkungen auf höheren trophischen Ebenen, den wenigen vorliegenden Freilandstudien mit Btk-Formulierungen zu entnehmen (z.B. BECK et al. 1999).

Quantifizierung der jeweiligen Exposition:

Unabhängig vom jeweiligen Expositionspfad ist für die Beurteilung einer möglichen Gefährdung der Bodenorganismen die Quantifizierung des aufgenommenen Schadstoffs notwendig. Dies kann entweder durch die Messung der verabreichten oder aufgenommenen Futtermenge oder der Menge bzw. Konzentration im umgebenden Substrat (d.h. dem Boden (= Totalkonzentration) oder im Boden-Porenwasser geschehen. Im Falle eines Toxins gehört die aufgenommene Menge im Bodenorganismus zur bevorzugten Methode. Doch ist dabei zu beachten, dass beim Bezug auf den Gesamtorganismus (oft aus Gründen der geringen Größe der Organismen die einzige Möglichkeit) nichts darüber bekannt ist, inwieweit der Schadstoff i.w.S. sich an einem Ort befindet, an dem Wirkungen ausgelöst werden können oder ob er, z.B. in einem Ausscheidungsorgan, weitgehend

neutral gespeichert ist. Allerdings wurde eine solche Messung im Körpergewebe bei Bodeninvertebraten bisher nur sehr selten durchgeführt (z.B. SAXENA & STOTZKY 2001a). Bei der Wirkung einer toxischen Substanz ist bekannt, dass die Toxikokinetik und Toxikodynamik getrennt betrachtet werden sollten (JAGER et al 2011, ASHAUER et al. 2011). Dazu sollten die relevanten internen Konzentrationen im Organismus gemessen und berücksichtigt werden. Diese sind abhängig von äußeren Einflüssen (z.B. pH-Wert, TOC-Gehalt etc.). Eine Interpolation dieser Konzentrationen auf Freiland relevante Konzentrationen ist nachträglich möglich (MCELROY et al. 2010).

1.4.3 Beschreibung der wichtigsten Expositionswege: GVO-bezogener Ansatz

Während im vorhergehenden Kapitel die jeweiligen Expositionspfade in einer Art beschrieben wurden, wie sie generell bei Schadstoffen i.w.S. in der Ökotoxikologie üblich ist (vgl. HILBECK et al. 2008), wird im Folgenden weitgehend der Darstellung der Exposition durch GVOs im Rahmen des BfN-Vorhabens „Gentechnik in der Landwirtschaft" (ESCHENBACH & WINDHORST 2009) gefolgt, ergänzt durch weitere Informationen. Demnach können drei transportabhängige Typen der Exposition unterschieden werden, wobei im Genindikator-Projekt vor allem der 2. Transportweg behandelt wurde:

1. Transport von mit GVP-Material kontaminierten landwirtschaftlichen Produkten (z.B. Streu oder Gülle von mit GVP gefütterten Tieren) auf landwirtschaftliche Flächen (sowohl GVP-Anbauflächen als auch andere Standorte).
2. Transport durch Tiere, die sich zunächst zu den GVO-Flächen hinbewegen, dort die Diasporen/GV-Produkte „abholen" und an einen anderen Ort transportieren (endozoochor oder exozoochor).
3. Transport von Diasporen/GV-Produkten von den Anbauflächen weg, z.B. durch Wind).

Diese drei Typen werden im Folgenden unter besonderer Berücksichtigung der Exposition von Bodenorganismen genauer diskutiert, wobei sich die Aufnahmeprozesse weitgehend überschneiden können (z.B. im Fall des horizontalen Gentransfers). Abschließend wird dann diskutiert werden, welche dieser Expositionspfade hinsichtlich der Nutzung von BDF im Rahmen des GVP-Monitorings von besonderer Relevanz sind.

Hinsichtlich des 1. Typs ist davon auszugehen, dass bei Anbau von GVP auch deren „Nebenprodukte" (z.B. Maisstreu, Dung oder Gülle von mit GVP gefütterten Nutztieren) in die Umwelt, insbesondere auf landwirtschaftliche Flächen gelangen werden.

Bei der weiteren Darstellung der Exposition der Typen 2 und 3 beziehen sich ESCHENBACH & WINDHORST (2009) vor allem auf die Arbeit von ZÜGHART & BRECKLING (2003), die als die bisher umfassendste Bearbeitung dieses Problemkreises anzusehen ist. Dabei wurden primär die Erfahrungen mit vier GVOs verarbeitet: herbizidresistenter Raps (HR-Raps), Mais mit einer gentechnisch vermittelten Insektenresistenz (Bt-Mais), kohlenhydratveränderte Kartoffeln (KH-Kartoffeln) und virusresistente Zuckerrüben (VR-Zuckerrüben). Allerdings erfolgte die Untersuchung der komplexen Expositionsbedingungen unter Freilandbedingungen bisher nur selten (z.B. SQUIRE et al. 2003), so dass die folgende Auflistung teils auf theoretischen Überlegungen beruht. Nach ESCHENBACH &

WINDHORST (2009) sind demnach die folgenden, für Bodenorganismen relevanten Expositionswege zu unterscheiden.

Horizontaler Gentransfer:

„Durch horizontalen Gentransfer, asexuelle Übertragung von Genen oder Genfragmenten unabhängig von Artengrenzen, können Bodenmikroorganismen im Boden befindliche freie DNA der GV-Pflanze aufnehmen und in ihr Genom integrieren und das Merkmal ausprägen. Dadurch sind Veränderungen der Artenzusammensetzung und Abundanz der Bodenmikroorganismen und des Funktionsgefüges möglich. Horizontaler Gentransfer wird als seltener Prozess eingestuft" (EFSA 2005).

Auswilderung und Durchwuchs:

„Bei Vorhandensein verschiedener Transgene einer Kulturart in einem Gebiet können durch Akkumulation und Neukombination der Transgene unvorhergesehene Wirkungen auftreten" (ZÜGHART & BRECKLING 2003). Veränderte Merkmalsausprägungen können neue Expositionswege eröffnen und eine verstärkte Ausbreitungsfähigkeit bewirken. Insbesondere wenn sich das Anbaugebiet mit dem Herkunftsgebiet deckt und die Kulturarten an die Klimabedingungen gut angepasst sind, können sich stabile Populationen der GVO außerhalb der Kulturflächen etablieren (Auswilderung, Verwilderung). Es kann auf Kulturflächen in den Folgejahren zum Auflaufen von fortpflanzungsfähigen Individuen aus der Bodensamenbank kommen, insbesondere bei sekundärer Dormanz der Samen (Durchwuchs)". Dabei können die Samen z.T. viele Jahre überdauern und nach Jahren auf dem Ackerschlag oder z.B. in Ruderalflächen auflaufen.

Pollenausbreitung:

„Die Pollenausbreitung stellt einen wesentlichen Expositionsmechanismus dar. Wichtige Faktoren sind die Pollengröße, die Überlebensfähigkeit der Pollen, Blühzeitpunkte, Windgeschwindigkeit und -richtung, Bestandsgrößen, räumliche Anordnung der Felder in Bezug zur Hauptwindrichtung sowie Umwelt- und Landschaftsbedingungen" (HALSEY et al. 2005, MESSÉAN et al. 2006, SQUIRE et al. 2003).

1.4.4 Exposition von Boden-Dauerbeobachtungsflächen gegenüber GVP

Dieser Bericht fokussiert sich auf das Boden-Dauerbeobachtungsprogramm und somit konkrete Flächen (BDF). Diese Flächen sind potentiell gegenüber GVP exponiert, was deren Nutzen in einem Monitoring-Programm entscheidend beeinflussen kann und somit zu berücksichtigen ist. „Durch den Anbau von GVP können sich Veränderungen in der landwirtschaftlichen Praxis ergeben (z.B. verändertes Spritzverhalten, Verschiebung der Aussaatzeitpunkte), die wiederum Veränderungen in den betroffenen Ökosystemen hervorrufen können (z.B. GRAEF et al. 2007)". Während potentielle Wirkungen auf den Anbauflächen selbst unstrittig sind, gibt es je nach Anbaukultur - Raps, Mais, Kartoffel etc. - unterschiedliche Vorstellungen, in welchen Entfernungen von Anbauflächen noch Wirkungen des GVP auftreten können.

Soweit keine GVP auf dem BDF angebaut werden, sind als potentielle Expositionspfade gegenüber GVP der Pollenflug, aber auch die Einbringung von Dung, Gülle etc. zu nennen. Im vorliegenden Vorhaben wird nur der Pollenflug als Pfad mit möglichem erheblichem Einfluss berücksichtigt, da die anderen genannten Pfade aufgrund der jeweils

räumlich und zeitlich begrenzten Ausbringung sowie der starken Veränderungen (inklusive Abbau) des GVP-Materials in Substraten wie Dung oder Gülle als weniger relevant eingeschätzt werden.

Hinsichtlich der Ausbreitung von Pollen variieren die Literaturangaben von wenigen Metern bis hin zu mehreren Kilometern (ZÜGHART & BRECKLING 2003; BIOSICHERHEIT 2010, HOFMANN et al. 2008a). In diesem Vorhaben werden drei Distanzen, 50, 150 und 1000 m als Ausbreitungs- und Wirkszenarien berücksichtigt.

1.5 Zusammenstellung der für die Beobachtung von GVO-Wirkungen relevanten Bodenorganismen

Eine erste Zusammenstellung zu diesem Thema wurde, allerdings mit stark methodischem Schwerpunkt, von HILBECK et al. (2008) durchgeführt. Demnach stehen theoretisch 46 Labortestverfahren, 8 Halbfreilandmethoden und 2 Freilandstudien für die Untersuchung der Wirkung von GVOs auf Bodenorganismen zur Verfügung. In Hinsicht auf die zu betrachtenden Organismengruppen wurden Regenwürmer, Collembolen und Asseln als die geeignetsten Testorganismen identifiziert, wobei die jeweilige ökologische Relevanz, Exposition und Praktikabilität der Handhabung ausschlaggebend war. Allerdings bezog sich diese Schlussfolgerung primär auf die Laborebene. Ausgehend von einer weitergehenden Analyse der aus der Chemikalien- bzw. Pestizidbeurteilung zur Verfügung stehenden Testverfahren schlugen RÖMBKE et al. (2010) vor, eine Testbatterie zu erarbeiten, mit der nicht nur die verschiedenen Expositionspfade, sondern auch die wichtigsten trophischen Gruppen der Bodenorganismengemeinschaft abgedeckt werden. Für diesen Zweck könnten sowohl bestehende Verfahren modifiziert (z.B. mit Regenwürmern, Collembolen oder Raubmilben) als auch neue Tests entwickelt werden (z.B. mit Isopoden). Im Gegensatz zum bisherigen Vorgehen ist allerdings fallspezifisch, d.h. je nach der Umwelt, in der GVOs angebaut werden, eine jeweils andere Art der ausgewählten Gruppe einzusetzen. Dabei kommen die Autoren zu folgendem Schluss: „Preferably, the same test method can be used for different species of the same organism group, e.g. representing different regions or environments within Europe. The main challenge will be to increase the number of species from these groups beyond the existing standard ecotoxicological test species in order to account for differences of the receiving environments, behavioural types and exposure pathways (mainly via feeding) more adequately."

Im Zusammenhang mit der in diesem Vorhaben bearbeiteten Freilandebene sind die genannten Gründe für die Organismenauswahl zwar ebenfalls relevant, doch sollte zudem die Sensitivität der Organismen unter Freilandbedingungen in die Entscheidungsfindung eingehen. Daher wird im Folgenden kurz auf die Wirkung von GVOs im Freiland eingegangen. Bisher wurde nur einmal eine standardisierte Methode in einer Freilandstudie mit GVOs (Bt-Mais bzw. Cry1Ab) eingesetzt. Dabei ging es um deren Wirkung auf den Streuabbau (CORTET et al. 2006). Diese Studie was Teil des EU-ECOGEN-Projekts, in dem neben dem integrativen Parameter Streuabbau auch Wirkungen auf verschiedene Bodenorganismengruppen im Freiland untersucht wurden. Die Ergebnisse dieses Teils von ECOGEN lassen sich wie folgt zusammenfassen (BIRCH et al. 2007):

- Es wurden keine Wirkungen des GVO auf die mikrobielle Gemeinschaft des Bodens, ausgewählte Gruppen der Bodenfauna (z.B. Collembolen und Schnecken) oder den Streuabbau festgestellt (vgl. auch ZWAHLEN et al. 2007).
- Auf den Bt-Maisflächen wurden vorübergehende negative Effekte auf die Zahl der Protozoen und Nematoden festgestellt (in Gewächshausversuchen lag deren Zahl aber höher als in Böden von konventionell bearbeiteten Äckern).
- Generell unterschied sich der Einfluss von Bt-Mais auf die Bodenorganismengemeinschaft nicht von den Auswirkungen der jeweiligen Pflanzensorte, der Bodenbearbeitung oder des Pestizideinsatzes

Zu einem ähnlichen Schluss kamen PRIESTLEY & BROWNBRIDGE (2008) in einer zweijährigen Freilandstudie, in der die Wirkung des gleichen Toxins auf Collembolen untersucht wurde. Nach Auswertung verschiedener Freilandstudien mit Collembolen kamen THEIßEN & RUSSELL (2009) zu dem Schluss, dass es keine generellen Tendenzen hinsichtlich möglicher Wirkungen auf diese Tiere gab. Negative Wirkungen traten, wenn überhaupt, bei einzelnen Arten oder Gruppen auf, wobei im Allgemeinen nicht zwischen direkten und indirekten Effekten von GVOs unterschieden werden konnte. Allerdings ist darauf hinzuweisen, dass neben der geringen Zahl bisher durchgeführter Studien auch deren häufig unzureichendes Design (z.B. nur Betrachtung des isolierten Toxins, zu geringe Dauer, keine Aufschlüsselung der taxonomischen Zusammensetzung der betrachteten Organismengruppen sowie Nichtberücksichtigung weiterer Einflussfaktoren neben dem GVO) die Aussagekraft dieser Studien einschränkt.

Einige mehrjährige Freiland-Studien beschäftigen sich insbesondere mit potenziellen Effekten auf die Artenvielfalt, die Abundanz von Flora und Fauna und dem komplexen Gefüge der Räuber-Beute-Beziehungen“ (z.B. CANDOLFI et al. 2004, LUDY & LANG 2006, TOSCHKI et al. 2007, TOSCHKI 2008). In diesen Studien, in denen ein Untersuchungsstandort über mehrere Jahre untersucht wurde, zeigten sich durchgehend hohe Schwankungen von Populationen einiger Arten über die Jahre. Diese Populationsschwankungen zeigten sich unabhängig von der GV-Pflanze, d.h. sie spiegeln die umweltbedingten, natürlichen Schwankungen von Populationen und den damit verbundenen Varianzen im Freiland wider (vgl. FRENCH et al. 2004, BHATTI et al. 2005, LOZZIA 1999). Effekte von GV-Pflanzen auf Nichtzielarthropoden zeigten sich Art spezifisch jedoch nicht generell durch z.B. Populationsrückgänge einer gesamten taxonomischen Gruppe (vgl. auch BROOKS et al. 2003, HAWES et al. 2003). Einige Art-spezifische Veränderungen konnten auf mit dem GVO Anbau einhergehende Veränderungen der Umweltbedingungen (z.B. Feuchtigkeit) zurückgeführt werden (vgl. TOSCHKI et al 2007, TOSCHKI 2008). Der Vergleich verschiedener Studien ist jedoch nur bedingt möglich, da darin unterschiedliche z.T. nicht standardisierte Methoden verwandt wurden (Versuchsdesign, Plotgröße, Wiederholungszahl, statistische Auswertung, taxonomische Auflösung, etc.). Diese Faktoren können, wie BROOKS et al. (2003) herausstellt, großen Einfluss auf die Ergebnisse haben. Betrachtet man z.B. die Abundanz auf Familienniveau ist die Aufhebung von artspezifischen gegenläufigen Effekten möglich. Das bedeutet, es sind Effekte da, die aber aufgrund der geringen taxonomischen Auflösung nicht gesehen werden können (HOLLAND et al. 2002).

Im Rahmen der Erstellung einer Richtlinie zum Monitoring der Wirkungen von GVO auf Bodenorganismen wurde die Literatur von Dr. David Russell (Senckenberg-Museum Görlitz) ausgewertet (VDI 2012), deren zentrale Aussagen die Relevanz der im vorherigen Abschnitt beschriebenen Expositionspfade belegen. Sie wurden von ihm wie folgt zusammengefasst:

1. Durch GVOs potentiell verursachte Veränderungen der Struktur und Funktion der Bodenmikroflora, insbesondere in der Rhizosphäre und Streuschicht, und die damit einhergehenden Veränderungen der Ressourcen betreffen vor allem die saprophagen und mikrobivoren Bodenorganismen. Diese Auswirkungen an der Basis des Nahrungsnetzes können am besten durch die Berücksichtigung von Taxa niedrigerer trophischer Ebenen erfasst werden.
2. Gleichzeitig können durch die mögliche Weiterreichung im Nahrungsnetz auch Organismen höherer trophischer Ebenen beeinflusst werden, was die gleichzeitige Untersuchung von Taxa höherer trophischer Ebenen und damit die Analyse der Nahrungsnetzstrukturen erfordert.
3. Da sowohl in Labor- als auch in Freilandversuchen das Auftreten von Effekten artabhängig war (z. B. bei Protozoa, Nematoda, Collembola, Acari, Lumbricidae, Isopoda, Carabidae, Araneae) reichen Summenparameter (z. B. die Gesamtabundanz) bei verschiedenen Tiergruppen nicht aus. Untersuchungen sollten definitiv auf der Artebene stattfinden, um so Artenzusammensetzungen und Gemeinschaftsstrukturen auswerten zu können. Eine Ausnahme stellen Nematoden dar, bei denen derzeit eine Bearbeitung auf Artebene nicht möglich erscheint. Aus diesem Grunde erscheint eine Auswertung mit Hilfe etablierten Indices zur Gemeinschaftsstruktur zunächst angemessen.
4. Ob Auswirkungen von GVO beobachtet werden, kann von vielen Faktoren (z. B. Bodenart, pH, Temperatur, Jahr, Saison, Region usw.) abhängen, die zum einen zur Variabilität der biologischen Systeme beitragen und zum andern die potentielle Störgrößen der GVOs beeinflussen (z.B. Abbau oder Bioverfügbarkeit von Bt-Toxinen). Für eine Bewertung der Daten sind daher umfangreiche Referenzuntersuchungen nötig.

Ausgehend von bis hierher skizzierten Erfahrungen und Überlegungen hat in den letzten Jahren eine VDI-Arbeitsgruppe konkrete Vorschläge für die Auswahl von Organismengruppen im Rahmen des GVO-Monitorings gemacht (RUF et al. 2013). Um den Bodenzustand in fachlich angemessener Weise bewerten zu können sollten für ein GVO Monitoring Taxa unter Verwendung folgender Kriterien ausgewählt werden:

- Wichtige ökologische Funktion im Ökosystem, Repräsentanz für eine trophische Ebene;
- Enge Anbindung an die jeweiligen Kompartimente (d.h. Bewohner des Mineralbodens oder der Streuschicht);
- Ausreichend hohe Diversität (Artenvielfalt), um Standorte differenzieren zu können;
- Gute taxonomische und ökologische Kenntnisse;
- Weite Verbreitung in Mitteleuropa;

- Vorhandensein von standardisierten Erfassungsmethoden;
- Potenzial zum routinemäßigen Einsatz (insbesondere Möglichkeiten zur Vereinfachung der Determination);
- Verfügbarkeit von Daten aus bzw. Nutzung in bestehenden Monitoring-Systemen.

Die Auswahl sollte hinsichtlich der veränderten Eigenschaften des jeweiligen GVO und der jeweiligen Exposition der Organismen angepasst werden. Es sollten mindestens vier verschiedene Taxa verwendet werden, die es ermöglichen unterschiedliche trophische (epigäisch-endogäisch) sowie funktionelle Ebenen (Ernährungstyp) einzubeziehen.

2 Fachliche Bewertung der Möglichkeiten und Grenzen der Nutzung der Boden-Dauerbeobachtung der Länder für das GVO-Monitoring

2.1 Das Boden-Dauerbeobachtungsprogramm: Einleitung

Das Ziel der Boden-Dauerbeobachtung ist es, den aktuellen Zustand der Böden zu erfassen und langfristig zu überwachen. Es dient somit (UBA 2011):

1. als Frühwarnsystem für schädliche Bodenveränderungen;
2. als Kontrollinstrument für umweltpolitische Maßnahmen;
3. zur Beweissicherung;
4. als Referenz zur Beurteilung von Bodenbelastungen;
5. als Grundlage für Umweltforschung und Methodenentwicklung.

Einen Überblick zur Lage der BDF in den Naturräumen Deutschlands gibt Abb. 4.

Bislang konzentriert sich die Boden-Dauerbeobachtung auf die Messung von physikalischen und chemischen Messgrößen. Nur in wenigen Fällen werden standardisiert biologische Daten erhoben. Dies spiegelt sich auch in der Methode zur Auswahl von BDF wider, die maßgeblich durch den landesbezogenen Anteil von Bodentypen sowie Nutzungsformen bestimmt wird. Ökologisch relevante Parameter werden derzeit für die Auswahl nicht in angemessener Weise berücksichtigt. Ob die fachliche Anforderung an ein fundiertes Monitoring des Lebensraumes Boden als "Lebensgrundlage und Lebensraum für Menschen, Tiere, Pflanzen und Bodenorganismen" (BBODSCHG 1998, §2) durch das Boden-Dauerbeobachtungs-Programm in seinem derzeitigen Umfang erfüllt wird, ist somit fraglich. Dabei ist zu beachten, dass die Verantwortung für den Betrieb und auch die zu messenden Parameter bei den Ländern liegt.

In einem ersten Schritt wurden im Wesentlichen die Fragen zur Repräsentativität und Aussagekraft der BDF hinsichtlich ihrer Nutzung im Rahmen des GVO-Monitorings bearbeitet. Dazu wurden die Lage der BDF sowie deren jeweilige Eigenschaften (z.B. Biotoptyp, Nutzung, Bodenart etc.) in einer Boden-Informations-Datenbank (Bo-Info) zusammengestellt. Damit ist es möglich, Abfragen zu erstellen und Analysen durchzuführen, die für dieses Projekt zielführend sind. Darüber hinaus stehen in der Datenbank geographische Koordinaten zur Verfügung, mit denen es möglich ist, die Lage der BDF kartographisch mittel GIS darzustellen und mit anderen Kartenwerken zwecks Analyse zu verschneiden.

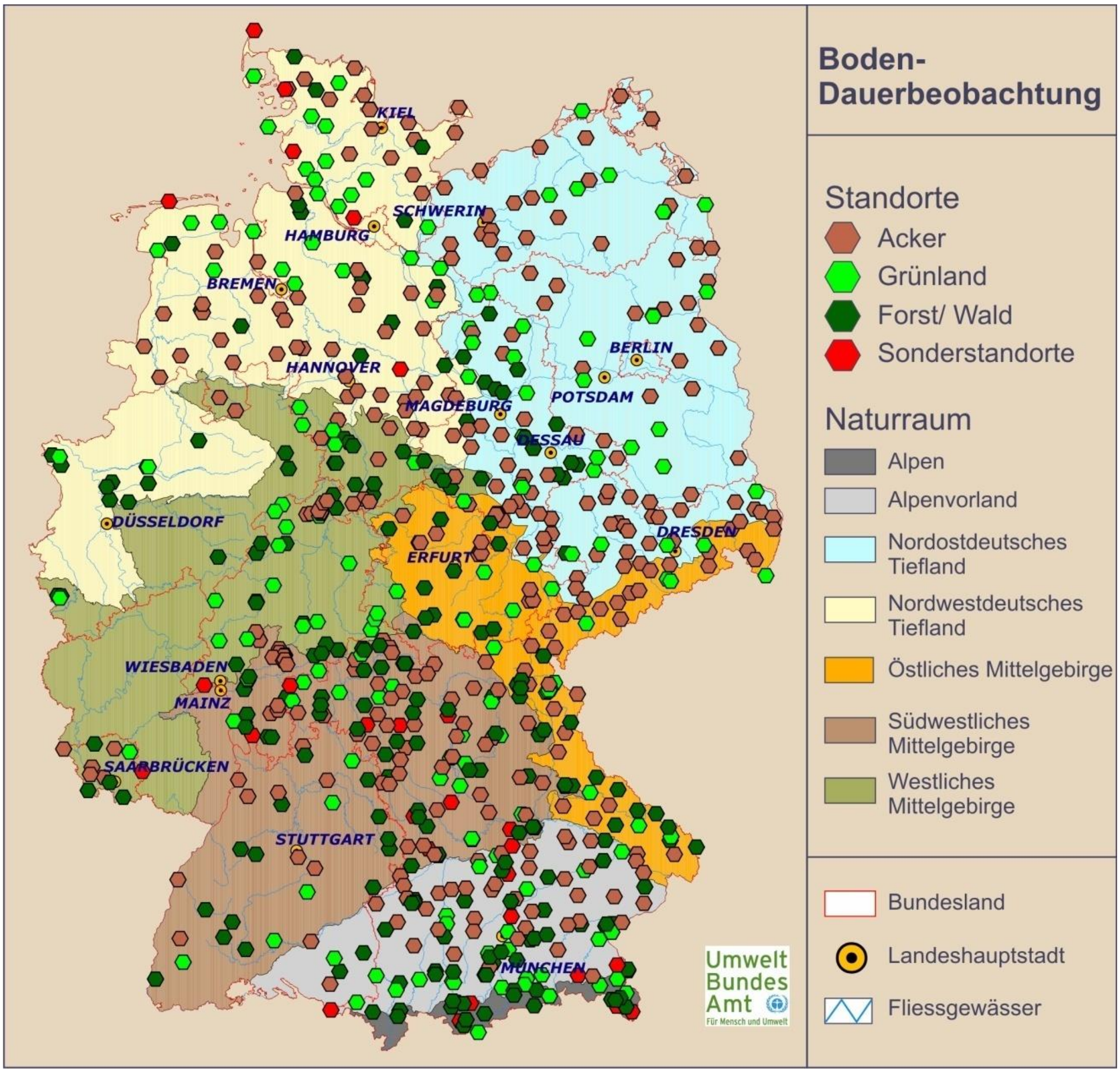

Abb. 4: Boden-Dauerbeobachtungsflächen (BDF) in den verschiedenen deutschen Naturräumen, differenziert nach Landnutzung (RÖMBKE et al. 2012).

2.2 Die Bo-Info Datenbank

Die Bo-Info Datenbank wurde im Rahmen des F & E Vorhabens des Umweltbundesamtes (UBA) "Erfassung und Analyse des Bodenzustandes im Hinblick auf die Umsetzung und Weiterentwicklung der Nationalen Biodiversitätsstrategie" erstellt und auf die Anforderungen im vorliegenden Projekt angepasst (RÖMBKE et al. 2012). Vom UBA wurden bereits alle Daten der BDF zu physikalischen und chemischen Bodeneigenschaften aus den Ländern in einer zentralen UBA-Datenbank zusammengetragen. Da diese Datenbank jedoch nicht auf die Datenführung von bodenbiologischen Datensätzen ausgerichtet war, erfolgte die Erstellung einer separat angelegten Bo-Info Datenbank (Access-Format; Abb. 5). Darin wurden die für Boden-Taxa relevanten Endpunkte (z.B. Artname, Abundanz, Erhebungsmethode etc.) zusammengetragen und in Form einer relationalen Datenbank in Beziehung gesetzt. Die fachlichen Erfordernisse wurden dabei eng mit dem VDI-Arbeitskreis Bodenorganismen (Erstellung der Richtlinie 4331: Monitoring von gen-

technisch veränderten Organismen, speziell zur Wirkung auf Bodenorganismen) abgestimmt. Dabei geht es um die Entwicklung von Methodenstandards zur Erfassung und Beurteilung von Wirkungen auf Bodenorganismen bzw. -gemeinschaften. Diese Fragestellung ist auch zentral für die Aufgabenstellung im vorliegenden Projekt.

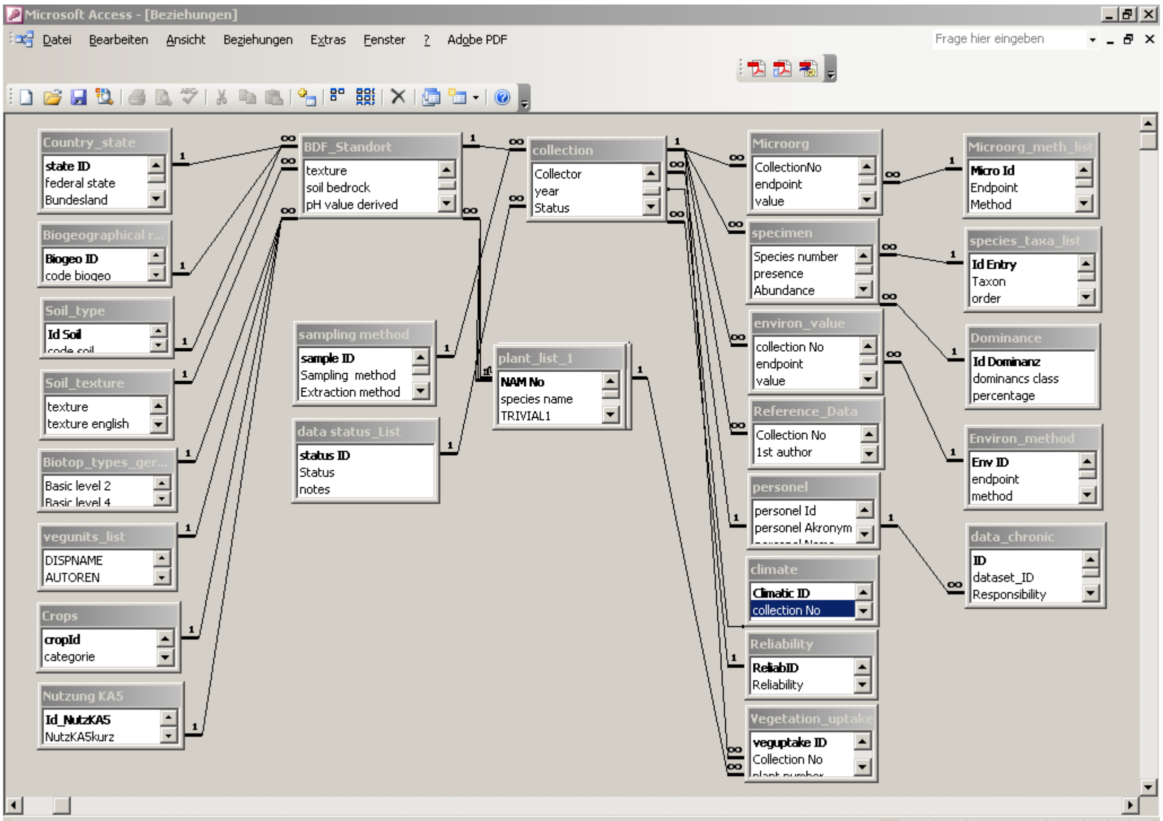

Abb. 5: Relationale Beziehungen verschiedenen Datenblätter in der Bo-Info Datenbank.

Im zweiten Schritt wurden alle für die Bewertung von Biodiversität relevanten Umweltvariablen (z.B. pH-Werte, Bodenart, etc.) aus der UBA-Datenbank in die Bo-Info Datenbank überführt. Darüber hinaus wurden soweit diese vorlagen bodenbiologische Daten aus einzelnen Bundesländern in die Datenbank eingepflegt. Eine Schwierigkeit bestand in der sehr unterschiedlichen Methodik der jeweiligen Länder bezüglich Datenerhebung sowie der differierenden Datenführung (z.B. Access, Excel, etc.). Da die Auswertung auch im vorliegenden Projekt eine bundesweite Betrachtung ermöglichen muss, ist die standardisierte Verwendung von Methoden und Bezugsgrößen unumgänglich. Aus diesem Grunde wurde soweit möglich auf Standardlisten zurückgegriffen, die eine bundesweite Betrachtung ermöglichen. In Tab. 1 sind alle Standardtabellen aufgeführt, die in der Datenbank hinterlegt sind und für eine Analyse verwendet werden können. Jedoch nicht alle BDF verfügen über Daten zu Standardlistenparametern. Dies wird später analysiert und dargestellt.

Tab. 1: Standardisierte Datensätze (Standardtabellen), hinterlegt in der Bo-Info Datenbank.

Tabellenname	Beschreibung Inhalt	Zitat
biotop types	Standard-Biotoptypenliste für Deutschland	Riecken et al. 2003
biogeographical region	Naturräumliche Gliederung Deutschlands/ Naturräume und Großlandschaften	Meynen et al. (1953-1962)
country state	Liste der deutschen Bundesländer	Römbke et al. (2012)
crops	Ackerkultur, Fruchtfolge	AG Boden (1994), KA 4 erweitert
soil type	Bodentypen Deutschlands	AG Boden (1994)
soil texture	Bodenarten	AG Boden (1994)
vegunits_list	Vegetationskundliche Einheiten	BfN (www.floraweb.de)
Nutzung KA5	Nutzungstyp (z.B. Grünland, Acker, Wald etc.)	AG Boden (2005)
plant_list (1-3)	Artnamen der Pflanzen Deutschlands	BfN (www.floraweb.de)
species taxa list	Artnamen (Systematik) der Bodenorganismen	Projektgruppe
dominance class	Dominanzklassen von Organismen (generell)	Engelmann (1978)

2.3 Repräsentativität der BDF

2.3.1 Verteilung der BDF nach Bundesländern und Hauptnutzungstypen

Insgesamt gibt es in Deutschland laut der zentralen UBA-Datenbank (Frank Glante, UBA, pers. Mittl.) 795 BDF (Abb. 4). Die mit Abstand meisten BDF liegen in Bayern vor (289 Standorte). Für Berlin, Bremen und Rheinland-Pfalz werden derzeit keine BDF in der UBA-Datenbank geführt, auch wenn es in diesen Bundesländern BDF-ähnliche Standorte gibt (z.B. in Rheinland-Pfalz gegenwärtig 16; Franz Horak, Staatliches Museum für Naturkunde Karlsruhe, pers. Mittl.).

In Abb. 6 ist vergleichsweise dargestellt, welche Anteile die jeweiligen Hauptnutzungsformen (CORINE-Datensatz) an der Landesfläche besitzen. Daneben ist dargestellt wie die Verteilung der BDF in den jeweiligen Ländern auf die Nutzungstypen verteilt ist. Es ist ersichtlich, dass in den meisten Bundesländern der Anteil an Ackerland sehr stark ist. Dies spiegelt sich durchaus in der Häufigkeit der BDF wider. Als Ausnahme sind, abgesehen von den Bundesländern ohne BDF, die Bundesländer NRW und Hamburg zu nennen in denen Acker-BDF deutlich unterrepräsentiert erscheinen. In einigen Ländern wie z.B. Brandenburg wurde der Fokus auf Äcker und Grünland gelegt, während Wald-BDF unterrepräsentiert sind. In den Ländern Thüringen, Sachsen, Brandenburg sowie Hamburg ist Grünland für die BDF überrepräsentativ berücksichtigt.

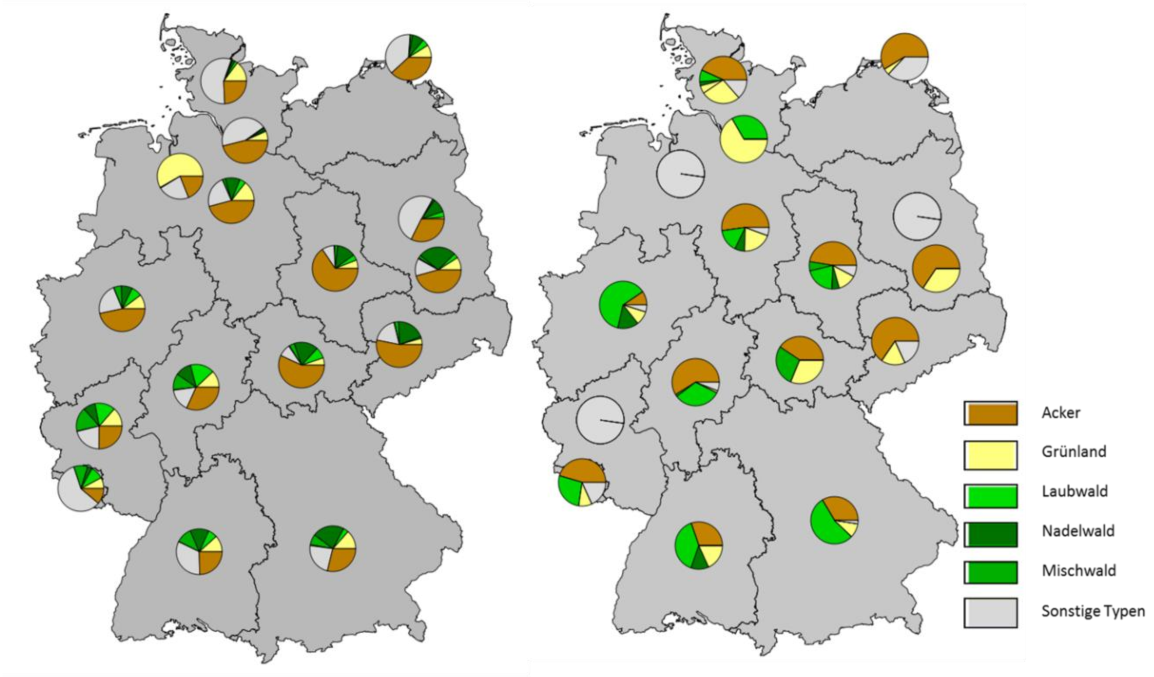

Abb. 6: Links: Anteile der Nutzungstypen (Acker, Grünland und Wald) an der Gesamtlandesfläche der jeweiligen Bundesländer (Datenbasis CORINE-Datensatz); Rechts: Anteile der Nutzungstypen an den eingerichteten BDF im Rahmen des Boden-Dauerbeobachtungs-programmes.

2.3.2 Verteilung der BDF nach Biotoptypen

Um die Repräsentativität der BDF als Grundlage für ein Monitoring der Auswirkungen von GVO auf die Bodenlebensgemeinschaft einschätzen zu können ist es wichtig, die Bezugsgrößen für die Repräsentativität vorab festzulegen. In diesem Zusammenhang ist ein Ansatz notwendig, der die verschiedenen in der Umwelt wirkenden Faktoren integriert (vgl. TOSCHKI 2008, MIDDELHOFF et al. 2006, ROß-NICKOLL et al. 2004). Ansonsten erscheint es unmöglich, die Repräsentativität von BDFs für jeden bodenphysikalischen, biologischen oder klimatischen Faktor (z.B. pH-Wert, Korngröße, Nährstoffgehalte, Feuchte, Klimazone etc.) einzeln zu bestimmen, um statistisch abgesicherte Ergebnisse zu erzielen. Daher wurden die Biotoptypen als integrierende Einheiten als Bewertungsgrundlage für die Repräsentativität genutzt, die mit anderen Größen (z.B. Klimazone, ökologische Raumeinheit bzw. Nutzungsform) kombiniert werden können. Dabei ist zu beachten, dass der Fokus des GVO-Monitorings im Bereich Boden auf den GVO-Anbauflächen liegen wird; d.h. die Nutzungsform Acker steht im Mittelpunkt des Interesses. Aufgrund der möglichen Ausbreitung von Pollen kann aber nicht ausgeschlossen werden, dass die Bodenbiodiversität von anderen Biotoptypen in der Umgebung beeinflusst werden kann.

Biotoptypen differenzieren synökologisch unterschiedliche Boden-Lebensgemeinschaften und integrieren zusätzlich die relevanten Faktoren, die für ihre Differenzierung ausschlaggebend sind (ROß-NICKOLL et al. 2004; TOSCHKI 2008). Maßgebliche Faktoren sind

Bodentyp, Feuchte und Nährstoffversorgung, die auch für die Verteilung der Bodenorganismen wichtig sind. Die hierarchische Gliederung der Standard-Biotoptypenliste für Deutschland bietet darüber hinaus die Möglichkeit einer Auswertung auf verschiedenen ökologischen Skalenebenen (z.B. Repräsentativität für Äcker (Ebene 1) oder für Kalk-Äcker (Ebene 2) etc.). Da sich die Biotoptypenliste auf alle Lebensräume bezieht, wurden vorab nur die für die Diversität der Bodenlebensgemeinschaft wesentlichen Biotoptypen ausgewählt (d.h. alle Biotoptypen der Meere, Gewässer und Felsstandorte wurden ausgeschlossen). In Tab. 2 sind diejenigen 21 Biotoptypen angegeben, mit denen eine Repräsentativitätsanalyse durchgeführt wurde.

Tab. 2: Auswahl relevanter Biotoptypen Deutschlands aus der Standard-Biotoptypenliste Deutschlands (RIECKEN et al. 2003): Basis Typen und die in ihr enthaltenen Untertypen.

Basis Code	Untertypen	Basis Name
07.	16	Salzgrünland der Nordseeküste (Supralitoral)
08.	13	Salzgrünland, Brackwasserröhrichte und Hochstaudenfluren des Geolitorals der Ostseeküste
10	1	Küstendünen
33	26	Acker und Ackerbrache
34	83	Trockenrasen sowie Grünland trockener bis frischer Standorte
35	39	Waldfreie Niedermoore und Sümpfe, Grünland nasser bis feuchter Standorte (ohne Röhrichte und Großseggenrieder)
36	26	Hoch-, Zwischen- und Übergangsmoore
37	7	Großseggenrieder
38	10	Röhrichte (ohne Brackwasserröhrichte)
39	44	Wald- und Ufersäume, Staudenfluren
40	14	Zwergstrauchheiden
41	63	Feldgehölze, Gebüsche, Hecken und Gehölzkulturen
42	20	Waldmäntel und Vorwälder, spezielle Waldnutzungsformen
43	70	Laub (Misch)Wälder und –Forste
44	56	Nadel (Misch) Wälder und –Forste
65	3	Moore der subalpinen bis alpinen Stufe
66	10	Gebirgsrasen (subalpine bis alpine Stufe)
67	6	Stauden- und Lägerfluren der hochmontanen bis alpinen Stufe
68	3	Zwergstrauchheiden der subalpinen bis alpinen Stufe
69	10	Gebüsche der hochmontanen bis subalpinen Stufe
70	5	Subalpine Wälder

351 BDFs, d.h. 44 % aller BDFs, gehören zum Biotoptyp Acker, 242 (30 %) zum Biotoptyp Laubwald (vgl. Abb. 4). Weiterhin liegen 102 Standorte (13 %) im Biotoptyp Grünland. 4 % der Standorte besitzen keine Biotoptypenzuordnung, alle anderen Biotoptypen liegen unter 5 %. Die Verteilung der Biotoptypen in den einzelnen Ländern weicht von diesem bundesweiten Muster zum Teil erheblich ab (Abb. 7).

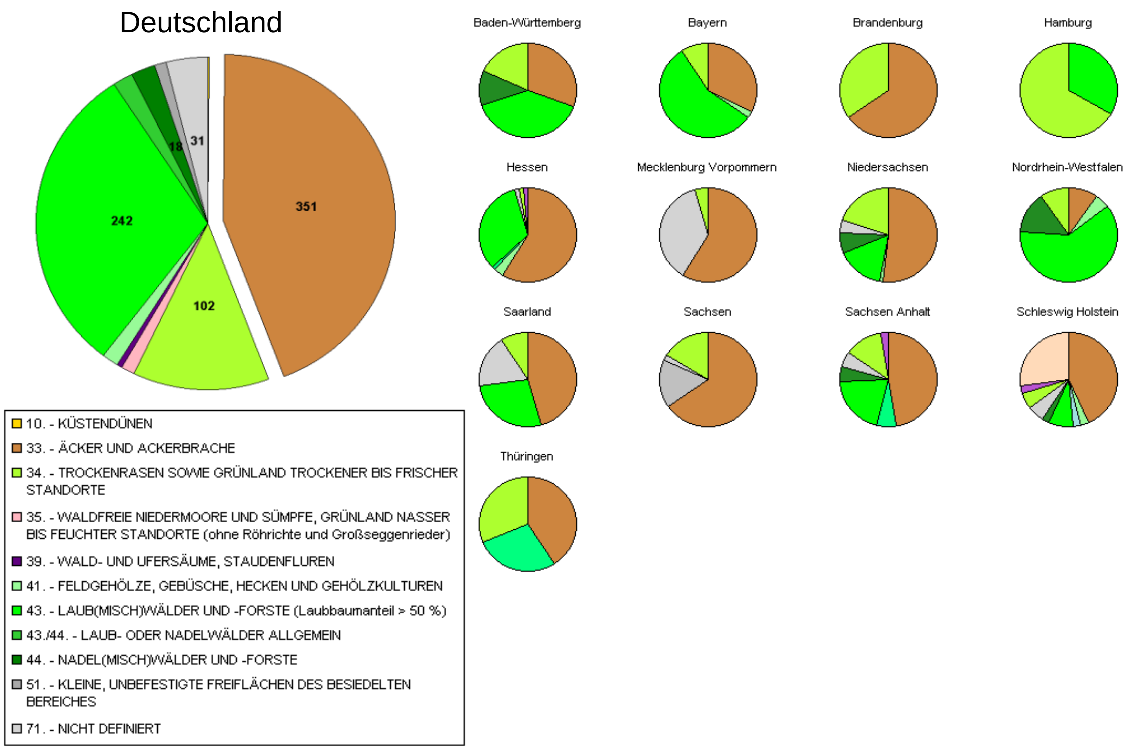

Abb. 7: Anteile der BDFs an den Biotoptypen (Standard-Liste für Deutschland, Basis-Code). Links oben: Gesamt Deutschland, Rechts: für die Bundesländer

In Abb. 8 ist ersichtlich, dass bis dato im Wesentlichen nur drei Biotoptypen durch eine ausreichende Menge BDF-Standorte abgedeckt werden. Dies sind Äcker (351), Grünland trockener und frischer Standorte (102) sowie Laubwälder (242). In einem kleineren Umfang sind auch Nadelwälder (18) berücksichtigt worden. Alle anderen Standorte werden nicht oder nur unwesentlich durch die BDFs abgebildet (vgl. dazu auch Abb. 7).

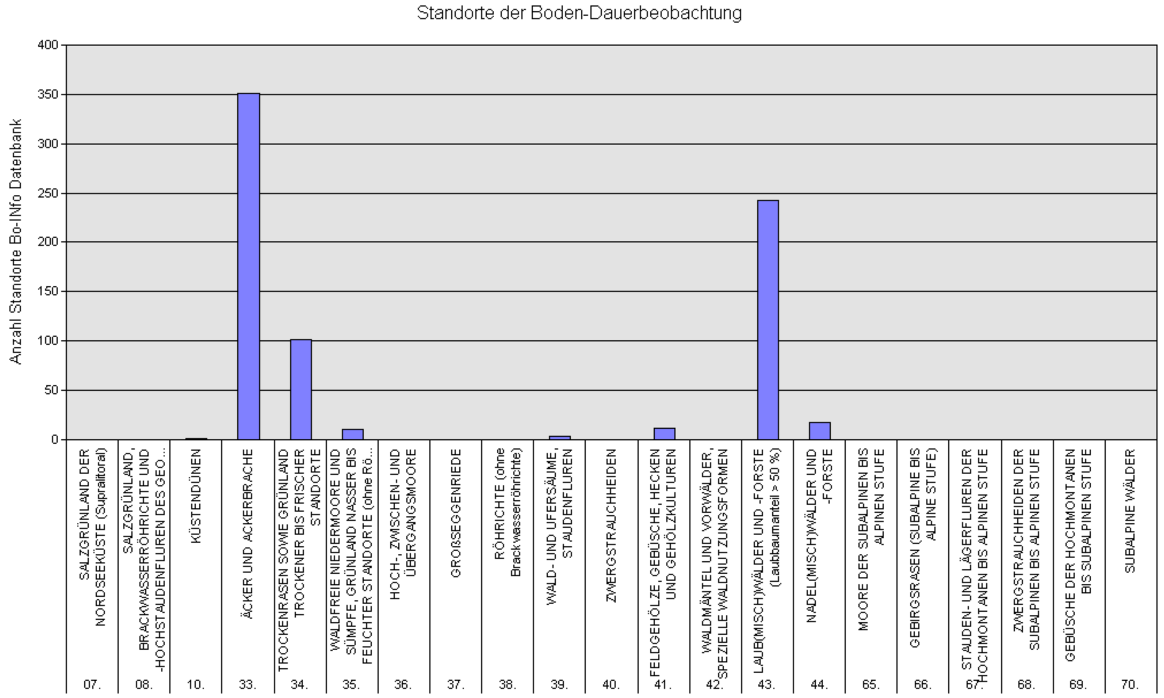

Abb. 8: Verteilung der BDF-Standorte auf die als relevant eingestuften Basis-Biotoptypen der Standardliste der Biotoptypen Deutschlands

2.3.3 Verteilung der BDF nach Boden- und Standorteigenschaften

Entsprechend der Anleitung zur Auswahl von Boden-Dauerbeobachtungsflächen nach Repräsentativität des Bodentyps und der Nutzung (BARTH et al. 2000) im jeweiligen Bundesland ist eine sehr homogene Verteilung der BDF Standorte über verschiedene Bodenbasistypen ersichtlich (vgl. Abb. 9). Am stärksten sind Ah-B-C Böden wie Braunerden, Stauwasserböden und Lessives vertreten. In geringerem Maße, immerhin mit über 25 Standorten vertreten sind Ah-C Böden wie Ranker und Rendzinen, Auenböden, Gleye, natürliche Moorböden und Podsole.

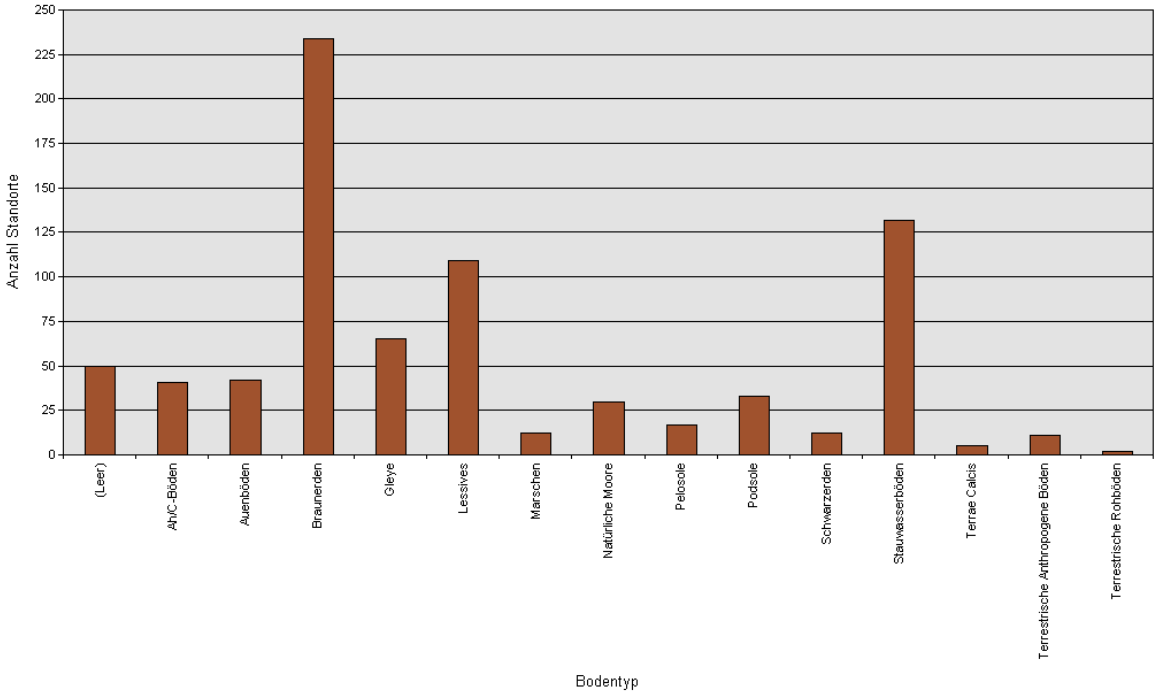

Abb. 9: Verteilung der BDF-Standorte auf Bodenbasistypen nach KA5.

Nach SCHRÖDER & SCHMIDT (2000) wurden anhand von räumlichen Daten zu potentieller natürlicher Vegetation (Zielvariable), Klima, Relief / Topographie und Bodenart ökologische Raumeinheiten für die ökologische Umweltbeobachtung (SCHRÖDER & SCHMIDT 2000) erstellt. Wie in Abb. 10 dargestellt sind die ökologischen Raumklassen räumlich voneinander getrennt und besitzen unterschiedliche Anzahlen von BDF. Die Repräsentativität der BDF hinsichtlich der 21er Klassifikation dieser ökologischen Raumgliederung, ist in Abb. 11 dargestellt. Es zeigt sich, dass die Raumklassen durch die BDF sehr gut abgedeckt und die BDF somit als repräsentativ anzusehen sind. Lediglich drei Raumeinheiten (Klasse 22, 26 und 63) besitzen weniger als 10, alle anderen sind mit mehr als 20 Standorten vertreten. Auch in Bezug auf die naturräumliche Einteilung in Großlandschaften (Gliederung des BfN auf Basis von MEYNEN et al. 1953-62 & IFAG 1979) sind die BDF sehr homogen verteilt (Abb. 12).

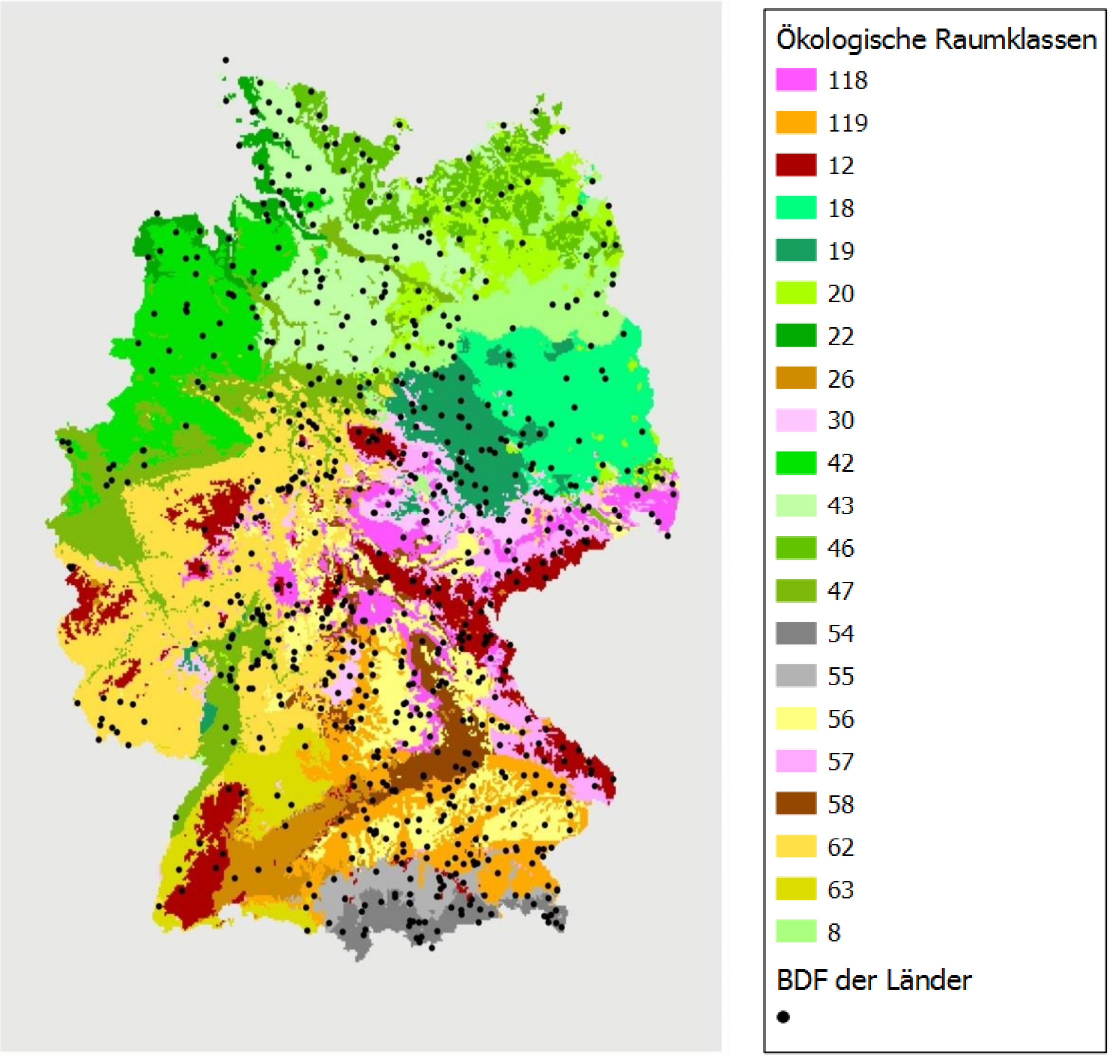

Abb. 10: Darstellung der ökologischen Raumgliederung Deutschlands in 21 Klassen (vgl. SCHRÖDER & SCHMIDT 2000).

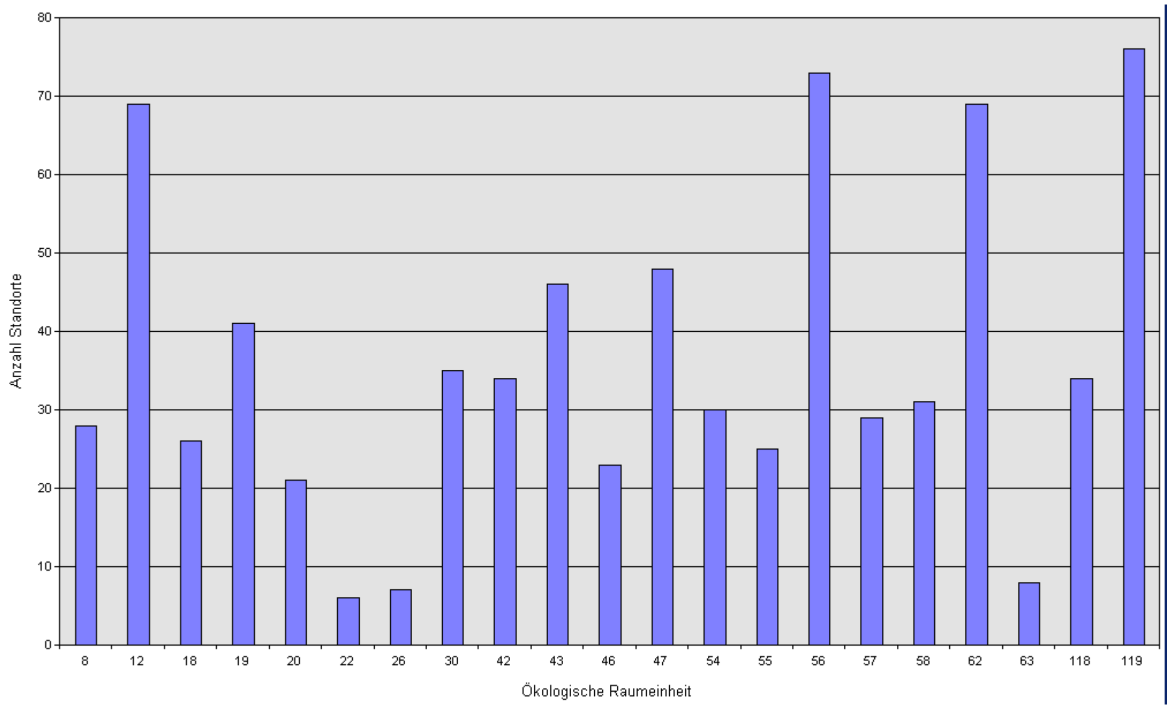

Abb. 11: Verteilung der BDF-Standorte hinsichtlich der ökologischen Raumgliederungseinheiten nach SCHRÖDER & SCHMIDT (2000).

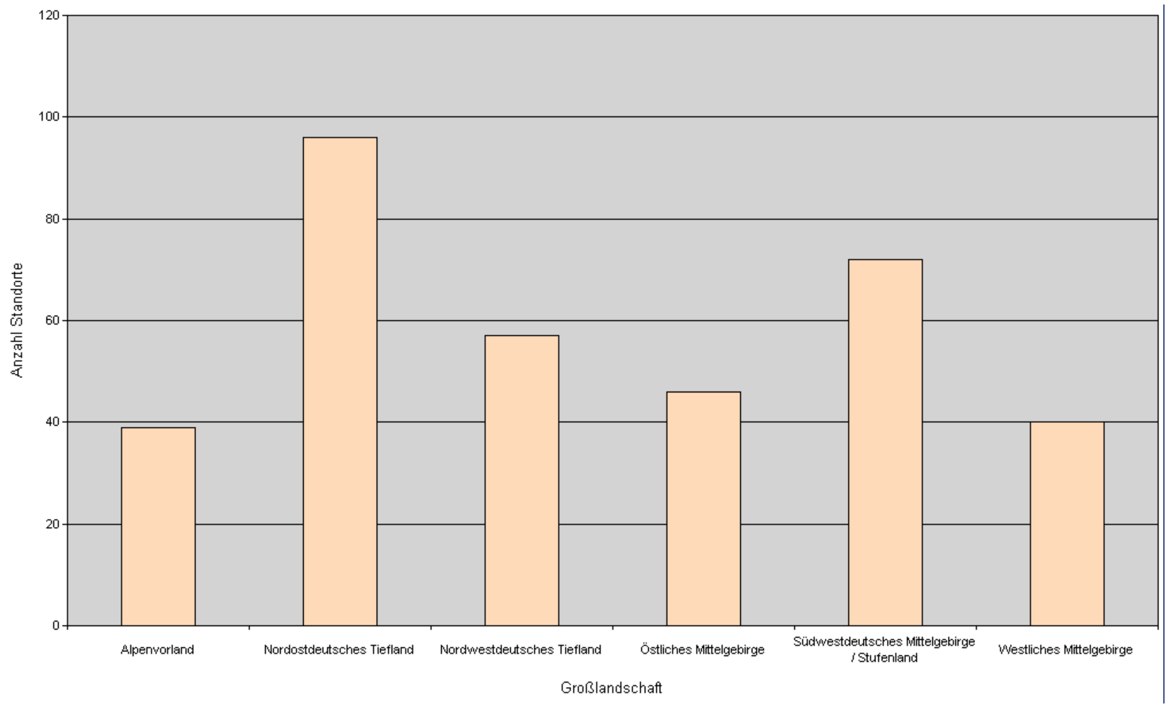

Abb. 12: Verteilung der BDF-Standorte hinsichtlich der naturräumlichen Großlandschaften (Gliederung des BfN auf Basis von MEYNEN et al. 1953-62 & IFAG 1979)

2.4 Verwendbarkeit von BDF als Referenz

Um eine nachhaltige Nutzung des BDF-Programmes für das Monitoring von GVO zu gewährleisten sollte die Frage beantwortet werden, wie stark bestehende BDF in der Vergangenheit bereits gegenüber GVO exponiert waren bzw. in welchem Ausmaß eine Exposition bei einem zukünftigen Anbau zu erwarten ist. Als Beispiel-GVO wurde in Absprache mit dem Auftraggeber die insektenresistente Maissorte MON810 als bislang einzige in Deutschland auf größerer Fläche angebaute GVO ausgewählt bei angenommenen Ausbreitungsradien (Expositionsdistanzen) für Pollen von 50, 150 und 1000 m. Diese Radien wurden in Anlehnung an unterschiedliche Abstandsregelungen in der EU zu konventionellen Anbauflächen (z.B. Spanien: 50 m, Deutschland: 150 m; GENTPFLEV 2008) sowie Empfehlungen aus Feldversuchen zur Maispollenausbreitung ausgewählt. So empfehlen beispielsweise FELKE & LANGENBRUCH (2005) einen Abstand von 1000 m zu Naturschutzgebieten und laut HOFMANN et al. (2008b) ist ein solcher Abstand notwendig, um mit einer 90-prozentigen Wahrscheinlichkeit Depositionen über 100.000 Maispollen/m² auszuschließen. Die folgenden Expositionsszenarien sind prinzipiell denkbar: 1) auf der BDF wurde der GVO angebaut; 2) die BDF lag nicht im Einflussbereich des GVO; 3) kein Anbau des GVO auf der BDF aber BDF lag im direkten Einflussbereich des GVO basierend auf den drei Pollen-Ausbreitungsradien. Diese Szenarien konnten im vorliegenden Projekt nicht deutschlandweit untersucht werden, sondern wurden beispielhaft für einzelne Bundesländer bearbeitet. Hierfür wurden in Absprache mit dem Auftraggeber vier Bundesländer ausgewählt: Brandenburg, Hessen, Niedersachsen sowie Schleswig-Holstein.

Die Gesamtanbaufläche von MON810 in Deutschland erreichte im Jahre 2008 mit ca. 3200 ha sein Maximum, bevor 2009 das Anbauverbot für MON810 aufgrund der Ruhensanordnung der Anbaugenehmigung nach §20 Abs. 2 GenTG in Kraft trat. Wie Abb. 13 zeigt (man beachte die logarithmische Skalierung der Y-Achse) konzentrierte sich der MON810-Anbau vor allem auf vier östliche Bundesländer (Brandenburg, Mecklenburg-Vorpommern, Sachsen und Sachsen-Anhalt), wobei der stärkste Anbau im Jahre 2007 in Brandenburg erfolgte (ca. 1350 ha). Die drei anderen ausgewählten Bundesländer hatten mit maximal ca. 0,25 ha (Hessen 2007), ca. 18 ha (Niedersachsen 2007) sowie ca. 0,04 ha (Schleswig-Holstein 2008) einen weitaus geringeren MON810-Anbau.

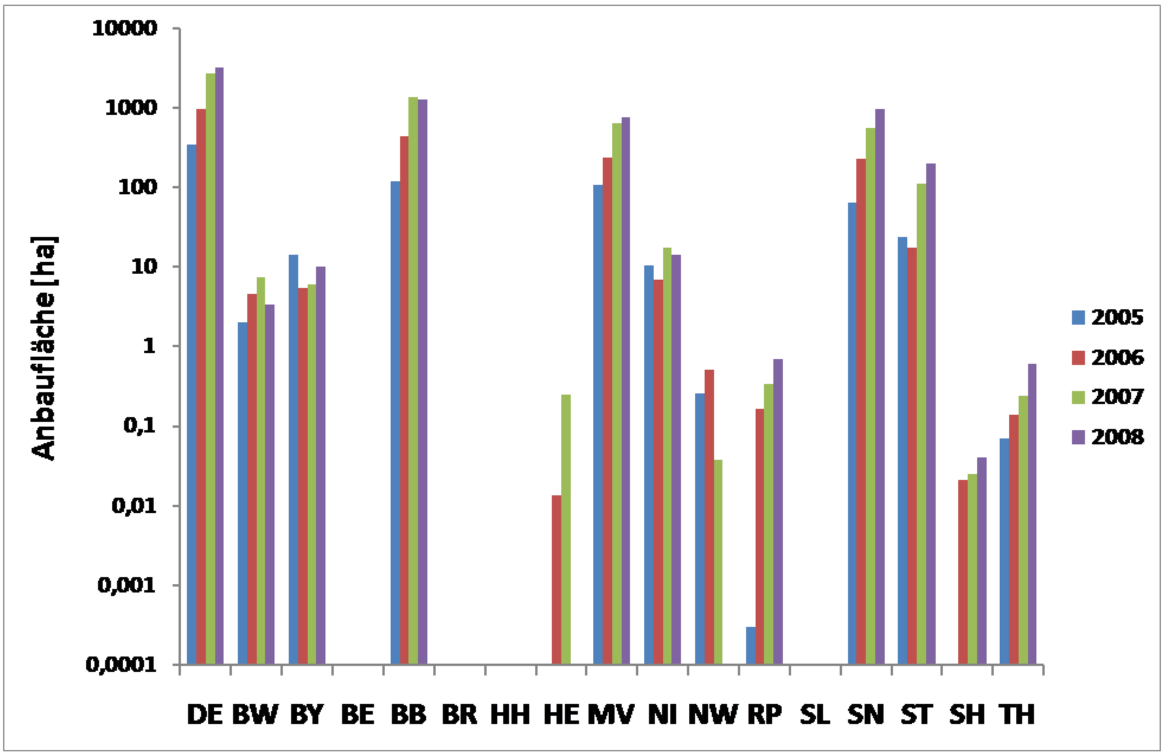

Abb. 13: Fläche des MON810-Anbaus in den deutschen Bundesländern 2005-2008.

2.4.1 **Methodik**

Für die vier ausgewählten Bundesländer wurden zunächst die jeweiligen InVeKoS (Integriertes Verwaltungs- und Kontrollsystem) Daten direkt oder durch Vermittlung von jeweils zuständigen Landesbehörden erhalten, mit denen landwirtschaftlich genutzte Parzellen GIS-basiert identifiziert werden können. Zudem wurden von Vertretern dieser Bundesländer die Koordinaten der Acker-BDF zur Verfügung gestellt. Aus Gründen des Datenschutzes wurden nicht von allen Bundesländern, sondern nur von Hessen, punktgenaue Koordinaten geliefert (Tab. 3). Das Land Niedersachsen lieferte zunächst abgerundete Hoch- und Rechtswerte, d.h. von den Koordinaten erstreckte sich nach Nord und Ost ein Suchraum von 1x1 km. Würde eine GVO-Fläche in diesen Suchraum hineinreichen, würden für diese Fläche genauere Koordinaten bereitgestellt, wie es auch bei Planungen im Lande üblich ist. Von den Ländern Brandenburg und Schleswig-Holstein wurden die zu den BDF gehörigen Feldblocknummern angegeben.

Zur Ermittlung der Standorte mit MON810-Anbau in den genannten Bundesländern wurden zunächst die öffentlich verfügbaren Angaben aus dem Standortregister (www.standortregister.de) in eine MS Access-Datenbank übernommen. Die Anbauflächen sind anhand der Angaben zu Postleitzahl, Gemeinde, Gemarkung, Flur, Flurstück und Schlagnummer bzw. –name identifizierbar. Unter Benutzung von ebenfalls öffentlich verfügbaren GeoWebDiensten der Bundesländer wurden die Koordinaten der MON810-Anbauflächen in unterschiedlicher räumlicher Auflösung abgeschätzt, jedoch mindestens auf der Ebene der Flurmittelpunkte (Tab. 3). Diese Koordinaten wurden anschließend im Programm Quantum GIS (Version 1.7.4) mit den InVeKoS-Daten verschnitten, um eine Zuordnung der jeweiligen Anbaufläche zu einem bestimmten Feldblock zu erreichen. Das

Ergebnis ist dabei mit einer Restunsicherheit behaftet, das heißt es wird nicht der Anspruch erhoben, zu jeder MON810-Anbaufläche zweifelsfrei den dazu gehörigen Feldblock identifiziert zu haben. Das erhaltene Resultat repräsentiert jedoch die mit den verfügbaren Daten bestmögliche Annäherung an die realen Verhältnisse und wird für die Zwecke der hier erfolgten Abschätzung als ausreichend genau betrachtet.

Tab. 3: Datenherkunft, -genauigkeit und –abfrage zur Darstellung im GIS-Programm.

	**Brandenburg**	**Hessen**	**Niedersachsen**	**Schleswig-Holstein**
**Zahl Acker-BDF**	20	26	46	17
**Genauigkeit der BDF-Koordinaten**	Feldblock-ID	punktgenau	1x1 km	Feldblock-ID
**Zahl MON810-Meldungen 2005-2008**	152	5	28	5
**Koordinaten GVO-Anbauflächen**	Quelle: http://isk.geobasis-bb.de/index.php/bb-viewer<br>Genauigkeit: flurgenau<br>Abfragedatum: 29.02.2012	Quelle: http://hessenviewer.hessen.de<br>Genauigkeit: flurstückgenau<br>Abfragedatum: 14.03.2012	Quelle: http://www.landmap-niedersachsen.de<br>Genauigkeit: flurstückgenau<br>Abfragedatum: 11.06.2012	Quelle: http://141.91.150.41/feldblockfinder<br>Genauigkeit: flurstückgenau<br>Abfragedatum: 15.03.2012

Für die Einschätzung, inwieweit die BDF gegenüber GVO exponiert waren, wurden die BDF-Standorte und MON810-Anbauflächen in einer gemeinsamen Karte dargestellt und die oben genannten drei Pollen-Ausbreitungsradien angenommen, die im GIS als Pufferabstände um die den MON810-Anbauflächen zugeordneten Feldblöcke gelegt wurden: 50, 150, 1000 m.

2.4.2 Ergebnisse

In Abb. 14 findet sich eine Übersicht über die Lage der Acker-BDF sowie der bisherigen Anbaustandorte von MON810 in den vier betrachteten Beispiel-Bundesländern.

Brandenburg

Der bislang intensivste Anbau von MON810 in Deutschland fand im Brandenburgischen Landkreis Oberhavel statt (2007 ca. 1,3%; 2008 ca. 0,6% der Ackerfläche; Abb. 15), gefolgt von den Landkreisen Märkisch-Oberland (2008 ca. 0,5%) und Spree-Neiße (2008 ca. 0,4%). In diesen drei Landkreisen sowie zusätzlich im Landkreis Uckermark ist es in der Vergangenheit möglicher Weise bereits zur Exposition von Acker-BDF gegenüber MON810 gekommen (Abb. 16), sodass diese im Folgenden näher betrachtet werden.

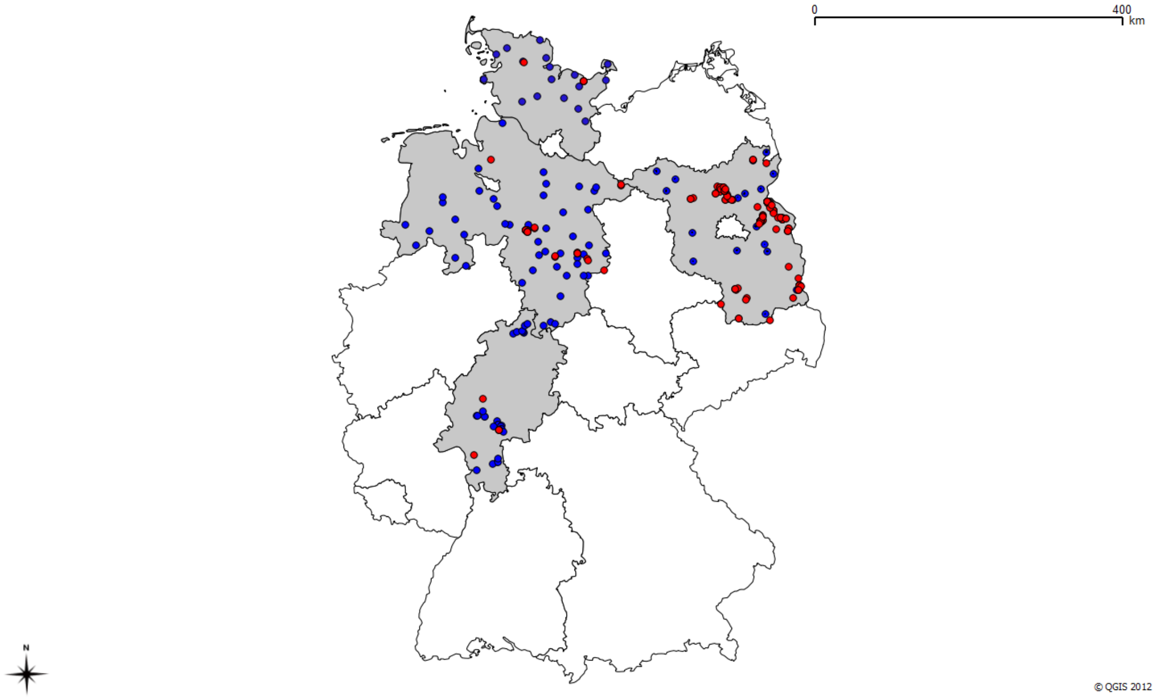

Abb. 14: Beispiel Bundesländer (BL), für die InVeKoS Datensätze angefragt wurden. Blaue Punkte: Lage der Acker-BDF. Rote Punkte: Lage der MON810-Anbauflächen 2005-2008.

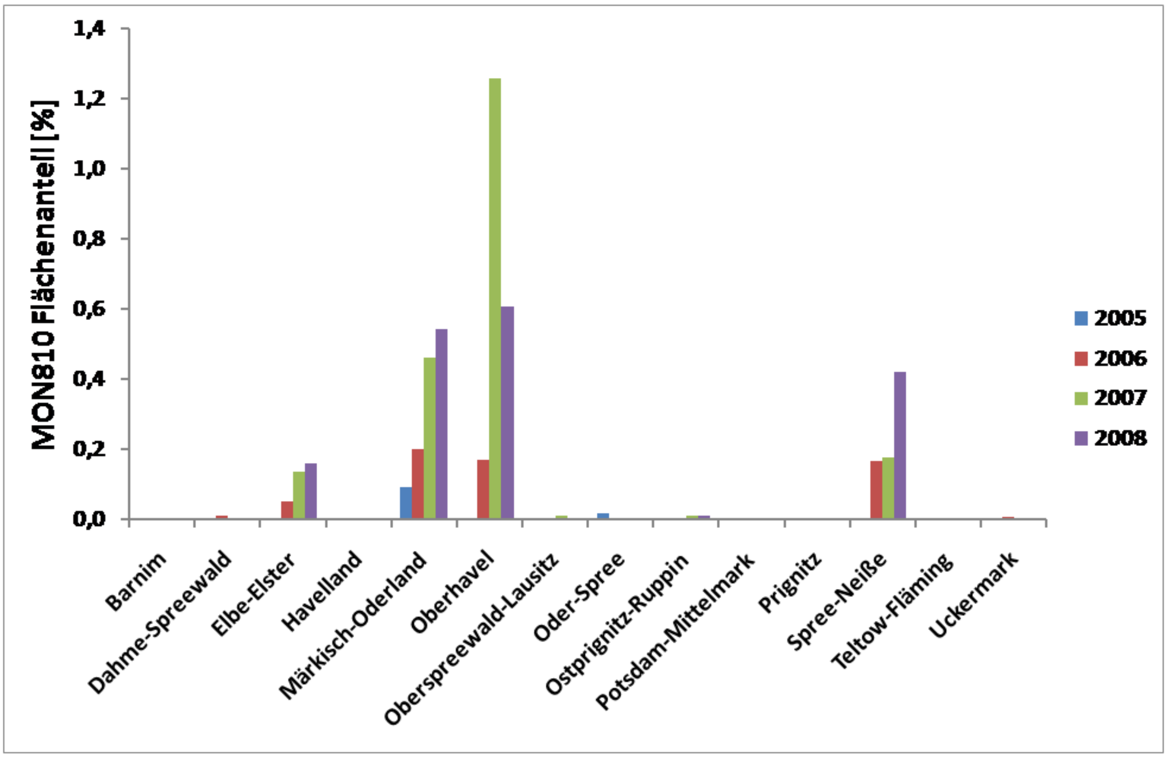

Abb. 15: Anteil des MON810-Anbaus in Brandenburg an der gesamt Ackerfläche 2005-2008.

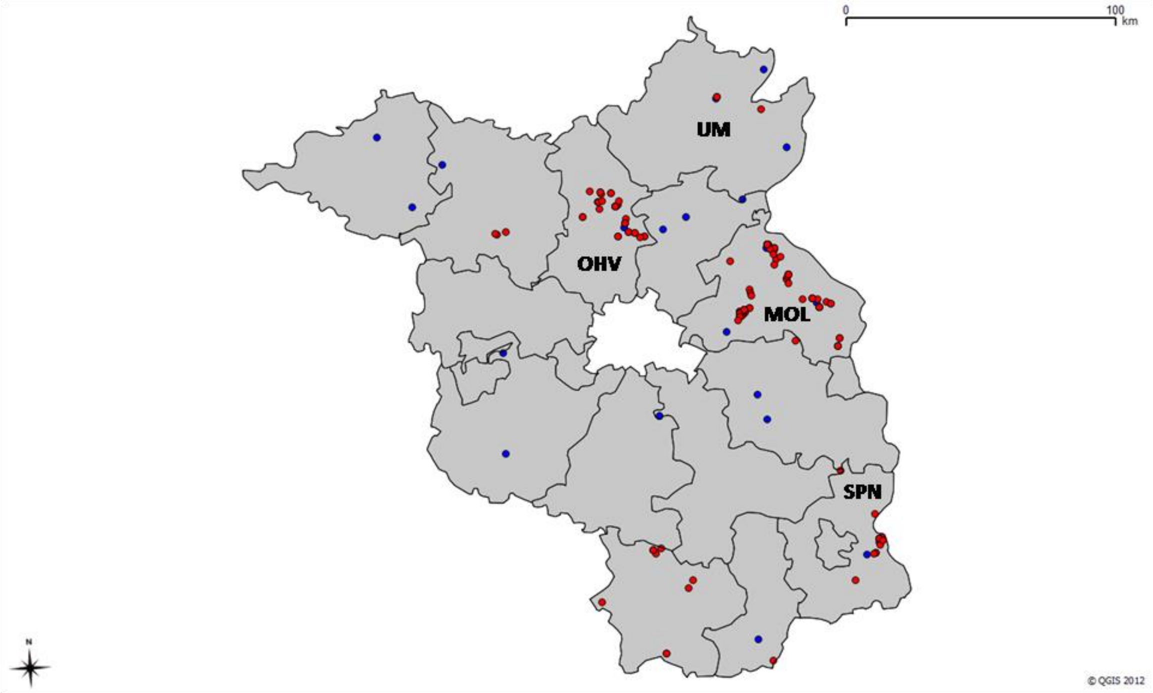

Abb.16: Lage der Acker-BDF (blaue Punkte) und MON810-Anbau in Brandenburg 2005-2008 (rote Punkte). Die Landkreise Märkisch-Oberland (MOL), Oberhavel (OHV), Spree-Neiße (SPN) und Uckermark (UM) wurden aufgrund möglicher MON810-Exposition von BDF näher betrachte

Im Landkreis Märkisch-Oderland liegen die Feldblöcke von zwei der drei Acker-BDF in unmittelbarer Nähe zu ehemaligen MON810-Anbauflächen (Abb. 17). Der Feldblock der BDF „Gusow" befindet sich zu einem Großteil im Überlappungsbereich bei einer angenommenen Pollenausbreitung von 1000 m von einer ehemaligen MON810-Anbaufläche und grenzt unmittelbar an diese Fläche, sodass hier von einer Exposition der BDF gegenüber MON810-Pollen in der Vergangenheit ausgegangen werden kann (Abb. 18). Gleiches gilt für die BDF „Rathsdorf", deren Feldblock sich innerhalb des 1000 m Radius um mehrere ehemalige MON810-Anbauflächen befindet und direkt an zwei dieser Flächen grenzt (Abb. 19).

Im Landkreis Oberhavel liegt der Feldblock der einzigen Acker-BDF „Neuholland" vollständig in Überlappung mit einem angenommenen Pollenausbreitungsradius von 1000 m um ein bis zwei ehemalige MON810-Anbauflächen, grenzt jedoch nicht unmittelbar an diese Flächen. Dennoch kann hier von einer Exposition der BDF gegenüber MON810-Pollen in der Vergangenheit ausgegangen werden (Abb. 20 und Abb. 21).

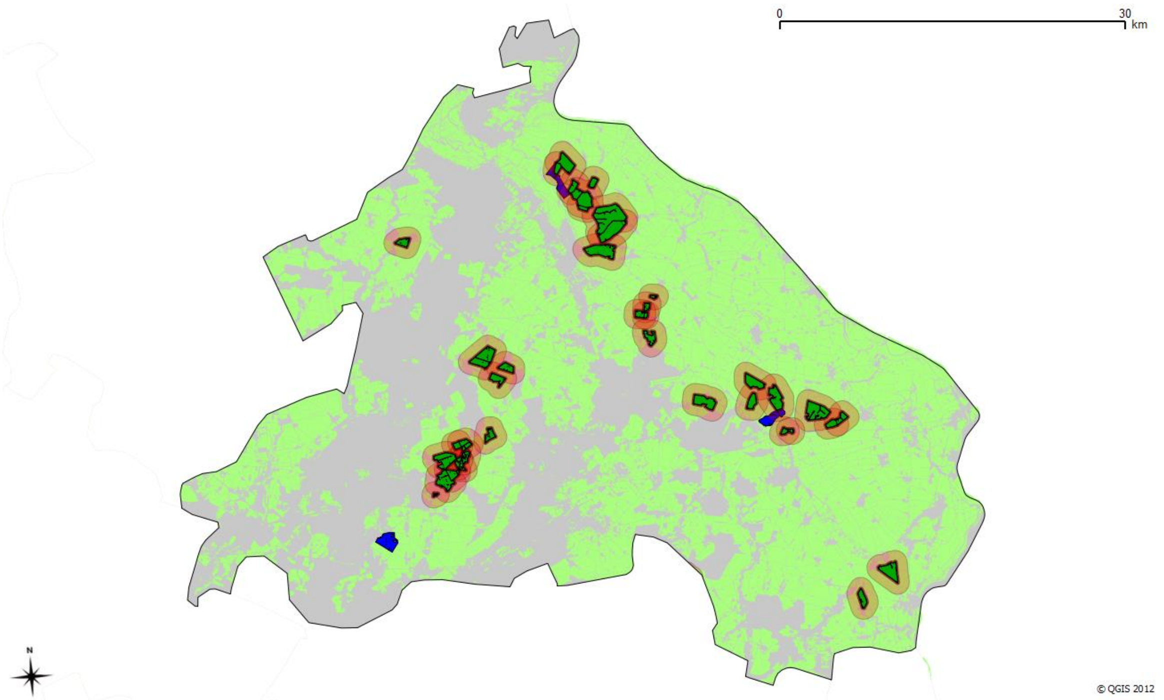

Abb. 17: Feldblöcke mit Acker-BDF (blau) und mit MON810-Anbau (dunkelgrün) unter Annahme einer Pollenausbreitung von 50, 100 und 1000 m (rote Puffer) im Landkreis Märkisch-Oderland (Brandenburg) 2005-2008.

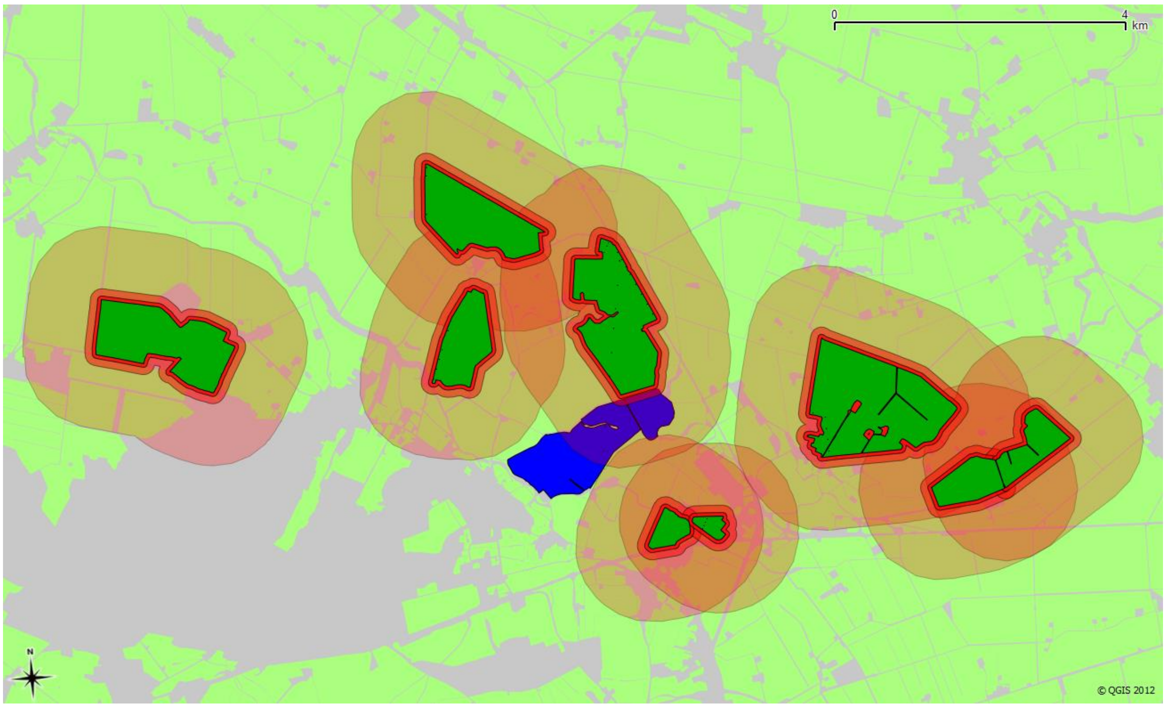

Abb. 18: Exposition des Feldblocks der Acker-BDF Gusow (blau) im Landkreis Märkisch-Oderland (Brandenburg) gegenüber MON810-Anbau (dunkelgrün) unter Annahme einer Pollenausbreitung von 50, 100 und 1000 m (rote Puffer) 2005-2008.

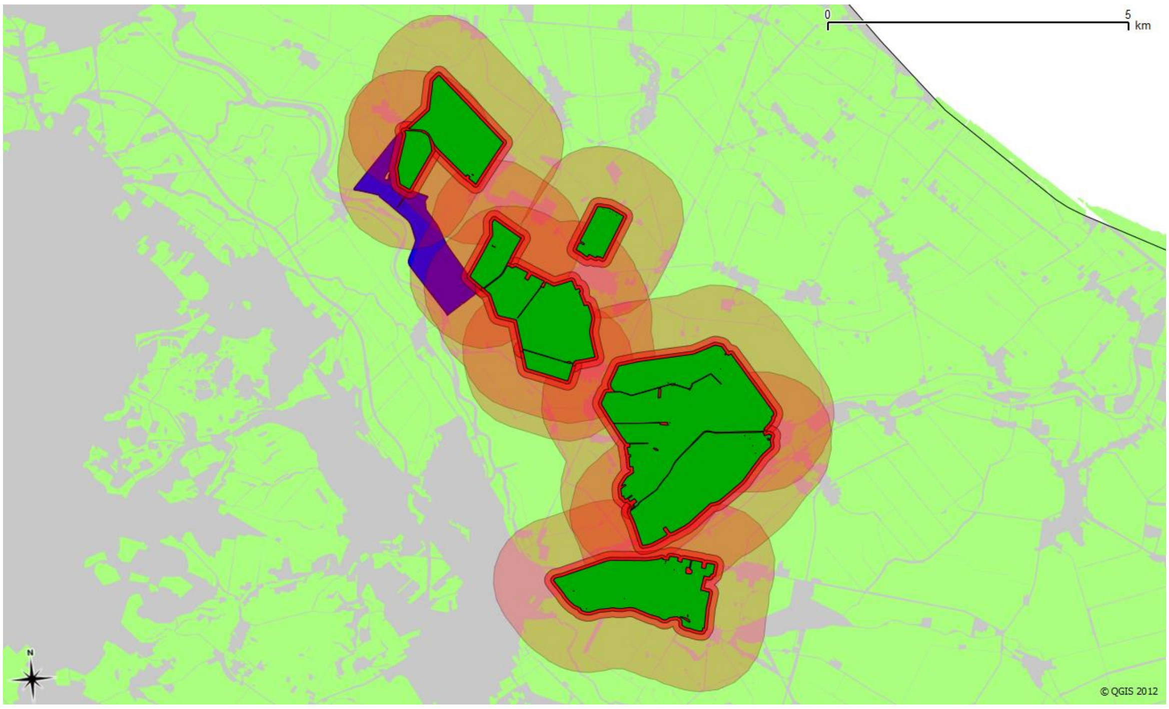

Abb. 19: Exposition des Feldblocks der Acker-BDF Rathsdorf (blau) im Landkreis Märkisch-Oderland (Brandenburg) gegenüber MON810-Anbau (dunkelgrün) unter Annahme einer Pollenausbreitung von 50, 100 und 1000 m (rote Puffer) 2005-2008.

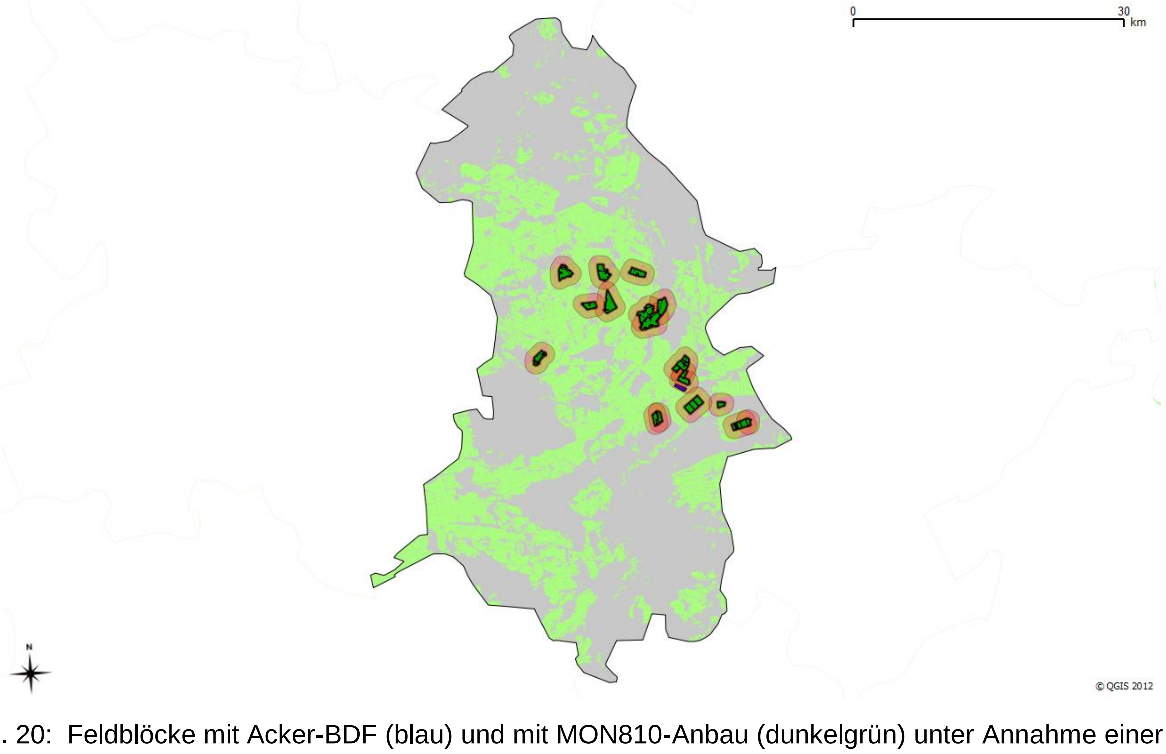

Abb. 20: Feldblöcke mit Acker-BDF (blau) und mit MON810-Anbau (dunkelgrün) unter Annahme einer Pollenausbreitung von 50, 100 und 1000 m (rote Puffer) im Landkreis Oberhavel (Brandenburg) 2005-2008.

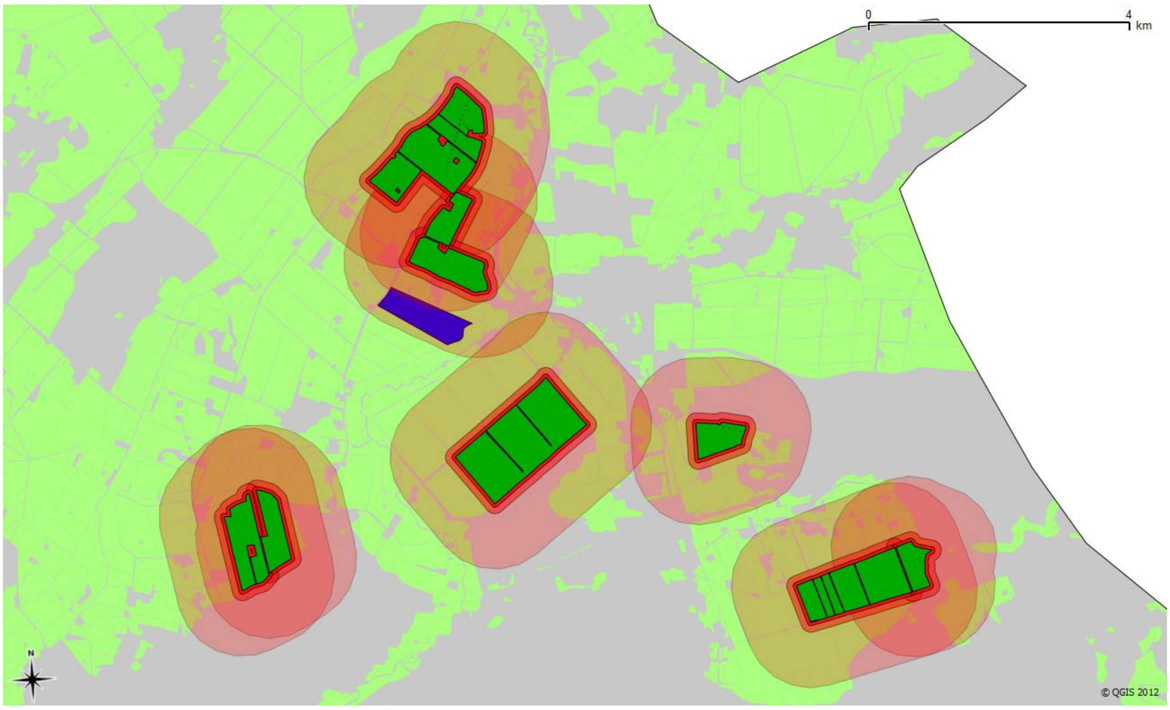

Abb. 21 Exposition des Feldblocks der Acker-BDF Neuholland (blau) im Landkreis Oberhavel (Brandenburg) gegenüber MON810-Anbau (dunkelgrün) unter Annahme einer Pollenausbreitung von 50, 100 und 1000 m (rote Puffer) 2005-2008.

Im Landkreis Spree-Neiße befindet sich ebenfalls eine Acker-BDF, die sich jedoch nicht innerhalb eines 1000 m Radius um eine der ehemaligen MON810-Anbauflächen befindet und somit nach diesem Szenario bislang keine Exposition gegenüber MON810-Pollen erfolgt ist (Abb. 22).

Im Landkreis Uckermark befinden sich insgesamt vier Acker-BDF (Abb. 23). Der Feldblock der Acker-BDF „Augustenfelde“ befindet sich vollständig in Überlappung mit einem angenommenen Pollenausbreitungsradius von 1000 m um eine ehemalige MON810-Anbaufläche, grenzt aber nicht direkt an diese Fläche. Dennoch kann hier ebenfalls von einer Exposition der BDF gegenüber MON810-Pollen in der Vergangenheit ausgegangen werden (Abb. 24).

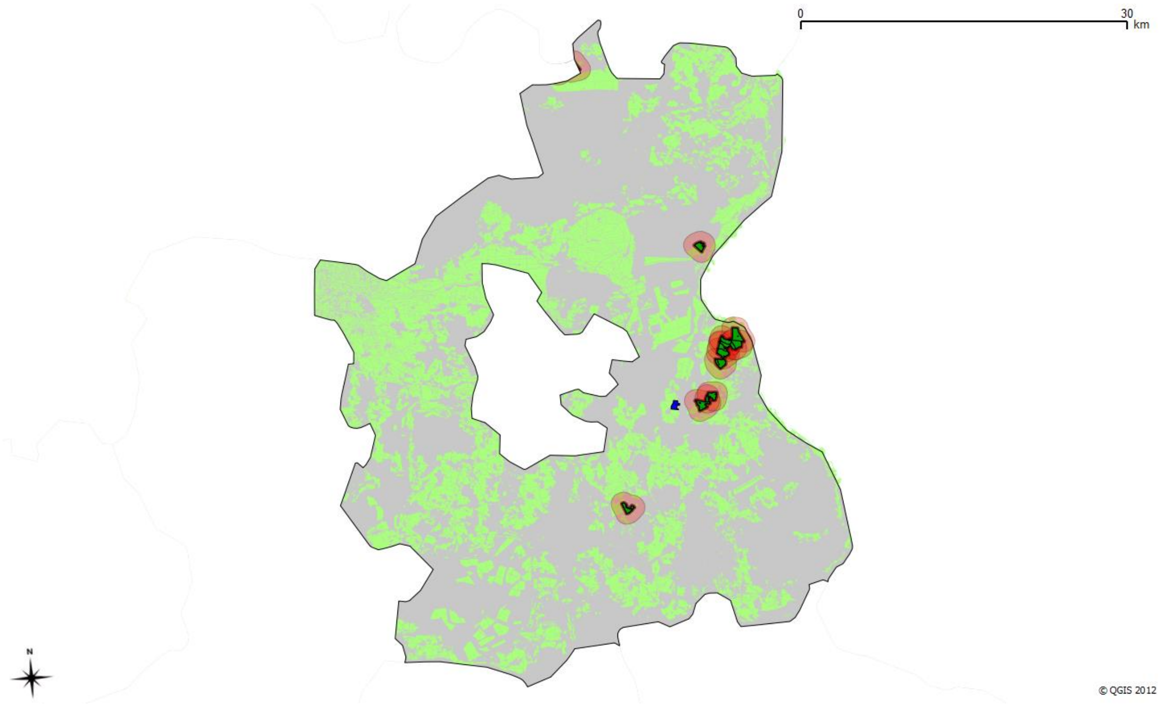

Abb. 22: Feldblöcke mit Acker-BDF (blau) und mit MON810-Anbau (dunkelgrün) unter Annahme einer Pollenausbreitung von 50, 100 und 1000 m (rote Puffer) im Landkreis Spree-Neiße (Brandenburg) 2005-2008.

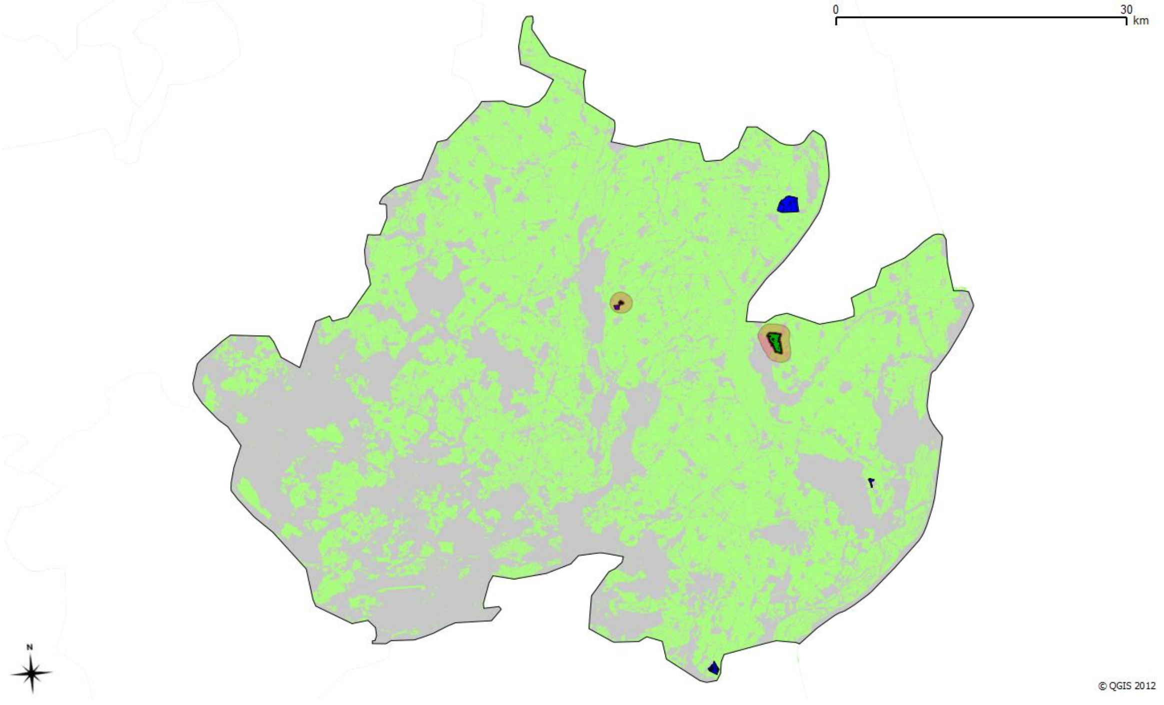

Abb. 23: Feldblöcke mit Acker-BDF (blau) und mit MON810-Anbau (dunkelgrün) unter Annahme einer Pollenausbreitung von 50, 100 und 1000 m (rote Puffer) im Landkreis Uckermark (Brandenburg) 2005-2008.

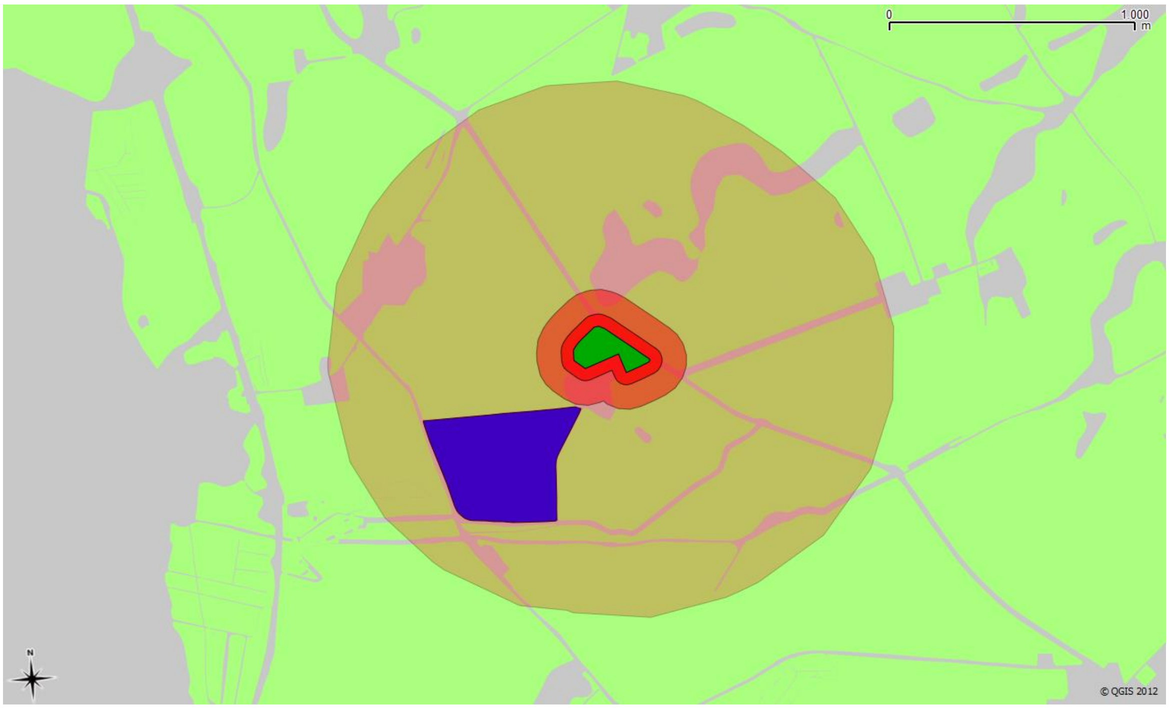

Abb. 24: Exposition des Feldblocks der Acker-BDF Augustenfelde (blau) im Landkreis Uckermark (Brandenburg) gegenüber MON810-Anbau (dunkelgrün) unter Annahme einer Pollenausbreitung von 50, 100 und 1000 m (rote Puffer) 2005-2008.

Hessen

In Hessen fand bislang nur auf drei verschiedenen Feldblöcken ein Anbau von MON810 statt (Abb. 25). Eine dieser Flächen im Main-Kinzig-Kreis befindet sich in der Nähe zu einer der drei Acker-BDF, es kommt jedoch zu keiner Überlappung mit dem 1000 m Radius um die ehemalige MON810-Anbaufläche (Abb. 26). Somit hat gemäß diesem Szenario bislang keine Exposition von Acker-BDF gegenüber MON810-Pollen in Hessen stattgefunden.

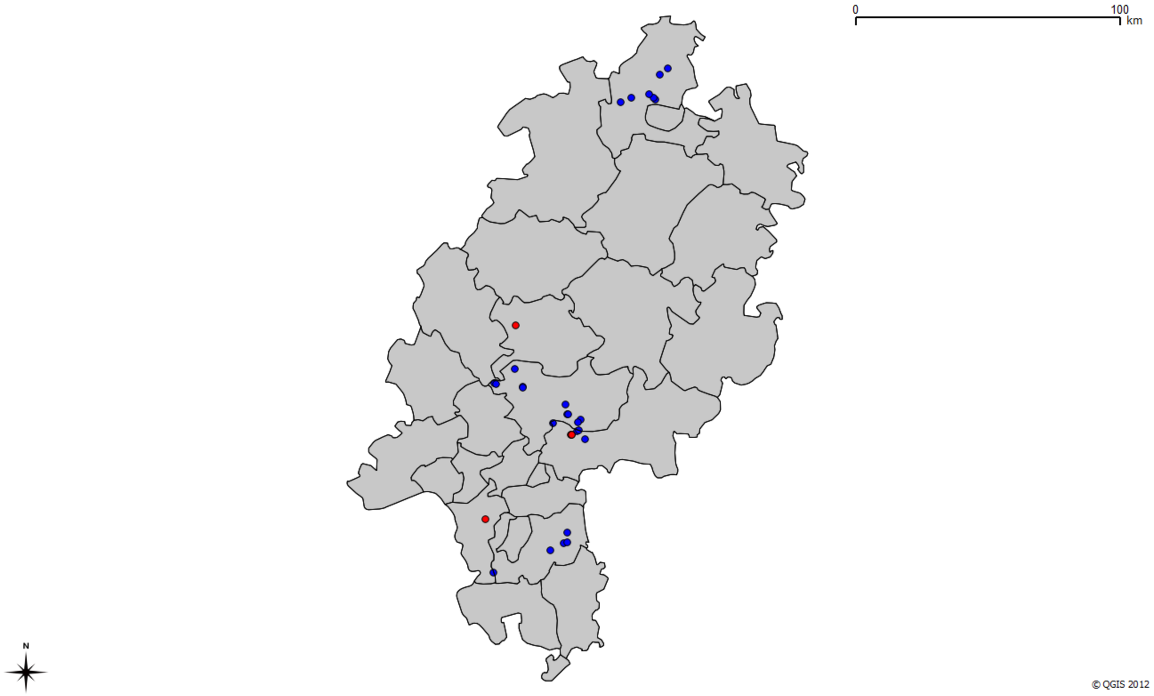

Abb. 25: Lage der Acker-BDF (blaue Punkte) und MON810-Anbau in Hessen 2005-2008 (rote Punkte).

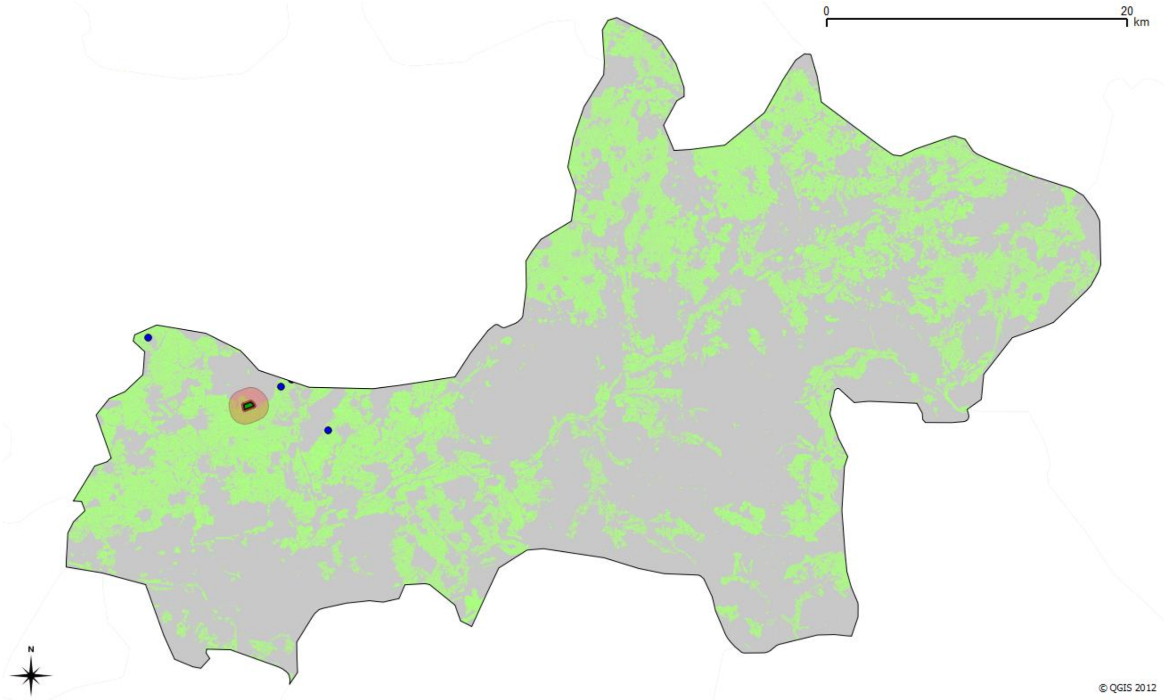

Abb. 26: Feldblöcke mit Acker-BDF (blau) und mit MON810-Anbau (dunkelgrün) unter Annahme einer Pollenausbreitung von 50, 100 und 1000 m (rote Puffer) im Landkreis Main-Kinzig-Kreis (Hessen) 2005-2008.

Niedersachsen

Die 46 im Bundesland Niedersachsen befindlichen Acker-BDF befinden sich ausnahmslos außerhalb des 1000 m Ausbreitungsradius der Pollen ehemaliger MON810-Anbauflächen (Abb. 27). Daher ist gemäß diesem Szenario bislang von keiner Exposition von Acker-BDF gegenüber MON810-Pollen in Niedersachsen auszugehen.

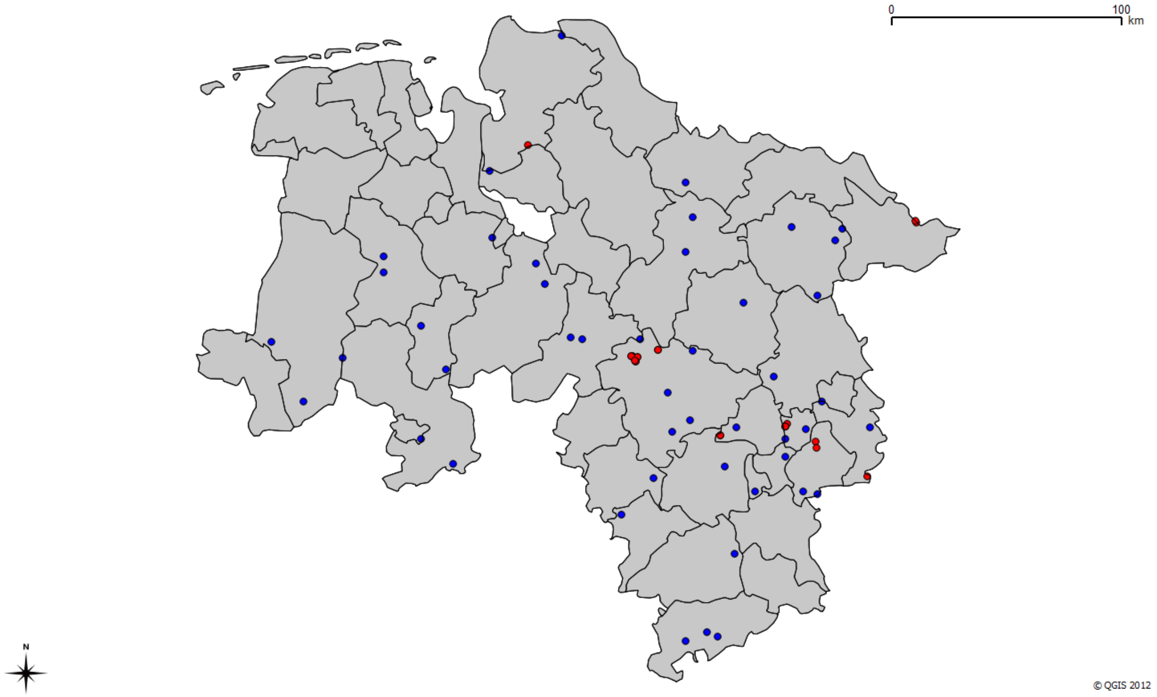

Abb. 27: Lage der Acker-BDF (blaue Punkte) und MON810-Anbau in Niedersachsen 2005-2008 (rote Punkte).

Schleswig-Holstein

In Schleswig-Holstein fand bislang wie in Hessen nur auf drei verschiedenen Feldblöcken ein Anbau von MON810 statt (Abb. 28). Eine dieser Flächen im Landkreis Schleswig-Flensburg befindet sich in der Nähe des Feldblocks von einer der zwei Acker-BDF (Abb. 29). Es kommt jedoch nur zu einer minimalen Überlappung mit dem 1000 m Radius um die ehemalige MON810-Anbaufläche (Abb. 30), sodass es eher unwahrscheinlich erscheint, dass die im Innern des Feldblocks befindliche eigentliche BDF-Fläche hiervon betroffen ist. Somit hat gemäß diesem Szenario in Schleswig-Holstein ebenfalls bislang keine Exposition von Acker-BDF gegenüber MON810-Pollen stattgefunden.

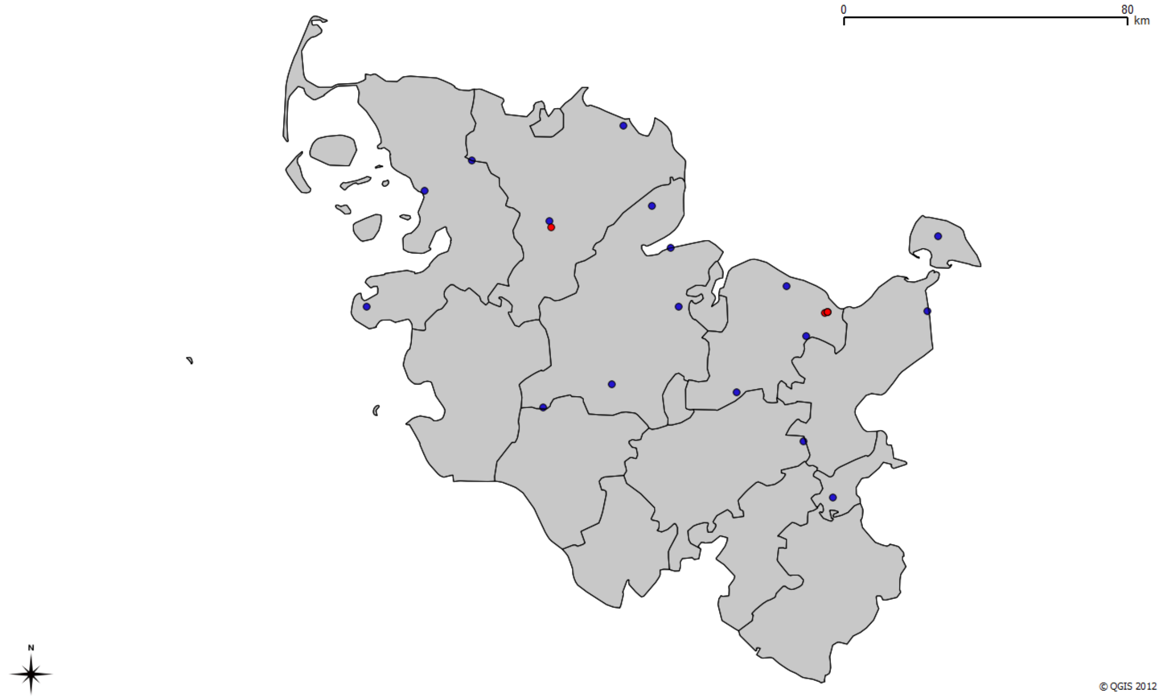

Abb. 28: Lage der Acker-BDF (blaue Punkte) und MON810-Anbau in Schleswig-Holstein 2005-2008 (rote Punkte).

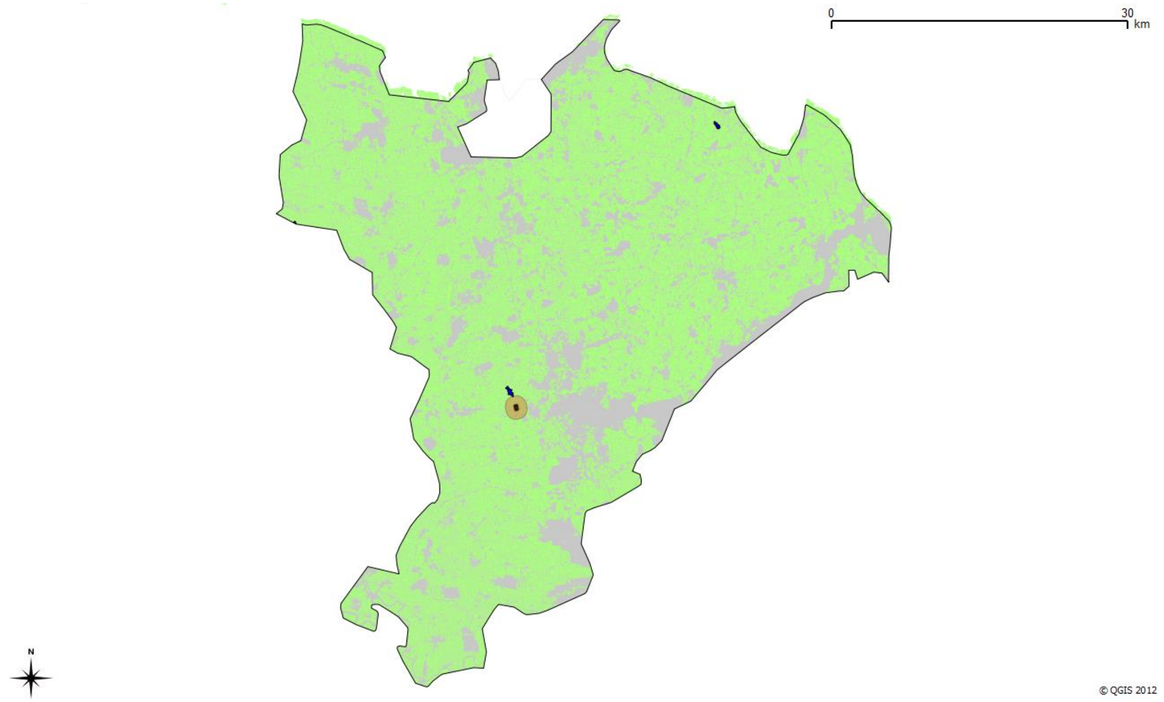

Abb. 29: Feldblöcke mit Acker-BDF (blau) und mit MON810-Anbau (dunkelgrün) unter Annahme einer Pollenausbreitung von 50, 100 und 1000 m (rote Puffer) im Landkreis Schleswig-Flensburg (Schleswig-Holstein) 2005-2008

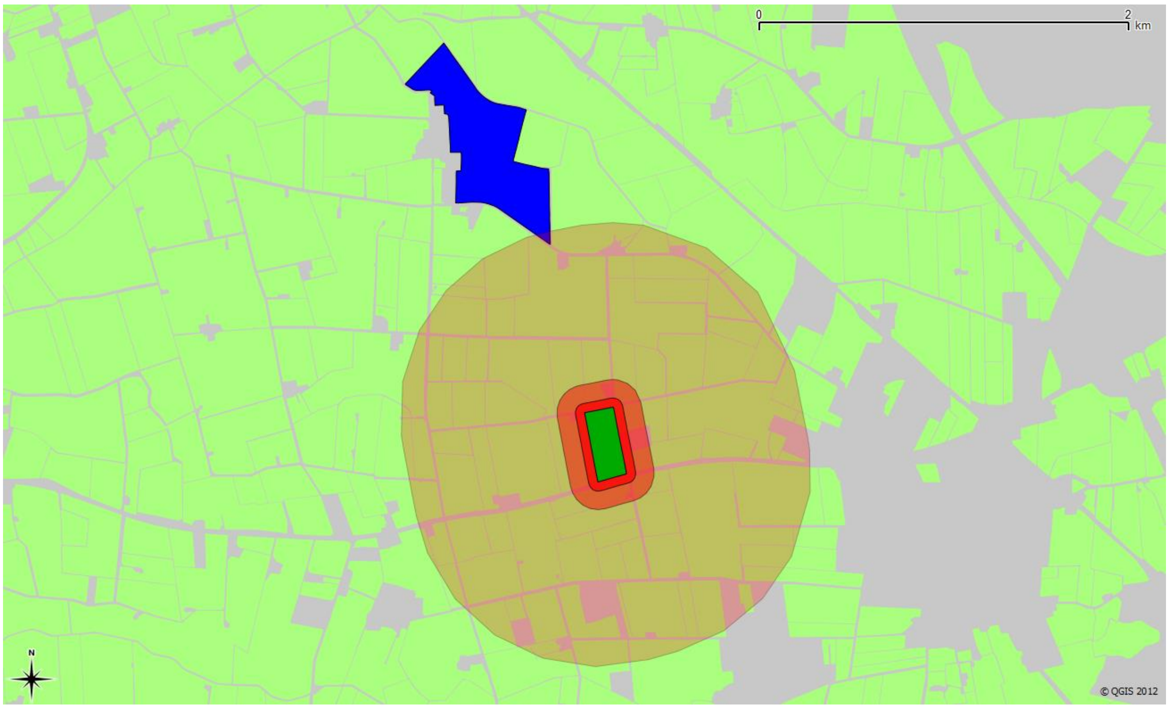

Abb. 30: Exposition des Feldblocks der Acker-BDF Schuby (blau) im Landkreis Schleswig-Flensburg (Schleswig-Holstein) gegenüber MON810-Anbau (dunkelgrün) unter Annahme einer Pollenausbreitung von 50, 100 und 1000 m (rote Puffer) 2005-2008.

2.4.3 Fazit

Von den insgesamt 109 Acker-BDF in den betrachteten vier Beispielbundesländern waren in der Zeit von 2005-2008 mit hoher Wahrscheinlichkeit bereits vier gegenüber MON810-Pollen exponiert, was einem Anteil von 3,7% entspricht. Alle dieser BDF liegen in Brandenburg, wo der bislang intensivste MON810-Anbau stattgefunden hat. Gemessen an den landesweit 20 Acker-BDF beträgt der Anteil der exponierten BDF somit bereits 20%. Ein Anbau von MON810 direkt auf einer BDF hat hingegen offenbar bislang nicht stattgefunden. Bei einer möglicherweise künftigen Intensivierung des GVO-Anbaus in Deutschland ist davon auszugehen, dass weitere Acker-BDF früher oder später gegenüber GVO exponiert sein werden. Um künftig BDF als unbeeinflusste Flächen zum Monitoring von GVO nutzen zu können bedarf es daher Maßnahmen zum Schutz dieser Flächen (z.B. kein GVO-Anbau auf BDF, GVO-freie Pufferzonen) sowie für die bereits exponierten BDF eine Festlegung, welcher zeitliche Mindestabstand seit der letzten GVO-Exposition gegeben sein muss, um die jeweilige Fläche wieder für ein GVO-Monitoring nutzen zu können. Diese räumlichen und zeitlichen Abstände müssen so weit wie möglich auf wissenschaftlicher Grundlage festgelegt werden. Hierbei kann es fallbezogen unterschiedliche Anforderungen geben (z.B. unterschiedliche Pollenflugradien für verschiedene Kulturarten) für die ein gemeinsames Vorgehen gefunden werden muss. Es muss jedoch auch beachtet werden, dass ab einer gewissen GVO-Anbauintensität und –dauer langfristig kaum noch von einer völligen Isolation der BDF und anderer zu schützender Flächen (konventioneller oder ökologischer Landbau, Naturschutzgebiete, etc.) von GVO-Produkten ausgegangen werden kann (siehe z.B. HOFMANN et al. 2008b oder der diffuse Eintrag von Schwermetallen und Organika über den Luft-Eintragspfad in

praktisch alle Böden Deutschlands). Daher ist die Festlegung akzeptabler Abstandsregelungen nicht zuletzt auch eine sozio-ökonomische Fragestellung.

2.4.4 Darstellung der zoologischen Erhebungen auf BDF

Sehr fragmentarisch und ähnlich heterogen wie die Standortzahl ist die Abdeckung bodenbiologischer Daten auf den BDFs (Tab. 4). Vegetationskundliche Erhebungen (Arten, Deckungsgrade etc.) werden nur in Schleswig-Holstein ausgewiesen, die Mikrobiologie (verschiedene Parameter; durchgängig nur die Bodenatmung bzw. die daraus abgeleitete Biomasse) immerhin in acht Ländern. Fünf Bundesländer haben in der Vergangenheit bodenzoologische Daten erfasst: Brandenburg, Schleswig-Holstein sowie mit Abstrichen Nordrhein-Westfalen, und, kursorisch, Hamburg und Thüringen (Daten aus Bayern standen bei Abschluss dieses Vorhabens noch nicht zur Verfügung). Dabei handelt es sich fast ausschließlich um die Beprobung von Regenwürmern und Enchytraeen, obwohl in den entsprechenden Empfehlungen (BARTH et al. 2000) durchaus auch andere Gruppen genannt wurden. Zudem wurden die Daten nicht immer mit den gleichen Methoden erhoben. In Abb. 31 sind die Datensätze mit Lumbriciden und Enchytraeen, getrennt nach Biotoptyp, dargestellt. So fanden die umfangreichsten Erhebungen auf Acker- und Laubwaldstandorten statt. In einem geringeren Umfang wurde Grünland trockener und frischer sowie feuchter Standorte berücksichtigt. Allerdings ist die real vorliegende Zahl in Deutschland vorliegender bodenbiologischer Daten durch Einbeziehung von Beprobungen außerhalb von BDFs deutlich höher, wie in dem oben erwähnten UBA-Vorhaben festgestellt wurde.

Tab. 4: Tabellarische Darstellung der zur Verfügung stehenden Datensätze zur Zoologie, Mikrobiologie und Vegetation.

	**Summe Standorte**	**Summe Datensätze**		
		**Zoologie**	**Mikrobiologie**	**Vegetation**
**BDF UBA-Datenbank (∑)**	**797**	**5797**	**3420**	**2478**
Baden-Württemberg	33	-	-	-
Bayern	289	-	124	-
Berlin	-	-	-	-
Brandenburg	32 (33)	3241	178	-
Bremen	-	-	-	-
Hamburg	3	137	-	-
Hessen	68	-	-	-
Mecklenburg-Vorpommern	46	-	-	-
Niedersachsen	90	-	635	-
Nordrhein-Westfalen	21	743	60	-
Rheinland-Pfalz	-	-	-	-
Saarland	11	-	-	-
Sachsen	55	-	-	-
Sachsen-Anhalt	78	-	727	-
Schleswig-Holstein	37 (38)	1599	1576	2478
Thüringen	32	77	120	-

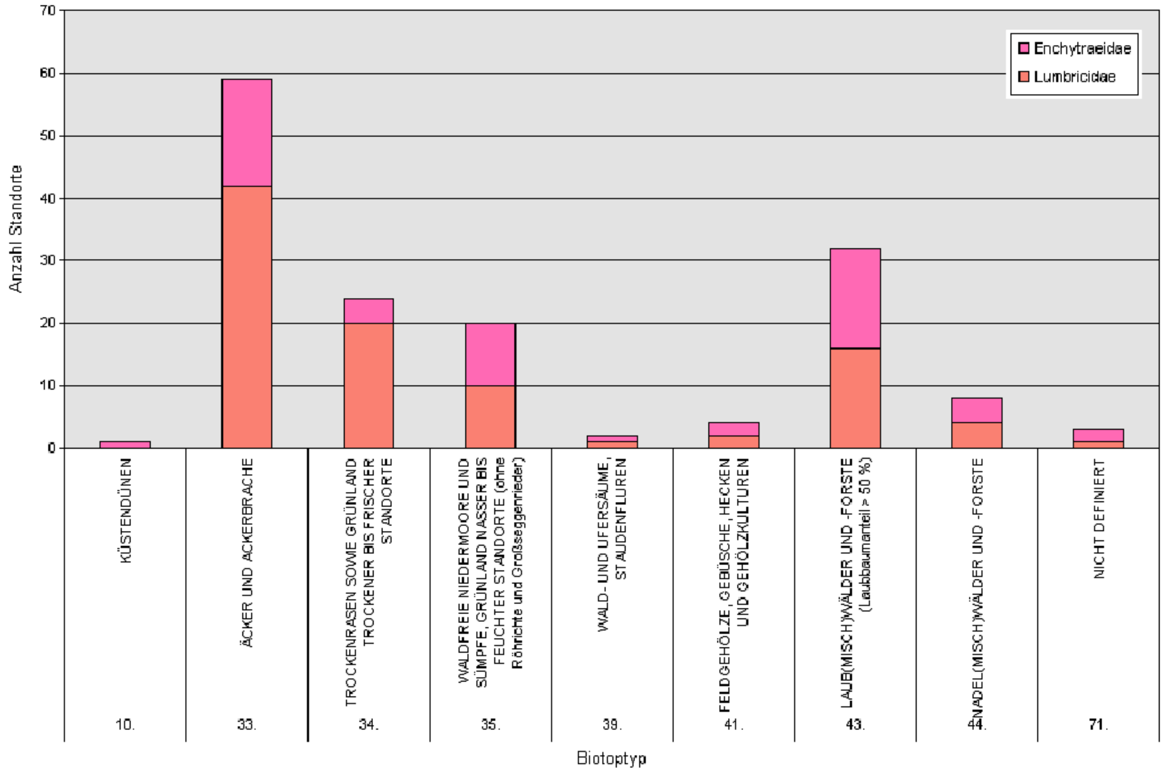

Abb. 31: Darstellung der Standorte mit zoologischen Erhebungen für Lumbriciden und Enchytraeen im Boden-Dauerbeobachtungsprogramm. Verteilung der Standorte auf die Basistypen der Biotoptypen Deutschlands.

2.5 Fazit: Repräsentativität der BDF, speziell in Hinsicht auf deren Nutzung für bodenbiologische Monitoringprogramme

Insgesamt kann damit nach derzeitigem Stand von einer guten Repräsentanz der BDF in Deutschland hinsichtlich Ackerstandorten (allgemein), Abbildung ökologischer Raumeinheiten bzw. Naturräumen ausgegangen werden. Dies bezieht sich jedoch lediglich auf den allgemein als Acker und Ackerbrachen bezogenen Basis-Biotoptyp. Für die Bodenorganismen sind aufgrund ihrer Lebensweise weitere standortspezifische Merkmale (Bodenparameter, Nährstoffversorgung, Feuchte etc.) für ihre Verteilung relevant, die sich in der tiefer gehenden Gliederung der Biotoptypen (2. und 3. Ebene) von Äckern und Ackerbrachen widerspiegeln. Zur tieferen Gliederung fehlen häufig genaue Angaben z.B. zum Untergrund (Kalk, Silikat, Sand etc.) bzw. zur allgemeinen Nährstoffversorgung (extensiv artenreich, intensiv, nährstoffreich, artenarm, etc.). Eine standardisierte und möglichst genaue Datenerhebung dieser Parameter ist anzustreben, da die Verteilung von Bodentieren auf der untersten Klassifikationsebene die stärksten Korrelationen zeigt (vgl. TOSCHKI 2008, ROß-NICKOLL et al. 2004, LENNARTZ 2003). Eine exakte Biotoptypenansprache der Standorte ist somit auch für statistische Belange erforderlich, um die im Freiland meist naturgegebenen hohen Varianzen für die Verteilung von Tieren möglichst gering zu halten.

Die BDF-Datensätze enthalten wenig bodenbiologische Daten, und der Datenbestand weist hinsichtlich der Vollständigkeit der Daten pro Tiergruppe folgende gravierende Lücken auf:

- Lumbricidae (bestes Datenpaket): 97 BDF-Standorte (von 737), repräsentativ für Grünland, Äcker und Wälder, aber Zuordnung zu tieferen Ebenen der Biotoptypenliste nur in Ansätzen möglich, in einigen Bundesländern lückenhaft;
- Enchytraeidae: Daten aus 60 BDF, muss repräsentativ ergänzt werden;
- Collembola, Oribatida, Carabidae, Araneae etc.: keine Datensätze aus BDF
- Mikroben: bisher keine Daten, die zur Beurteilung von Diversität geeignet sind.

Es gibt nicht genügend Standorte, an denen Daten mehrerer wichtiger Tiergruppen hinsichtlich Bodenbiodiversität und relevanter Bodenparameter zusammen vorliegen. Insgesamt betrachtet fehlen auch belastbare Angaben über die Biodiversität naturnaher Standorte. Methodisch fehlt außerdem ein zielführendes Beprobungskonzept (Mindeststandard), das in einem länderübergreifenden Ansatz realisiert werden müsste. Ein Abgleich mit den Ergebnissen der Arbeitsgruppe des VDI (RUF et al. 2013) ist in diesem Zusammenhang zu empfehlen. Darüber hinaus fehlen besonders Daten zur Beeinflussung von Bodenorganismen durch stoffliche Belastungen und andere Stressoren, z.B. Bodenverdichtung oder klimatische Faktoren, unter Freilandbedingungen, die zur Definition von Schwellenwerten genutzt werden können. Bodenphysikalische Daten liegen für die BDF in umfangreicher Menge vor, wohingegen es derzeit an einer hinsichtlich des Monitoring und der dazu angemessenen Auswertungsroutine, geeigneten Verknüpfungen und Abstimmungen zur Tierökologie fehlt.

Zusammenfassend legen die bisherigen Ergebnisse nahe, dass:

- die Datenlage zum Vorkommen der wichtigsten Gruppen der Bodenorganismen auf den BDF nicht ausreichend ist, um diese für ein GVO-Monitoring zu nutzen;
- die bestehenden (sowie die noch zu beprobenden) BDF unter Verwendung einer einheitlichen Biotoptypenliste einzustufen sind;
- weitergehende Untersuchungen (z.B. bessere regionale Abdeckung; Verwendung neuer Determinationsmethoden) auf möglichst repräsentativ ausgesuchten BDF zu empfehlen sind;
- die Grundlagen für ein Referenzsystem geschaffen werden müssen, um die beim GVO-Monitoring erhobenen Daten auch beurteilen zu können.

Im folgenden Kapitel wird zunächst speziell auf die Frage dieses Referenzsystems eingegangen, bevor konkrete Vorschläge zur Weiterentwicklung des BDF-Monitoringprogramms abgegeben werden.

3 Entwicklung konkreter Erweiterungs- oder Anpassungsmöglichkeiten der Boden-Dauerbeobachtung der Länder und / oder ergänzender Monitoringmodule für das GVO-Monitoring

In den bisherigen Kapiteln wurde dargestellt, inwieweit die BDF für ein bodenbiologisches Monitoring von GVO nutzbar sind. Ein Fazit war, dass für ein bundesweites Monitoring die Zahl der bisher dort erhobenen biologischen Daten eindeutig nicht ausreicht. Daher wurde geprüft, ob es weitere, qualitativ ausreichende bodenbiologische Daten von anderen Standorten gibt. Dazu wurde im Rahmen des UBA-Projektes "Erfassung und Analyse des Bodenzustandes im Hinblick auf die Umsetzung und Weiterentwicklung der Nationalen Biodiversitätsstrategie“ eine Literaturrecherche durchgeführt und auch eigene, bisher nicht publizierte Daten verwendet. Alle so erhobenen Daten wurden zusammen mit den organismischen Daten der BDF in die Bo-Info Datenbank aufgenommen, in der nun (Stand Januar 2012) 1.744 Standorte mit über 42.000 Bodentier-Datensätzen enthalten sind. Es wurden chemische und physikalische Bodenparameter, mikrobiologische und Landnutzungsparameter berücksichtigt. Ökologische Charakterisierungen wie z.B. Biotoptyp, Bodentyp, ökologische Raumeinheit und Naturraum sind ebenso eingebunden. Für die Auswertung wurden Biotoptyp, Wasserstoffionenkonzentration (pH), organischer Gehalt (Corg), Kohlenstoff-Stickstoff-Verhältnis (C/N) und Textur des Bodens herangezogen. Im Folgenden wird aufgezeigt werden, dass mit diesem kombinierten Datensatz die ökologisch-räumliche Abdeckung Deutschlands mit bodenbiologischen Daten deutlich verbessert werden konnte (Abb. 32). Es bestehen zwar noch Lücken, wobei allerdings zu unterscheiden ist zwischen Regionen, die bisher nur wenig beprobt wurden (z.B. Mecklenburg-Vorpommern) und denjenigen, deren Daten dem UBA (noch) nicht zur Verfügung gestellt wurden (z.B. Bayern).

Im Folgenden wird zuerst die Ableitung eines Referenzsystems zur Beurteilung der bodenbiologischen Qualität eines Standorts (z.B. im Rahmen eines GVO-Monitoringprogramms) vorgestellt, bevor am Beispiel einzelner Organismengruppen (Lumbriciden, Enchytraeen) konkrete Referenzwerte vorgestellt werden. Dazu wird zuerst auf die Unterschiede zwischen den Biotoptypen 1. Ebene (= Haupt-Landnutzungsformen) eingegangen, bevor gezeigt werden wird, dass sich auch einzelne Ackerbiotoptypen der 2. Ebene anhand bodenbiologischer Daten differenzieren lassen. Mit anderen Worten: es werden erste Teile eines Referenzsystems vorgestellt, mit dem sich auch Auswirkungen von GVOs beurteilen lassen könnten.

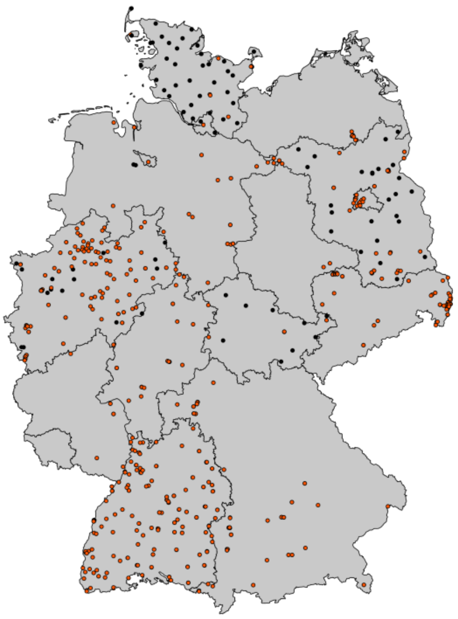

Abb. 32: Standorte zu denen Daten zu Bodenorganismen (primär Collembolen, Oribatiden, Lumbriciden, Enchytraeen) vorhanden sind. Standorte der Boden-Dauerbeobachtung der Länder: schwarz; Standorte der ARGE: rot.

3.1 Konzeptionelle Herangehensweise

Um ökologische Tier-Daten auf Landschaftsebene nutzbar zu machen, bedarf es eines einheitlichen Bezugsystems. Ohne ein solches sind aufgrund der Heterogenität der Landschaft keine vergleichenden Aussagen über Strukturen von Lebensgemeinschaften oder auf sie wirkende Einflussfaktoren möglich. Für eine Bewertung von Veränderungen im Rahmen des GVO-Monitoring erscheint es deshalb essentiell, standortbezogen sogenannte Referenzen (Soll-Werte, Summe der Referenzen=Referenzsystem) zu erstellen. Die Kenntnis dieser Referenzen, d.h. Biozönose-Standort-Beziehungen, macht Bewertungen des ökologischen Zustandes unabhängig von der Höhe der Diversität oder der Zahl der Tiere, die nicht als Maß für die Qualität von Lebensgemeinschaften und Biotopen gelten können. Erst durch die Kenntnis von biotopspezifischen Soll-Werten wird die Ableitungen von Schwellenwerten, die erhebliche Veränderungen der Lebensgemeinschaft anzeigen, möglich (vgl. Abb. 33).

Ein Referenzsystem zum Umgang mit der standortbezogenen Diversität von Bodenorganismen besteht dementsprechend aus:

- Listen von Arten und Spannen von Abundanzen, die an einem Standort mit spezifischen Bedingungen (z.B. Klima, Bodenfaktoren, Region usw.) auftreten sollten;
- einer Vorstellung, ab wann eine Abweichung von dieser Erwartung als negativ zu bewerten ist.

Das GVO-Monitorings sollte demnach ein standortökologisches Konzept verfolgen um standörtliche Parameter mit dem Auftreten von Bodenorganismen zu verknüpfen bzw. in einem System zu integrieren. Somit wird es möglich wesentliche Faktoren zu selektieren und als Kovariablen in einer selektiven statistischen Effekt-Auswertung zu integrieren.

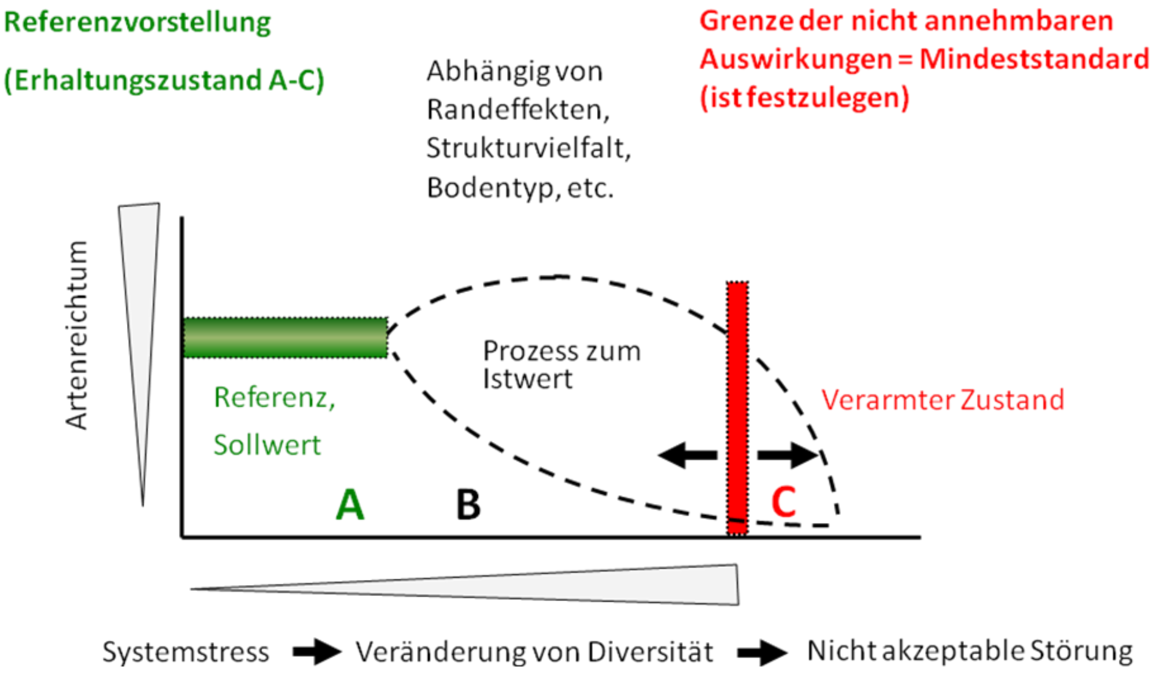

Abb. 33: Prinzip zur Ableitung von Schwellenwerten in Bezug auf Referenzzustände (RÖMBKE et al. 2012): A, B und C entsprechen verschiedenen Erhaltungszuständen in Bezug zum Systemstress (z.B. FFH-Gesetzgebung, EU 1992).

Um eine basale räumlich-ökologische Bezugsgröße zur Bewertung von Effekten zu schaffen, ist eine entsprechende Typisierung der untersuchten Lebensräume unabdingbar. Im oben genannten UBA-Vorhaben wurden die Standard-Biotoptypenliste für Deutschland (RIECKEN et al. 2003), die 44 Basistypen mit ca. 1000 Untertypen umfasst, verwendet und hinsichtlich ihrer Verwendbarkeit erprobt. Ihre Verwendung führt zur Kompatibilität mit anderen Monitoringansätzen, der FFH-Gesetzgebung, des Naturschutz-Managements und voraussichtlich auch der Pestizidregistrierung. Aus der Biotoptypenliste wurden 21 Basistypen mit 525 Untertypen als relevante Standorttypen für die Bewertung der Bodenlebensgemeinschaften extrahiert. In den Analysen der Organismengruppen zeigten sich klare Korrelationen zwischen dem Vorkommen von Arten und der jeweiligen hierarchischen Ebene im System der Biotoptypen-Liste. Die weitergehende Analyse zeigt, dass die Zusammensetzung von Gemeinschaften abhängig ist von den jeweiligen Standortfaktoren, und eine umfassende ökologische Beurteilung von Standorten die Integration verschiedener relevanter Organismengruppen auf Artebene, wodurch auch funktionelle Anforderungen abgedeckt werden, erfordert. Damit werden auch die von einzelnen Organismen erbrachten ökosystemaren Dienstleistungen berücksichtigt.

Nur die integrative Auswertung der Verteilung der Arten über verschiedene Biotoptypen macht es möglich Arten, die für einen bestimmten Biotoptyp charakteristisch sind, zu identifizieren. Das Prinzip des Zusammenhangs zwischen Biotoptypen und Verteilungs-

amplituden einzelner Taxa ist in Abb. 34 visualisiert. Deshalb ist es notwendig, eine für die betrachtete Landschaft oder Gesamtfläche repräsentative Anzahl von Biotoptypen über mehrere Taxa vergleichend zu analysieren.

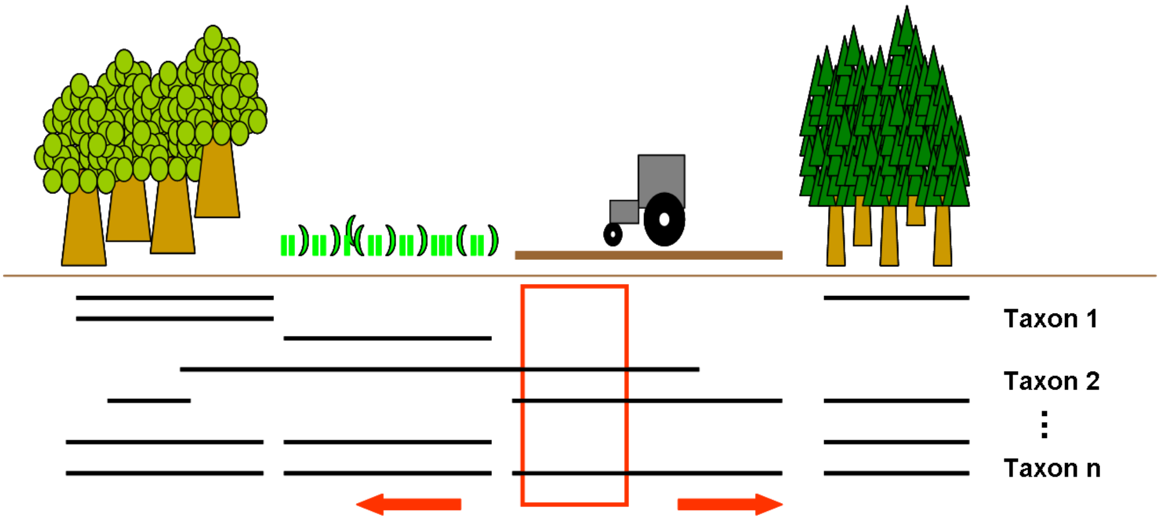

Abb. 34: Schematische Darstellung der Biotoptyp spezifischen Biodiversität am Beispiel eines Ackers, basierend auf den Verteilungsmustern verschiedener Taxa (RÖMBKE et al. 2012).

Trotz recht heterogener Datenlage, mit den aus der Bo-Info Datenbank zur Verfügung stehenden Informationen, konnten wesentliche Grundlagen für die Referenzwertbildung bereits gelegt werden. Bei einheitlicher Datengrundlage, d.h. der standardisierten Erhebung von Bodenorganismen auf BDF, wäre in Zukunft auch eine standardisierte Auswertung der Daten unter anderem für die Zwecke und gemäß den Anforderungen des GVO-Monitorings möglich. Darüber hinaus könnten auch taxonspezifische Besonderheiten durch die Einbeziehung von Kovariablen (z.B. regionale Muster, Vertikalverteilungen) dargestellt werden.

3.2 Einzelne Tiergruppen

Für den im Fokus dieses Projektes stehenden Nutzungstyp Acker beruhen die Ergebnisse mit Ausnahme der Lumbriciden nur auf wenigen Standorten. Es handelt sich somit nicht um eine repräsentative Datenlage zu den jeweiligen Nutzungsformen und somit kann zurzeit keine Differenzierung der jeweiligen Nutzungsformen anhand der Bodenorganismen durchgeführt werden. Die besten Kenntnisse bestehen für Lumbriciden. Da es in Deutschland sehr unterschiedliche, hinsichtlich der Bodenfauna relevante, Acker-Nutzungsformen (Pflugeinsatz, Pfluglose Bewirtschaftung, Kompostdüngung, Mineraldüngung etc.) als auch unterschiedliche Bodenausgangsgesteine (Löss, Sand, Kalk, etc.) auf denen Landwirtschaft betrieben wird gibt, kann von einer weit über die unten dargestellten Ergebnisse hinausgehenden Biodiversität, im Sinne verschiedenartiger Boden-Lebensgemeinschaften, Gemeinschaftsstrukturen etc., ausgegangen werden, die derzeit nur in Ansätzen bekannt ist.

3.2.1 **Collembolen**

In Tab. 5 sind für Deutschland die mittleren Abundanzen und Artenzahlen für Collembolen in den vier Haupt-Landnutzungsformen aufgeführt. Bei dieser Tiergruppe ist aufgrund der Datenlage keine Differenzierung innerhalb der Landnutzungsform Acker möglich. Weitere Probennahmen, vor allem auf BDFs, sind daher für die Nutzung der Collembolen zu empfehlen.

Tab. 5 Mittlere Abundanzen und Artenzahlen der in Bo-Info erfassten Collembolengemeinschaften der verschiedenen Haupt-Biotoptypen. Ebenfalls angegeben sind die Minimum- und Maximalwerte.

	**Laubwald**	**Nadelwald**	**Grasland**	**Acker**
n =	123	58	8	19
Mittlere Abundanz	41.000	47.000	7.900	41.000
Min	5.300	5.000	500	8.400
Max	274.000	215.000	180.000	128.000
Mittlere Artenzahl	18	16	13	4
Min	3	5	3	1
Max	55	33	21	10

3.2.2 **Oribatiden**

In Tab. 6 sind für Deutschland die mittleren Abundanzen und Artenzahlen für Oribatiden in den vier Haupt-Landnutzungsformen aufgeführt. Zudem sind in Tab. 7 die Zeigerarten der Oribatiden für den Landnutzungstyp Acker aufgelistet.

Tab. 6: Mittlere Abundanzen und Artenzahlen der in Bo-Info erfassten Oribatidengemeinschaften der verschiedenen Haupt-Biotoptypen. Ebenfalls angegeben sind die Minimum- und Maximalwerte sowie Referenzwerte für Abundanz und Artenzahl in Abhängigkeit von den verschiedenen Biotoptypen.

	**Laub(misch)wald**	**Nadelwald**	**Grasland**	**Acker**
Anzahl der Standorte	40	8	21	4
**Abundanz**				
Mittelwerte	31.000	46.000	5.800	750
Untergrenze	3.500	14.500	2.300	400
Obergrenze	113.000	125.000	10.000	1.200
**Artenzahl**				
Mittelwerte	53	52	20	7
Untergrenze	25	43	8	4
Obergrenze	92	67	34	10

Tab. 7: Potentielle Zeigerarten (hoch stetig nur im Biotoptyp Acker) und Differentialarten (für bestimmte Faktorenausprägungen innerhalb eines Biotoptyps) der Oribatiden, basierend auf den Ergebnissen der Einzelarten-, Gruppen- und multivariaten Auswertung.

	Zeigerarten	Differentialarten	Ubiquisten
**Äcker**		*Punctoribates punctum (eudom)*<br>*Tectocepheus minor (eudom)*<br>*Tectocepheus velatus (eudom)*<br>*Oppiella falcata (dom)*<br>*Ceratozetes gracilis (subdom)*<br>*Scheloribates laevigatus (subdom)*<br>*Xenillus tegeocranus (subrez)*	*Achipteria coleoptrata*<br>*Oppiella nova (eudom)*<br>*Dissorhina ornata*

Auch für Oribatiden ist aufgrund der Datenlage keine Differenzierung innerhalb der Landnutzungsform Acker möglich.

3.2.3 Lumbriciden

In Tab. 8 sind die mittleren Abundanzen und Artenzahlen sowie die erwarteten Arten für die deutschen Regenwürmer in den vier Haupt-Landnutzungsformen (Biotoptypen) aufgeführt.

Qualitativ unterscheiden sich die vier Nutzungsformen ebenfalls, wobei die deutlichste Trennung (Basis: Vorkommen >50 %) die zwischen Wald- und Offenlandstandorten ist:

Äcker (33.): *A. caliginosa, A. rosea, L. terrestris*

Grasland (34.): *A. caliginosa, L. terrestris, L. rubellus, A. rosea*

Laubwald (43.): *L. rubellus, D. octaedra, D. rubidus*

Nadelwald (44.): *L. rubellus, D. octaedra*

Tab. 8: Erwartete Artenzahl und –zusammensetzung sowie die jeweilige mittlere Abundanz, getrennt nach den vier Landnutzungen / Hauptbiotoptypen (Basis: Bo-Info-Datenbank; Basis: bis zur Art bestimmte Tiere). n = Anzahl der in die Auswertung eingegangenen Stand In Rot: Typische Arten für jeden Nutzungstyp (Vorkommen an > 50% aller Standorte).

	**Äcker (33.) (n = 86)**		**Grasland (34.) (n = 48)**		**Laubwald (43.) (n = 65)**		**Nadelwald (44.) (n = 27)**	
	**Vorkommen**	**Ind./m²**	**Vorkommen**	**Ind./m²**	**Vorkommen**	**Ind./m²**	**Vorkommen**	**Ind./m²**
A. chlorotica	31,4 %	6,4	35,4 %	4,8	0,0 %	0,0	0,0 %	0,0
A. caliginosa	84,9 %	23,1	91,7 %	28,1	36,9 %	5,3	25,9 %	1,7
A. longa	19,8 %	2,6	10,4 %	0,6	3,1 %	0,0	0,0 %	0,0
A. rosea	55,8 %	7,1	56,3 %	6,6	33,8 %	2,0	25,9 %	2,2
D. octaedra	2,3 %	0,0	12,5 %	1,3	72,3 %	5,3	77,8 %	7,8
D. rubidus	0,0 %	0,0	8,3 %	0,7	55,4 %	3,4	29,6 %	0,7
L. castaneus	9,3 %	0,8	31,3 %	2,4	16,9 %	1,3	7,4 %	0,1
L. rubellus	24, %	1,3	62,5 %	10,3	73,8 %	3,7	59,3 %	3,9
L. terrestris	55,8 %	5,2	75,0 %	8,6	20,0 %	0,6	7,4 %	0,1
O. tyrtaeum	17,4 %	0,9	41,7 %	3,6	26,2 %	1,4	25,9 %	1,0
**Σ (Ind./m²)**	**49,3**		**75,6**		**36,6**		**18,3**	
**Artzahl**	**3,3**		**5,0**		**3,9**		**2,9**	

Bei den Äckern lassen sich drei häufige Biotoptypen unterscheiden (Tab. 9). Auf Kalkäckern (Biotoptyp 33.01) sollten mindestens drei Arten (zwei Arten der endogäischen Gattung *Aporrectodea (A. caliginosa, A. rosea)*) sowie die ebenfalls endogäische Art *Octolasion tyrtaeum* mit einer Gesamtabundanz von knapp 30 Tieren/m2 vorkommen. Zudem sollte auf Sandäckern (33.03) immer *A. caliginosa* (Fund auf 100% aller Standorte dieses Typs) auftreten, wobei die mittlere Abundanz der Gemeinschaft bei ca. 19 Tieren/m2 liegt (davon 86 % *A. caliginosa*). Bei Äckern auf Löss-, Lehm- oder Tonböden (33.04) wären mindestens vier Arten zu erwarten, für die außer den beiden *Aporrectodea*-Spezies (s. o.) *A. chlorotica* und *L. terrestris* typisch wären. Auffallend ist hier die hohe mittlere Abundanz von 93 Tieren/m2, der höchsten unter den untersuchten Biotoptypen. Für alle drei Ackertypen gilt, dass acidophile Streuschichtbewohner dort (fast)

völlig fehlen (speziell *D. octaedra* und *D. rubidus*). Dabei fällt allerdings auf, dass die ebenfalls sauren Boden präferierende Art *L. rubellus* durchaus in relevanter Häufigkeit (44 % aller Standorte dieses Typs) auf Kalkäckern vorkommen kann.

Tab. 9: Referenzwert-Vorschlag für die Lumbricidengemeinschaft dreier Ackerbiotoptypen. In Rot: Typische Arten für jeden Biotoptyp (Vorkommen an >50% aller Standorte).

	**„Kalk“ 33.01 (n = 16)**		**„Sand“ 33.03 (n = 21)**		**„Lehm“ 33.04 (n = 31)**	
	**Vorkommen**	**Ind./m²**	**Vorkommen**	**Ind./m²**	**Vorkommen**	**Ind./m²**
A. chlorotica	12,5%	2,9	14,3%	0,3	54,8%	11,3
A. caliginosa	75,0%	6,0	100,0%	16,2	87,1%	45,5
A. longa	6,3%	0,1	4,8%	0,0	41,9%	6,9
A. rosea	75,0%	11,8	14,3%	0,8	87,1%	12,0
D. octaedra	0,0%	0,0	9,5%	0,1	0,0%	0,0
D. rubidus	0,0%	0,0	0,0%	0,0	0,0%	0,0
L. castaneus	12,5%	0,4	0,0%	0,0	16,1%	2,0
L. rubellus	43,8%	1,7	9,5%	0,1	16,1%	1,4
L. terrestris	37,5%	0,9	33,3%	1,4	83,9%	10,9
O. tyrtaeum	62,5%	4,0	0,0%	0,0	12,9%	0,3
**Σ (Ind./m²)**	**28,7**		**18,9**		**93,2**	
**Artzahl**	**3,4**		**1,9**		**4,4**	

3.2.4 **Enchytraeen**

In Tab. 10 sind die mittleren Abundanzen und Artenzahlen sowie die erwarteten Arten für die deutschen Enchytraeen in den vier Haupt-Landnutzungsformen (Biotoptypen) aufgeführt.

Tab. 10: Erwartete Artenzahl und –zusammensetzung sowie die jeweilige mittlere Abundanz, getrennt nach den vier Landnutzungen / Hauptbiotoptypen (Basis: Bo-Info-Datenbank). In Rot: Typische Arten für jeden Nutzungstyp (Vorkommen an >50 % aller Standorte)

Vorkommen (%)	**Äcker (33.)**	**Grasland (34.)**	**Laubwald (43.)**	**Nadel-wald (44.)**
Achaeta aberrans	12,5	5,3	52,9	38,9
Achaeta abulba	8,3	5,3	23,5	66,7
Achaeta bibulba	16,7	5,3	2,9	0,0
Achaeta bohemica	4,2	7,9	17,6	55,6
Achaeta camerani	0,0	0,0	55,9	55,6
Achaeta pannonica	20,8	31,6	0,0	0,0
Buchholzia appendiculata	16,7	63,2	29,4	33,3
Cognettia sphagnetorum	8,3	10,5	94,1	100,0
Enchytraeus buchholzi	95,8	44,7	50,0	0,0
Enchytraeus christenseni	91,7	63,2	29,4	38,9
Enchytraeus lactaeus	50,0	13,2	5,9	0,0
Enchytraeus norvegicus	29,2	34,2	55,9	50,0
Enchytronia annulata	12,5	5,3	0,0	0,0
Enchytronia minor	50,0	31,6	0,0	0,0
Enchytronia parva	16,7	44,7	44,1	38,9
Fridericia benti	12,5	31,6	14,7	0,0
Fridericia bisetosa	25,0	50,0	11,8	16,7
Fridericia bulboides	83,3	86,8	2,9	22,2
Fridericia christeri	70,8	23,7	0,0	0,0
Fridericia deformis	29,2	0,0	0,0	0,0
Fridericia galba	62,5	55,3	23,5	11,1
Fridericia granosa	16,7	0,0	0,0	0,0

Vorkommen (%)	**Äcker (33.)**	**Grasland (34.)**	**Laubwald (43.)**	**Nadel-wald (44.)**
Fridericia isseli	45,8	2,6	0,0	0,0
Fridericia leidigy	20,8	28,9	5,9	0,0
Fridericia paroniana	62,5	15,8	8,8	0,0
Fridericia ratzeli	8,3	65,8	14,7	5,6
Henlea perpusilla	83,3	55,3	2,9	5,6
Henlea ventriculosa	37,5	71,1	0,0	0,0
Marionina brendae	41,7	7,9	2,9	0,0
Marionina clavata	0,0	2,6	73,5	83,3
Oconorella cam-brensis	0,0	0,0	76,5	72,2
**∑ (Ind./m²)**	**20,165**	**13,834**	**51,241**	**52,087**
**Mittl. Artzahl/Standort**	**13,7**	**12,2**	**12,4**	**9,2**

Im Anschluss wird überprüft, inwieweit sich für die Biotoptypen der 2. Ebene für Äcker Referenzwerte für den Endpunkt Artenzusammensetzung angeben lassen. Bei den Äckern und Ackerbrache (Biotoptyp 33.) liegen Daten für „Äcker und Ackerbrache auf Sandboden“ (33.03; 5 Standorte) sowie „Äcker und Ackerbrache auf Löss-, Lehm- oder Tonboden“ (33.04; 13 Standorte) vor (Tab. 11). Quantitativ sind beide Biotoptypen nicht unterscheidbar: die mittlere Abundanz unterscheidet sich um den Faktor 1,5 und die Artenzahl ist mit 14,2 bzw. 14,4 praktisch identisch.

Qualitativ gibt es ebenfalls eine breite Überschneidung: sechs Arten (*E. buchholzi, E. christenseni, E. minor, F. bulboides, F. christeri, H. perpusilla*) kommen auf beiden Standortgruppen mit >50 % Stetigkeit vor. Auf der anderen Seite gibt es aber auch klare Unterschiede (>50 % Vorkommen an Standorten des einen Biotoptyps, an weniger als 20 % Standorten des anderen Biotoptyps):

- nur in den Böden sandiger Äcker treten *A. aberrans, A. bibulba, E. norvegicus, E. annulata, E. parva, F. granosa* und *H. ventriculosa* an mehr als 50 % der Standorte auf;
- Äcker auf Löss-, Lehm- und Tonböden sind dagegen durch das häufige Auftreten von *E. lactaeus, E. minor* und *M. brendae*, vor allem aber mehrere *Fridericia*-Spezies charakterisiert: *F. deformis, F. galba, F. isseli* und *F. paroniana*.

Die Differenzierung tritt also schon auf Gattungsebene auf: Nur in leichten sandigen Böden treten *Achaeta*-Spezies auf, während die schweren Lehmböden einerseits durch die sehr kleinen Art *M. brendae* und eine hohe Zahl von *Fridericia*-Arten gekennzeichnet sind. Diese Aussagen werden allerdings durch die kleine Zahl der untersuchten sandigen Standorte relativiert. In jedem Fall fehlen (fast) immer die für saure Böden mit hohem or-

ganischem Anteil typischen Arten (d.h. primär Streuschichtbewohner), wie z.B. *M. clavata* oder *O. cambrensis*. Schwer erklärbar ist, weshalb an einem Acker-Standort mit sandigem Boden die dominante Art saurer Waldstandorte (*C. sphagnetorum*) nachgewiesen wurde. Eventuell könnte es sich dabei um „Randeffekte“ handeln; d.h. der/die beprobten Standorte grenzen an einen entsprechenden Wald. Zudem mag eine Rolle spielen, dass die Sandäcker im Mittel einen niedrigeren pH-Wert als die Kalk- oder Löss-, Lehm- und Tonäcker haben.

Tab. 11: Erwartete Artenzahl und –zusammensetzung sowie die jeweilige mittlere Abundanz, getrennt nach zwei Acker-Biotoptypen der 2. Ebene, ausgehend von den Angaben in der Bo-Info-Datenbank (Basis: nur bis zur Art bestimmte Tiere). In Rot: Typische Arten für jeden Nutzungstyp (Vorkommen an >50 % aller Standorte).

	**Sand 33.03 (n = 5)**		**Lehm usw. 33.04 (n = 13)**	
	**Vork-ommen**	**Ind./m²**	**Vorkommen**	**Ind./m²**
Achaeta aberrans	60,0 %	1322,8	0,0 %	0,0
Achaeta abulba	40,0 %	714,4	0,0 %	0,0
Achaeta bibulba	60,0 %	4585,5	7,7 %	20,8
Achaeta bohemica	0,0 %	0,0	7,7 %	20,4
Achaeta camerani	0,0 %	0,0	0,0 %	0,0
Achaeta pannonica	40,0 %	396,8	7,7 %	4,4
Buchholzia appendiculata	20,0 %	17,6	7,7 %	866,0
Cognettia sphagnetorum	20,0 %	35,2	0,0 %	0,0
Enchytraeus buchholzi	80,0 %	1357,9	100,0 %	4447,2
Enchytraeus christenseni	100,0 %	12633,9	100,0 %	3762,8
Enchytraeus lactaeus	20,0 %	194,0	69,2 %	287,8
Enchytraeus norvegicus	60,0 %	202,9	7,7 %	34,3
Enchytronia annulata	60,0 %	70,4	0,0 %	0,0
Enchytronia minor	80,0 %	1155,1	53,8 %	712,2
Enchytronia parva	60,0 %	511,4	7,7 %	241,6
Fridericia benti	0,0 %	0,0	15,4 %	101,2
Fridericia bisetosa	20,0 %	17,6	15,4 %	153,7
Fridericia bulboides	80,0 %	2360,5	92,3 %	826,7

	Sand 33.03 (n = 5)		Lehm usw. 33.04 (n = 13)	
	**Vork-ommen**	**Ind./m²**	**Vorkommen**	**Ind./m²**
Fridericia christeri	60,0 %	185,2	84,6 %	629,6
Fridericia deformis	0,0 %	0,0	53,8 %	427,4
Fridericia galba	0,0 %	0,0	84,6 %	430,8
Fridericia granosa	60,0 %	247,0	7,7 %	13,5
Fridericia isseli	0,0 %	0,0	76,9 %	1431,3
Fridericia leidigy	0,0 %	0,0	30,8 %	280,5
Fridericia paroniana	0,0 %	0,0	92,3 %	628,5
Fridericia ratzeli	20,0 %	17,6	7,7 %	3,9
Henlea perpusilla	100,0 %	499,5	84,6 %	981,1
Henlea ventriculosa	80,0 %	493,8	15,4 %	95,0
Marionina brendae	0,0 %	0,0	76,9 %	598,0
Marionina clavata	0,0 %	0,0	0,0 %	0,0
Oconorella cambrensis	0,0 %	0,0	0,0 %	0,0
**Σ (Ind./m²)**	**28.923,5**		**19.685,7**	
**Mittl. Artzahl/Standort**	**14,2**		**14,4**	

3.3 Repräsentativität der im UBA-Vorhaben erfassten Boden-Biodiversität

Eine wesentliche Frage für ein zukünftiges GVO-Monitoring ist, inwieweit die bisher ermittelten Kenntnisse (d.h. Untersuchungen auf bestehenden BDF sowie Literaturdaten aus dem UBA-Vorhaben) das Schutzgut Biodiversität im Boden abdecken. Dazu werden im Folgenden die im UBA-Projekt ermittelten Zahlen zur Artenvielfalt dargestellt und mit den aus Deutschland bisher bekannten Artenzahlen verglichen.

Bei den Collembolen geht man zurzeit von einem Vorkommen von 400-500 Arten in Deutschland aus. Davon wurden 207 Arten (ca. 45 %) im UBA-Vorhaben berücksichtigt. Nach WEIGMANN (2006) sind 630 Oribatidenarten für Deutschland zu erwarten, davon konnten 295 (ca. 48 %) in die vergleichende Auswertung eingebunden werden. Bei den Lumbriciden werden alle in Deutschland nachgewiesenen Arten in der Bo-Info-Datenbank geführt. In Deutschland sind derzeit 127 Enchytraeenarten bekannt (FAUNA EUROPAEA WEB SERVICE 2007), so dass bei 96 in der Datenbank geführten Arten ca. 76 % der deutschen Arten enthalten sind.

Die erfasste Diversität liegt daher bei den beiden Mikro-Arthropoden-Gruppen ungefähr im Bereich von 50 %, bei den Oligochäten-Gruppen bei über 75 % der aus Deutschland bekannten Arten. Damit wird in der Datenbank ein erheblicher Anteil der Bodenbiodiversität dieser vier Gruppen in Deutschland erfasst. Dieser hohe Wert ist überraschend, da durch die BDF und den in der Literatur beschriebenen Standorten viele für Bodentiere relevante Biotoptypen gar nicht abgedeckt werden (allerdings sind gerade die BDF repräsentativ für die Biotoptypen mit den höchsten Flächenanteilen). Diese Ergebnisse deuten darauf hin, dass die bisher untersuchten, größtenteils agrarisch oder forstlich genutzten Standorte (einschließlich der BDF) aufgrund der dort gefundenen relativ hohen Diversität gut dafür geeignet sind, ein Grundraster zum Monitoring der Bodenbiodiversität bzw. für das GVO-Monitoring in Deutschland zu liefern.

3.4 Fazit: Stand der Referenzwertableitung

Zusammenfassend lässt sich festhalten, dass der Ansatz, mögliche Wirkungen von anthropogenen Stressoren (inklusive GVOs) auf Organismengemeinschaften an ausgewählten Ackerstandorten zu erkennen, erfolgversprechend ist (allerdings ist die Datenlage zur Biodiversität gerade auf Äckern noch zu verbessern). Diese Aussage bedarf allerdings noch der Überprüfung unter realistischen Bedingungen. Dabei beruht die Beurteilung nicht auf dem direkten Vergleich zwischen einer bestimmten GVO-Fläche und einer benachbarten BDF, sondern um einen Vergleich zwischen dieser GVO-Fläche und einer Referenz, die wiederum von (u.a.) BDF-Flächen abgeleitet wurde. Dieser Ansatz wird u.a. in Holland für die Beurteilung der Bodenqualität vorgeschlagen (BISQ), wobei eine Vielzahl struktureller und funktionaler Endpunkte eingesetzt wird (u.a. zu mehreren Bodenorganismengruppen). Als Beispiel einer BISQ-Ergebnisdarstellung werden in der nächsten Abbildung (Abb. 35) die jeweiligen Werte von konventionell bewirtschafteten Standorten dargestellt, wobei die Daten einer biologisch arbeitenden Farm als Referenz (= 100 %) dienen. In diesem Fall würden alle Parameter, deren Balken des schwarzen inneren Kreises liegen (z.B. die Biomasse oder Abundanz von Regenwürmern), auf eine Störung des Bodenzustands hinweisen.

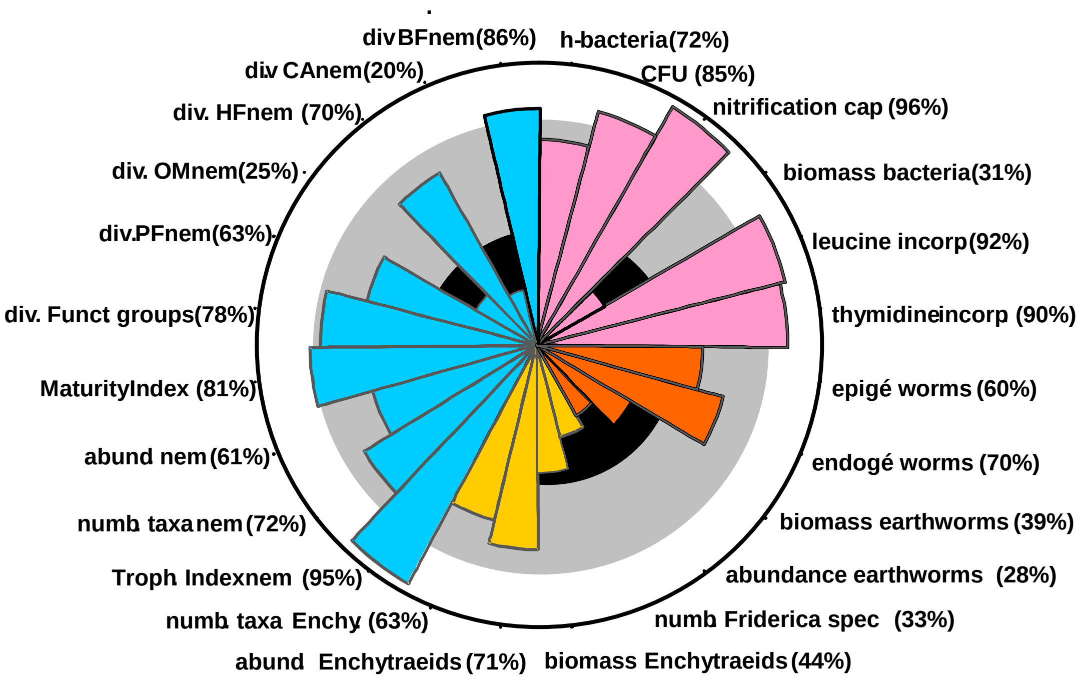

Abb. 35: Gegenüberstellung von Ergebnissen diverser Parameter an konventionell bewirtschafteten Standorten mit denen einer Referenzfläche (biologische Farm). Dabei bedeuten die einzelnen Kreise folgendes: Schwarze Fläche = Differenz >50 %; Graue Fläche = Differenz 25 - 50 %; Schwarzer Ring = 100 % der Referenzfläche (BREURE et al. 2003).

3.5 Vorschläge zur Weiterentwicklung des Monitoring

Zur Etablierung eines GVO-Monitoring der Bodenbiodiversität ist es erforderlich, die Erhebung einer für Deutschland repräsentativen Datengrundlage zur Referenzwertentwicklung für die relevanten Biotoptypen zu schaffen. Auf Basis der eingehenden Analyse kann gesagt werden, das BDF dazu ein geeignetes Grundraster darstellen. 344 von ihnen liegen in Äckern, 146 in Grünland und 247 im Wald (der Rest in Sonderbiotopen). Sie wurden ausgewählt anhand ihrer Repräsentativität für Landnutzung, Naturräume sowie für europäische Klimaregionen und erfüllen damit bereits wesentliche, für ein Monitoring notwendige Voraussetzungen. Die BDF sind aufgrund ihrer großen Zahl und guten Charakterisierung eine hervorragende Basis für ein umfassendes biologisches Monitoring zur nachhaltigen Eignung der Böden als Lebensraum für Bodentiere und Träger natürlicher Bodenfunktionen. Um den Einfluss von GVO auf die Bodenlebensgemeinschaft einzuschätzen ist die Entwicklung von Referenzen auf nicht von GVO beeinflussten Flächen notwendig. Sollen zu diesem Zwecke BDF genutzt werden, wäre es erforderlich, diese vor der Beeinflussung von GVO zu schützen. Somit ist der GVO-Anbau auf einer ausreichenden Zahl von BDF-Flächen auszuschließen und es gilt des Weiteren Schutzzonen, d.h. zu definierende Pufferbereiche ohne GVO-Anbau um die BDF einzurichten. Darüber hinaus gilt es klare Kriterien zu entwickeln (sowohl hinsichtlich räumlicher und zeitlicher Maßstäbe), nach denen entschiedenen wird, ab wann eine BDF-Fläche nicht mehr als GVO freie Referenz angesehen werden kann. Im Anschluss an die Referenzbildung auf Basis der derzeitigen BDF sollte entschieden werden, ob eine Nachbesserung der Re-

präsentativität für spezielle Biotoptypen erforderlich ist und wenn ja, für welche Biotoptypen ggf. neue BDF angelegt werden sollten.

3.5.1 Empfehlungen für ein Minimalprogramm zum GVO-Monitoring von Bodenorganismen

Die im Folgenden aufgelisteten Empfehlungen gelten in weiten Teilen generell für das Monitoring und die Beurteilung der Bodenbiodiversität in Deutschland (vgl. RÖMBKE et al. 2012) und sind somit auch relevante Anforderungen an die Boden-Dauerbeobachtung für das GVO-Monitoring. Empfehlungen für ein Minimalprogramm zum GVO-Monitoring von Bodenorganismen beinhalten im Wesentlichen fünf verschiedene Aspekte:

6. Empfehlungen eines Mindestflächensets: Repräsentativität (z.B. des Biotoptyps, der Bundesländer, Naturräume etc.) eines Flächenrasters sowie die Einbindung weiterer Flächenspezifika, wie etwa der Flächen-Historie oder spezifischen Vorbelastungssituationen.
7. Empfehlung eines Mindestdatensatzes zur Standortcharakterisierung; alle Messungen sollen nach ISO-Richtlinien (oder entsprechenden Standards) durchgeführt werden (ISO (2011), TURBÉ et al. (2010), ENVIRONMENT CANADA (2012)).
8. Empfehlungen eines Methodenstandards zum biologischen Monitoring generell, z.B. zur Organismenauswahl für die ausreichende Darstellung des Schutzgutes Biodiversität sowie räumlich und zeitlich repräsentativer Erfassungsmethoden.
9. Empfehlung eines Methodenstandards zum biologischen Monitoring auf BDF in Bezug auf die Nutzung im GVO Monitoring.
10. Empfohlene Merkmale eines abgestimmten Auswertungskonzeptes: Standards zur Datenhaltung und Möglichmachung von standardisierten Auswertungen.

Zu 1. Empfehlung eines Mindestflächensets:

- Rastergrundlage der Flächen auf BDF, repräsentativ über Deutschland verteilt;
- Länderübergreifende Verteilung der Flächen, zentrale Koordination zur abgestimmten Vorgehensweise sinnvoll, z.B. LABO;
- Biotoptypen Laub- und Nadelwald, Grünland, Acker sollen nachfolgende Punkte integrieren: 4 Typen, 4-5 Untertypen mit jeweils 10 Standorten (ca. 160-220 BDF), eine Probenahme-Serie über max. 5 Jahre;
- Bei der Flächenauswahl zu berücksichtigende Kriterien:
  - Abdeckung der in Deutschland relevanten
    - pH-Werte (Bandbreite)
    - Bodenart (Sand/Schluff/Lehm/Ton)
    - Oberbodenverhältnisse (Humusform / Streuauflage/Mineralboden)
    - Naturräume (Mittelgebirgs- und Flachlandausbildung)
  - Integration in europäische Monitoring-Programme.

Eine Wiederholung der Beprobung im Zyklus von 3-5 Jahren wird vorgeschlagen, da dieser Zeitraum das Erkennen von Veränderungen im Zeitraum der Zulassung des GVO möglich macht. Um das Monitoringprogramm für spezielle Fragestellungen im GVO-Monitoring sinnvoll intensivieren oder erweitern zu können, kann das Minimalprogramm auf verschiedenen Skalenebenen erweitert werden. Zum einen durch eine zeitlich intensivere Beprobung (z.B. jährliche Wiederholung) oder einer Verdichtung des Beprobungsrasters auf ausgewählten Flächen z.B. in Gebieten mit erhöhtem GVO-Anbau. Verdichtungen führen zum einen zu einer stärkeren Aussagekraft der Ergebnisse hinsichtlich der statistischen Power, machen aber auch räumlich wie zeitliche Unterschiede (z.B. kurzfristige Veränderungen) sichtbar. Entsprechend kann auch die notwendige Schaffung von Vergleichsdatensätzen in Räumen ohne GVO-Anbau eine zeitlich wie räumliche intensivere Beprobung erforderlich machen.

Zu 2. Empfehlung eines Methodenstandards zur Mindeststandortcharakterisierung:

- pH-Wert (CaCl2, KCl);
- Gehalt an organischem Kohlenstoff;
- Kationenaustauschkapazität;
- Elektrische Leitfähigkeit;
- Trockenmasse;
- Korngrößenverteilung;
- Bodendichte.

In Hinsicht auf die biologische Zielstellung sollten zudem erfasst werden:

- Stickstoffgehalt;
- C/N-Verhältnis;
- maximale Wasserhaltekapazität;
- (primär an Waldstandorten): Humusform.

Darüber hinaus sollten die folgenden Standorteigenschaften mit aufgenommen werden:

- Standorthistorie (z.B. Nutzung, frühere Aufnahmen usw.);
- Bodenkundliche Kennwerte zu Bodentyp (z.B. Braunerde, Lage des Stauwasserhorizontes etc.)
- Physikalischer Stress Bodenverdichtung, Düngung, Erosion usw.
- Genaue geographische Lage (allgemeines Koordinatensystem);
- Landnutzungsform;
- Klimatische Daten; im Minimum:
  - Jahresmittelwert der Lufttemperatur und des Niederschlags, möglichst auch im Jahresgang;

- Jahresmittelwert der Temperatur des Oberbodens, möglichst auch im Jahresgang.

- Grundwasserlevel;
- Angaben zur anthropogenen Belastung:
- Konzentration der wichtigsten Schadstoffe (z.B. LABO-Liste : Schwermetalle sowie ausgewählte Organika);

Zu 3. Empfehlung eines Methodenstandards zum biologischen Monitoring:

Aus Gründen der Praktikabilität ist es nicht möglich alle Bodenorganismen-Gruppen eines Ökosystems zu erfassen. Allein in einem sehr homogenen Lebensraum wie den grasigen Feldrainen ist mit über 1000 Invertebraten-Arten aus unterschiedlichen Tiergruppen zu rechnen (Roß-Nickoll et al. 2004). Aufgrund dieses Umstandes ist es wichtig für ein praktikables Monitoring, sich aus der Vielzahl, relevante Tiergruppen bzw. Taxa auszuwählen.

VDI-Richtlinie 4331 (VDI 2012)

Eine konkrete Vorgehensweise zur Auswahl geeigneter Organismengruppen wird in der VDI-Richtlinie 4331 „Monitoring der Wirkungen von gentechnisch Veränderten Organsimen (GVO)-Wirkung auf Bodenorganismen“ für die Zwecke des GVO Monitorings vorgeschlagen (VDI 2012, Ruf et al. 2013). Die in dieser Richtlinie verwendeten Auswahlkriterien (vgl. Kap. 2.7) beziehen sich auf ihre Repräsentativität für die Biodiversität des Ökosystems, dem Informationsgehalt, der mit ihnen erreicht werden kann sowie auf ihre Aussagekraft hinsichtlich GVO spezifischen Effekten (Abb. 36). Es ist anzumerken, dass im Gegensatz zur vorliegenden Studie in der Richtlinie auch epigäische Raubarthropoden (z.B. Carabiden, Araneae) mit einbezogen wurden. In der Richtlinie werden folgende Arthropodengruppen für ein Monitoring vorgeschlagen: Araneae, Carabidae, Carabiden-Larven, Chilopoda, Diplopoda, Gastropoda, Isopoda, Opilionida, Staphylinidae, Dipteren-Larven, Collembola, Enchytraeidae, Gamasina, Lumbricidae, Nematoda, Oribatida. Da nicht von jeder Stelle und für jede Fragestellung dieselben Tiergruppen gleichrangig bearbeitet werden können wurde eine Auswahlmatrix beschrieben, die es ermöglicht eine, der Fragestellung und der Expertise des Untersuchers angepasste Auswahl zu treffen. Grundsätzlich werden zunächst drei unterschiedliche Lebensraumtypen unterschieden Acker/Grasland und Hecken- und Waldstandorte. Danach werden drei Kriterien zur Auswahl angewendet:

I. Praktikabilität - Kenntnisstand, Vorhandensein von Standardmethoden, Handhabungs-aufwand der jeweiligen Tiergruppe)
II. Informationswert - Maß für die Möglichkeit, eine Zönose durch ökologische Gruppierungen und Differenzierungen der Art Ebene zu gliedern
III. Kategorisierung der Taxa hinsichtlich des Nahrungswahlverhaltens (Pilz- und Bakterienfresser, Pflanzenfresser und Räuber)

Prinzipiell sind diese Organismengruppen frei kombinierbar wobei für ein breit angelegtes Monitoring folgende Regeln einzuhalten sind:

1. Vier Taxa müssen mindestens untersucht werden

2. Unter diesen Taxa müssen alle Ernährungstypen vertreten sein
3. Am besten vier Taxa, mindestens jedoch zwei Taxa mit hohem Informationsgehalt im jeweiligen Biotoptyp müssen berücksichtigt werden
4. zwei epigäische und zwei endogäische Artengruppen müssen enthalten sein

Ergänzend:

- besteht eine bekannte spezifische Wirksamkeit des GVO oder seiner Produkte (z.B. Cry-Protein) auf taxonomische Gruppen, muss ein Vertreter dieser Gruppe aufgenommen werden
- besteht eine stoffliche Exposition in bestimmten trophischen Stufen, muss dies bei der Auswahl berücksichtigt werden
- Es sollten mindestens zwei Vertreter praktikabel, das heißt günstig bzw. eher günstig zu bearbeiten sein

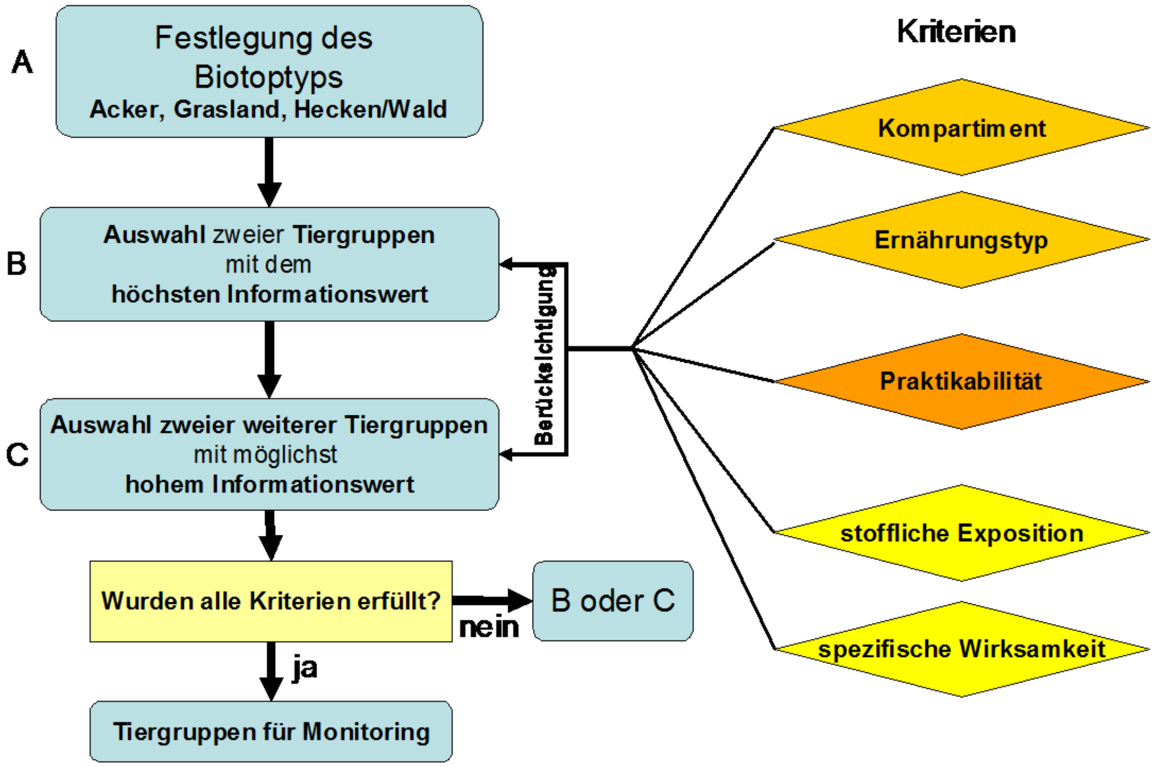

Abb. 36: Entscheidungsbaum zur standardisierten Auswahl von geeigneten Organismen-Gruppen zum GVO Monitoring gemäß VDI-Richtlinie 4331 (VDI 2012).

Zu 4. Empfehlung eines Methodenstandards zum biologischen Monitoring auf BDF in Bezug auf die Nutzung im GVO Monitoring:

Entsprechend der oben aufgeführten VDI-Richtlinie sollte die Organismenauswahl nach Bedarf den speziellen Fragestellungen hinsichtlich Exposition und spezifischer Wirkung des GVO angepasst werden. Es sollte bei dieser Anpassung darauf geachtet werden, dass kontinuierliche Datensätze erzeugt werden, die einen zeitlichen Verlauf abbilden können. Ein ständiger, nicht abgestimmter Wechsel der Erhebung von unterschiedlichen Datensätzen würde zur Fragmentierung dieser Datensätze führen und somit eine zeitli-

che Auswertung verhindern. Zudem dienen kontinuierliche Datenerhebungen dazu, Erfahrungen hinsichtlich der Biotopabhängigkeit von Arten und Artengruppen sowie ihrer Abhängigkeit von Umweltparametern zu sammeln. Diese Erfahrungen sind für die Erstellung von Referenzwerten zur Bemessung und Bewertung von Veränderung dringend erforderlich. Den Ergebnissen eines Literaturreviews zu Effekten von GVO auf Bodenorganismen Rechnung tragend (vgl. Kap. 2.7) erscheint es wesentlich, das Monitoring nicht auf Summenparameter wie z.B. "Gesamtanzahl Collembola" zu beziehen. Effekte von Umwelteinwirkungen erscheinen artspezifisch. Artspezifische Analysen sind anderen Methoden somit vorzuziehen.

Im vorliegenden Projekt liegt der Fokus auf den endogäischen Tiergruppen. Im Rahmen des GVO-Monitorings ist der Vergleich von Datensätzen auf GVO-Flächen bzw. im Einflussbereich von GVO-Flächen mit direkten Referenzflächen, d.h. erhobenen Datensätzen außerhalb des GVO-Einflussbereiches erforderlich. Nach derzeitigem Stand liegen keine geeignete Datensätze zu den verschiedenen endogäischen Tiergruppen auf BDF Standorten vor. Die wohl geeignetsten Datensätze außerhalb der BDF, die für eine Referenzbildung herangezogen werden können bestehen für die Tiergruppen der Lumbriciden, Enchytraeiden, Collembolen und Oribatiden. Diese Organismengruppen werden entsprechend für das Monitoring vorgeschlagen (Erweiterung "ENVASSO Tier 1"). Die Probenahme sollte zeitlich an die ökologischen Bedingungen der Tiere angepasst werden und auf Standards basieren (ISO-Richtlinie); Vertikalverteilung, Streuauflage/Boden sind zu berücksichtigen.

Tiergruppen spezifische Erhebungen für die Referenzwertbildung müssen nach einheitlichen Standards erfolgen. Zudem müssen die auf GVO-Flächen durchgeführten Erhebungen immer mit denselben Methoden, basierend auf denselben Standards erhoben werden, um einen Vergleich mit den Referenzen möglich zu machen.

Mit zu den methodisch erforderlichen Basisdaten gehört eine standortökologische Ansprache der genauen Untersuchungsfläche. Hierzu zählt die genaue Angabe des Biotoptyps, der Vegetationseinheit, sowie des Pflanzenbestandes. Mit Hilfe dieser Angaben ist es möglich standortökologische Bezüge herzustellen, die den Vergleich von Standorten (z.B. Sandäcker) auch überregional möglich machen. Daneben ist es jedoch erforderlich regionale Muster, die u.a. durch die Verbreitungsmuster von Arten, die klimatischen Bedingen etc. hervorgerufen werden, zu erfassen. Deren Berücksichtigung minimiert die Varianz der Datensätze und schärft somit die Aussagekraft gegenüber dem Einfluss des GVO. Diese Daten liegen auf derzeitigen BDF nicht durchgehend vor.

Auf Basis der heutigen Kenntnis zur Verteilung von Bodenorganismen in Ackerböden, können keine Unterschiede von Lebensgemeinschaften in Abhängigkeit zum jährlichen Kultivar (Raps, Zuckerrübe, etc.) ausgemacht werden. Die Bodenlebensgemeinschaft adaptiert sich somit wahrscheinlich an eine langjährige Nutzungsfolge im Agrarsystem an. Generell ist zudem die Verteilung von Bodenorganismen viel stärker mit Bodeneigenschaften wie pH-Wert, Bodenart oder organischem Gehalt, als mit der darüber befindlichen Vegetationsschicht korreliert (JÄNSCH et al. 2011, RÖMBKE et al. 2012). Somit erscheint zunächst die Bildung von Referenzwerten für den Biotoptyp z.B. sandiger Acker unabhängig vom Kultivar sinnvoll. Bei wachsender Kenntnis und steigenden Datensätzen kann eine kultivarspezifische Referenzbildung jedoch durchaus sinnvoll werden.

Zu 5. Empfohlene Merkmale eines abgestimmten Auswertungskonzeptes:

Für die Auswertung von komplexen Datensätzen ist eine standardisierte, abgestimmte Vorgehensweise erforderlich. Die Zahl der statistischen Verfahren zur Berechnung von Unterschieden oder Korrelationen ist groß. Bei der Datenaufarbeitung (z.B. Rechnung mit Absolutzahlen, Log-transformation von Zähldaten, Berechnung Präsenz/Absenz) bis hin zur verwendeten Methode der Vergleichsberechnung (z.B. multivariate Berechnung mit R, Matlab, Canoco, etc.) kann es Unterschiede geben. Häufige Schwierigkeiten bei der Vergleichbarkeit von Datensätzen liegen schon allein in der unterschiedlichen Datenhaltung begründet, bei der es darauf ankommt die Auswertungsmöglichkeit im Datensatz durchgängig zu halten. Ist dies nicht der Fall (Häufig bei z.B. der Datenhaltung in Excel-Tabellen) ist eine flexible Auswertung nicht bzw. nur unter erheblichem Aufwand möglich. Werden Daten an verschiedenen Stellen verwaltet ist eine Abstimmung der Datenbanken erforderlich. Dieser Erfahrung Rechnung tragend werden folgende Empfehlungen gemacht:

- Zentrale Verwaltung/Haltung der Daten in einer (Meta-)Datenbank
- Erhebung vollständiger Datensätze mit allen Parameter- und Organismendaten
- Präsenz/Absenz- und Abundanz-Auswertung
- Gemeinschaftsauswertung unter Berücksichtigung der Kovarianzen (Anwendung multivariater Statistik in einem Analysen-Modul)
- Einbeziehung von ökologischen Hintergrundinformationen

Bezüglich der Datenauswertung bedarf der Begriff der „schädlichen Bodenveränderung" im Kontext des GVO-Monitoring zunächst der Erläuterung und Konkretisierung. Bislang gibt es keine Vorstellung von Lebensraum spezifischen Referenzwerten für einzelne Organismen. Diese gilt es anhand des oben skizzierten Monitoring-Konzepts zu entwickeln. Der erste Schritt der Auswertung ist immer zunächst die einfache Feststellung einer Abweichung, erst danach erfolgt eine Beurteilung derselben anhand vorab festzulegender Schwellen und die abschließende Bewertung durch die kompetente Behörde. Es gibt bislang keine Regelungen bezüglich Effekten auf die Bodenbiozönose im BBodSchG oder anderen Gesetzen, sondern nur Vorsorge-, Prüf- und Maßnahmenwerte für stoffliche Parameter (d.h. bestimmte Konzentrationen einzelner Chemikalien dürfen in einem Boden bestimmter Nutzung nicht überschritten werden (BBodSchV 1999)). Hierbei stellt sich die Frage, wie man sich anzunehmenden Schwellenwerten nähert. Bei der IOBC werden zur Chemikalienbeurteilung z.B. 30 - 50% Effekt (= Abundanzrückgang einer Art (meist Arthropoden)) im Halbfreiland- oder Freilandtest angesetzt (Candolfi et al. 2000). Bei realitätsnäheren Studien (z.B. von Halbfreilandsystemen) geht die Tendenz eher zur 50% Schwelle, während im Freiland die Fähigkeit der Wiederbesiedlung innerhalb eines Jahres oder sogar „Expert Judgement" herangezogen wird.

3.5.2 Weitere Empfehlungen

Die Zielsetzung des GVO-Monitorings überschneidet sich in vielen Bereichen mit denen anderer Programme. Der Kern eines biologischen Monitoring sollte neben einem direkten Nachweis von Veränderungen auch darin liegen systematisch Daten und somit Erfahrun-

gen zu den verschiedenen Tiergruppen zu sammeln. Diese Daten und Erfahrungen können dann auch in anderen Bereichen, in denen Biodiversität im Blickfeld der Betrachtung liegt, von hohem Nutzen sein. Das Programm der BDF in Deutschland stellt neben der Nutzbarkeit für das GVO Monitoring einen Rahmen dar, der unter bestimmten Erweiterungen ebenfalls geeignet ist, die Umsetzung der Strategie zur biologischen Vielfalt und Maßnahmen zum Schutz und zur Förderung der Bodenorganismen zu realisieren. Das bestehende BDF-Programm integriert bereits wichtige ökosystemare Dienstleistungen des Bodens.

Basierend auf diesem Konzept sollten in Deutschland die bisherigen Zielstellungen

- die Charakterisierung des Bodens als Ergebnis von Bodenprozessen und äußeren Einflüssen sowie die Ermittlung und Bewertung von Veränderungen des Bodenzustands;
- die Identifizierung von Möglichkeiten zur Verhinderung von äußeren Einflüssen auf Böden mit dem Ziel des nachhaltigen Schutzes von Bodenfunktionen;
- die Abschätzung der Verlagerung von Bodenschadstoffen in Pflanzen bzw. in das Grundwasser, sowie der Einfluss von Chemikalienimmissionen auf den Boden (nur auf ausgewählten Standorten);
- die wissenschaftliche Nutzung zur Verbesserung des Monitoring sowie zum besseren Verständnis ökologischer Zusammenhänge

des Monitoring auf den BDF um folgende Zielstellungen erweitert werden: Erstellung eines Referenzsystems, dass die Organismen-Gemeinschaften in hinreichendem Maße nutzt, um Schwellenwerte für unakzeptable Veränderungen anzuzeigen. Dies wäre nutzbar nicht nur für das GVO-Monitoring sondern u.a. für das „Risk Assessment“ von z.B. Emissionen (Radioaktivität), Pestiziden, Nährstoffen etc. Eine inhaltliche Verbindung von nationalen und internationaler Monitoring Systemen aus den verschieden Themenfeldern mit dem Ziel einer einheitlichen Basis zur Bewertung von schädlichen Einflüssen könnte enorme Ressourcen sparen.

4 Prüfung der Datenverfügbarkeit sowie von Möglichkeiten der Datenauswertung für das GVO-Monitoring

Die BDFs und damit die auf ihnen erhobenen Daten unterliegen der Hoheit der Bundesländer. Die fachliche Koordinierung erfolgt im Rahmen der Bund-Länder-Arbeitsgruppe Bodenschutz (LABO): Die Geschäftsstelle wechselt alle 2 Jahre, daher ist die Geschäftsstelle zu erreichen unter: https://www.labo-deutschland.de/Kontakt.html.

Es gilt zunächst auf Bund-Länder-Ebene die Frage zu beantworten, ob die oben geforderte Einrichtung von Schutzzonen (kein GVO-Anbau auf und im Umfeld von BDF) mit dem eigentlichen BDF-Konzept vereinbar ist. Die BDF wurden ursprünglich eingerichtet, um den aktuellen Zustand der Böden zu erfassen, ihre Veränderungen langfristig zu überwachen und Entwicklungstendenzen abzubilden. Hierbei stehen bislang stoffliche Belastungen aus diffusen Quellen im Mittelpunkt. Die Betrachtung der Bodenbiologie und die Ableitung von Referenzwerten zur Beurteilung eventueller Effekte durch GVO als konkrete Belastungsquelle stellen hingegen eine neue Fragestellung dar.

Eine Nutzbarkeit der Daten für ein bundesweites Monitoring von GVO erfordert eine standardisierte, für alle Länder gleiche bzw. vergleichbare Datenerhebung und Datenführung, so dass Ergebnisse zentral (z.B. naturräumlich, kulturabhängig etc.) ausgewertet werden können (PLACHTER et al. 2002; TOSCHKI 2008; ZÜGHART et al. 2008). Eine Zusammenführung der bisher aufgenommen bodenkundlichen, klimatischen sowie mikrobiellen Daten aus den Boden-Dauerbeobachtungs-Untersuchungen findet derzeit im Umweltbundesamt statt. Die dort bisher zu diesem Zweck geführte Datenbank ist jedoch nicht für die Erfassung der bodenfaunistischen Daten (bisher nur Lumbriciden und Enchytraeen in einzelnen Bundesländern) ausgelegt. Daher wurden die bislang verfügbaren BDF- sowie weitere Daten aus der Literatur und aus Forschungsprojekten auch für andere Organismengruppen in die Bo-Info Datenbank aufgenommen. Mittlerweile ist auch eine Überführung in die vom BMBF geförderte Senckenberg-Datenbank ‚Edaphobase' (edaphobase.org; Burkhardt et al. 2013) erfolgt, wodurch die Daten auch im Global Biodiversity Information Facility (GBIF; gbif.org) sichtbar sind. Für die Nutzung und Auswertung im Rahmen des GVO-Monitoring bedarf es daher der Verknüpfung der verschiedenen Datenbanken, z.B. im Rahmen einer zentralen Meta-Datenbank. Hier müssen künftig die folgenden Fragen beantwortet werden können:

- Wer ist für die Dauerpflege zuständig und wer stellt die notwendigen Mittel zur Verfügung?
- Wer verfügt über die Datenhoheit?
- Wer ist unter welchen Bedingungen nutzungsberechtigt?

Da die Hoheit der Boden-Dauerbeobachtung bei den Ländern liegt gilt es darzustellen, wie künftig ein kontinuierlicher Datenfluss mit dem Ziel der zentralen Nutzung und Auswertung von Daten für das GVO-Monitoring koordiniert und zwischen den jeweiligen Länder-Programmen harmonisiert werden kann. Hierbei ist zu beachten, dass:

- die verschiedenen Länder-Programme derzeit unterschiedliche Strukturen hinsichtlich der Datenerhebungen, Datenhaltung/Datenverfügbarkeit und Datenauswertung besitzen;

- die Daten erst über langfristige, vergleichbare Erhebungen Wert bekommen. Wichtig ist daher eine Kontinuität bei der Datenhaltung, was durch eine koordinierende zentrale staatliche Stelle am besten gewährleistet werden kann; ein zentrales Datenhaltungskonzept ermöglichen muss, standardisierte, einheitliche und vergleichbare Daten, die für die Bewertung in einem GVO-Monitoring notwendig erscheinen, zu analysieren und diskutieren;
- neben den oben genannten Mindeststandards für die Datenerhebung auch Mindeststandards für die Datenweitergabe und Datenhaltung vorgegeben werden müssen.

Hinsichtlich der eigentlichen Durchführung einer länderübergreifenden Datenauswertung ist die Benennung kompetenter und unabhängiger Gremien erforderlich. Hierbei könnte die bewertende Behörde beispielsweise auf die bereits bestehende Arbeitsgruppe zur Auswertung der BDF-Daten, bestehend aus Vertretern von Bundes- und Landesbehörden, zurückgreifen. Für Länderübergreifende Auswertungen ist das Umweltbundesamt (UBA) Ansprechpartner, speziell das Fachgebiet II 2.7 "Bodenzustand, Bodenmonitoring" (Dr. Frank Glante, Umweltbundesamt, Wörlitzer Platz 1, D06844 Dessau-Roßlau; Tel: 0340 2103 3434; e-mail: frank.glante@uba.de; info@umweltbundesamt.de; http://www.umweltbundesamt.de/boden-und-altlasten/index.htm).

Im Auswertungskonzept sollten auch andere Nutzungsoptionen wie z.B. für die Risikobewertung von Pestiziden, die gleiche oder ähnliche Schutzziele verfolgen, in die Betrachtung eingebunden werden. Eine sinnvolle Verknüpfung von prospektiver und retrospektiver Risikobewertung sollte dabei angestrebt werden, um auch zukünftige Optionen entsprechend ihren Potentiales anzusprechen. Eine wesentliche Rolle wird dabei aller Voraussicht nach die zunehmende Verwendung von computergestützten Modellen zur Risikoanalyse sein (EFSA 2010, Galic et al. 2010, FORBES et al. 2009, GRIMM et al. 2009, PREUSS et al. 2009).

5 Literatur

AG BODEN (1994): Bodenkundliche Kartieranleitung, 4. Auflage. Hannover (BGR).

AG BODEN (2005): Bodenkundliche Kartieranleitung, 5. Erweiterte Auflage. Hannover (BGR).

AHL GOY, P., WARREN, G., WHITE, J., PRIVALLE, L., FEARING, P. & VLACHOS, D. (1995): Interaction of an insect tolerant maize with organisms in the ecosystem. In: LANDSMANN, J. & CASPER, R. (Hrsg.): Key biosafety aspects of genetically modified organisms. Workshop 10.-11. April 1995 in Braunschweig, Germany. - Berlin (Blackwell Wissenschafts-Verlag): S. 50-53.

ANDOW, D.A., HILBECK, A. (2004): Science-based risk assessment for non-target effects of transgenic crops. BioScience 54: 637-649.

ASHAUER, R., AGATZ, A., ALBERT, C. et al. (2011): Toxicokinetic-toxicodynamic modeling of quantal and graded sublethal endpoints: A brief discussion of concepts. Environmental Toxicology and Chemistry 30:2519-2524.

BARTH, N., BRANDTNER, W., CORDSEN, E. et al. (2000): Boden-Dauerbeobachtung. Einrichtung und Betrieb von Boden-Dauerbeobachtungsflächen. In: ROSENKRANZ, D., BACHMANN, G., KÖNIG, W., EINSELE, G. (Hrsg.): Bodenschutz. Berlin (Erich Schmidt Verlag). 32. Lfg., XI/00, 127 S.

BAUMGARTE, S., TEBBE, C. (2005): Field studies on the environmental fate of the Cry1Ab Bt-toxin produced by transgenic maize (MON810) and its effect on bacterial communities in the maize rhizosphere. Molecular Ecology 14: 2539-2551.

BBODSCHG (1998): Gesetz zum Schutz vor schädlichen Bodenveränderungen und zur Sanierung von Altlasten (Bundes-Bodenschutzgesetz). Bundesgesetzblatt I, 502 vom 17. März 1998.

BBODSCHV (1999): Bundes-Bodenschutz- und Altlastenverordnung. Verordnung zur Durchführung des Bundes-Bodenschutzgesetzes. BGBl I, 36, S. 1554 – 1582 vom 16.06.1999.

BECK, L. (1993): Zur Bedeutung der Bodentiere für den Stoffkreislauf in Wäldern. Biologie in unserer Zeit 23: 286-294.

BECK, L., RÖMBKE, J., RUF, A. et al. (1999): Auswirkungen des Einsatzes von Dimilin und *Bacillus thuringiensis* auf die Struktur und Funktion der Bodenfauna. Die Schwammspinner-Massenvermehrung in Baden-Württemberg 1993/94. Freiburger Forstliche Forschung 13: 74-101.

BHATTI, M. A., J. DUAN, G. HEAD, C.J. JIANG, M.J. MCKEE, T.E. NICKSON, C.L., PILCHER C.D. (2005): Field evaluation of the impact of corn rootworm (Coleoptera: Chrysomelidae)-protected Bt corn on ground-dwelling invertebrates. Environ. Entomol. 34: 1325-1335.

BIOSICHERHEIT (2010): Coexistence of genetically modified and nongenetically modified maize: Making the point on scientific evidence and commercial experience. http://www.pgeconomics.co.uk/pdf/Co-existence_maize_10october2006.pdf, letzter Aufruf 28.05.2010.

BIRCH, A.N.E., GEOGHEGAN, I.E., GRIFFITHS, D.W., MCNICOL, J.W. (2002): The effect of genetic transformations for pest resistance on foliar solanidine-based glycoalkaloids of potato (*Solanum tuberosum*). Annals of Applied Biology 140: 143-149.

BIRCH, A.N.E., GRIFFITHS, B.S., CAUL, S. et al. (2007): The role of laboratory, glasshouse and field scale experiments in understanding the interactions between genetically modified crops and soil ecosystems: A review of the ECOGEN project. Pedobiologia 51: 251-260.

BOUCHÉ, M.B. (1977): Strategies lombriciennes. Ecological Bulletin 25: 122-132.

BREURE, A.M., RUTGERS, M., BLOEM, J. et al. (2003): Ecologische kwaliteit van de bodem. RIVM report 607604005 (32 S).

BROOKS, D.R., BOHAN, D.A., CHAMPION, G.T. et al. (2003): Invertebrate responses to the management of genetically modifies herbicide-tolerant and conventional spring crops. I Soil-surface-active invertebrates. Philos. T. Roy Soc. B 358: 1847-1862.

BURKHARDT, U., RUSSELL, D.J., BURYN, R. et al. (2013): The Edaphobase project of GBIF-Germany – A new online soil-organism zoological data warehouse. Eingereicht bei Applied Soil Ecology.

CANDOLFI, M.P., BLÜMEL, S., FORSTER, R. et al. (Hrsg.) (2000): Guidelines to evaluate side-effects of plant protection products to non-target arthropods. IOBC, BART and EPPO Joint Initiative. IOBC/WRPS, Gent, Belgium, 158 S.

CANDOLFI, M. P., BROWN, K., GRIMM, C. et al. (2004): A faunistic approach to assess potential side effects of genetically modified Bt-corn on nontarget athropods under field conditions. Biocontrol Sci. Techn. 14: 129-170.

CHAMBERLAIN, P.M., BULL, I.D., BLACK, H.J. et al. (2006): Collembolan trophic preferences determined using fatty acid distributions and compound-specific stable carbon isotope values. Soil Biol. Biochemistry 38: 1275-1281.

CORTET, J., ANDERSEN, M.N., CAUL, S. et al. (2006): Decomposition processes under Bt (*Bacillus thuringiensis*) maize: Results of a multi-site experiment. Soil Biology and Biochemistry 38: 195-199.

DELB, H., MATTES, J. (2001): Monitoring of *Melolontha hippocastani* F. at the Upper Rhine Valley, Germany. Journal of Forest Science 47 (Special Issue No. 2):70-72.

DOLEZEL, M., HEISSENBERGER, A., GAUGITSCH, H. (2005): Ökologische Effekte von gentechnisch verändertem Mais mit Insektizidresistenz und/oder Herbizidresistenz. Umweltbundesamt Wien.

ENVIRONMENT CANADA (2012): Guidance Document on the Sampling and Preparation of Contaminated Soil for Use in Biological Testing. EPS 1/RM/53, 222 p.

EC (EUROPEAN COMMISSION). (2001): EU Directive 2001/18/EC of 12 March 2001 on the deliberate release into the environment of genetically modified organisms and repealing Council Directive 90/220/EC. Official Journal of the European Communities L 106/1-38 (17.4.2001).

EFSA (EUROPEAN FOOD SAFETY AUTHORITY) (2005): Guidance document of the scientific panel on genetically modified organisms for the risk assessment of genetically modified plants and derived food and feed. The EFSA Journal 99: 1-94.

EFSA (2010): Scientific Opinion on the development of specific protection goal options for environmental risk assessment of pesticides, in particular in relation to the revision of the Guidance Documents on Aquatic and Terrestrial Ecotoxicology (SANCO/3268/2001 and SANCO/10329/2002). EFSA Journal. E. P. o. P. P. P. a. t. R. (PPR). 8(10).

ENGELMANN, H.-D. (1978): Zur Dominanzklassifizierung von Bodenarthropoden. Pedobiologia 18: 378-380.

ESCHENBACH, C., WINDHORST, W. (2009): Indikatoren für die nationale Strategie zur biologischen Vielfalt: Gentechnik in der Landwirtschaft. Bericht für das Bundesamt für Naturschutz, Bonn, 141 S.

EU (EUROPEAN UNION) (1992): Council Directive 92/43/EEC 1992 on the conservation of natural habitats and of wild fauna and flora.

FAUNA EUROPAEA WEB SERVICE (2007): Fauna Europaea version 1.3, Available online at http://www.faunaeur.org

FELKE, M., LANGENBRUCH, G.-A. (2005): Auswirkungen des Pollens von transgenem Bt-Mais auf ausgewählte Schmetterlingslarven. BfN-Skripten 157. 143 S.

FORBES, V.E., HOMMEN, U., THORBEK, P. et al.(2009): Ecological Models in Support of Regulatory Risk Assessments of Pesticides: Developing a Strategy for the Future. Integrated Environmental Assessment and Management 5(1): 167-172.

FRENCH, B.W., CHANDLER, L.D., ELLSBURY, M.M. et al. (2004): Ground beetle (Coleoptera: Carabidae) assemblages in a transgenic corn-soybean cropping system. Environ. Entomol. 33: 554-563.

GALIC, N., HOMMEN, U., BAVECO, J.M., VAN DEN BRINK, P.J. (2010): Potential application of population models in the European ecological risk assessment of chemicals II: Review of models and their potential to address environmental protection aims. Integrated Environmental Assessment and Management 6(3): 338-360.

GENTPFLEV (2008): Verordnung über die gute fachliche Praxis bei der Erzeugung gentechnisch veränderter Pflanzen. Gentechnik-Pflanzenerzeugungsverordnung, BGBl. I: 655.

GRAEF, F., ZÜGHART, W., BENZLER, A., BERHORN, F., SUKOPP, U. (2007): Monitoring genetically modified plants (GMP): Data harmonisation and coordination on multiple levels to ensure data quality and comparability. Journal for Consumer Protection and Food Safety 2, Supplement 1: 72-75.

GRIMM, V., ASHAUER, R., FORBES, V.E. et al. (2009): CREAM: a European project on mechanistic effect models for ecological risk assessment of chemicals. Environ Sci Poll Res 16(6): 614-617.

GROSSI-DE-SA, M.F., LUCENA, W., SOUZA, M.L. et al. (2006): Transgene expression and locus structure of Bt cotton - Methodologies for assessing Bt cotton in Brazil. In: Hilbeck, A., Andow, D.A., Fontes, E.M.G. (Hrsg.): Environmental risk assessment of genetically modified organisms. Oxfordshire (CABI Publishing).

GRUBER, H., PAUL, V., GUERTLER, P. et al. (2011): Fate of Cry1Ab Protein in Agricultural Systems under Slurry Management of Cows Fed Genetically Modified Maize (*Zea mays* L.) MON810: A Quantitative Assessment. Journal of Agricultural and Food Chemistry, Volume 59, 13: 7135-7144.

GRUBER, H., PAUL, V., GUERTLER, P. ET AL. (2012): Determination of insecticidal Cry1Ab protein in soil collected in the final growing seasons of a nine-year field trial of Bt-maize MON810, Transgenic Research, Volume 21, 1: 77-88.

HALSEY M.E., REMUND K.M., DAVIS C.A. et al. (2005): Isolation of maize from pollen-mediated gene flow by time and distance. Crop Science Society of America 45: 2172-2185.

HANSKI, I., CAMBEFORT, Y. (1991): Dung beetle ecology. Princeton University Press, Princeton. 481 p.

HAWES, C., HAUGHTON, A.J., OSBORNE, J.L. et al. (2003): Responses of plants and invertebrate trophic groups to contrasting herbicide regimes in the farm scale evaluations of genetically modified herbicide-tolerant crops. Philos. T. Roy Soc. B 358: 1899-1913.

HILBECK, A., JÄNSCH, S., MEIER, M., RÖMBKE, J. (2008): Analysis and validation of present ecotoxicological test methods and strategies for the risk assessment of genetically modified plants. BfN-Skripten 236. 287 S.(

HOFMANN, F., JANICKE, U., JANICKE, L. et al. (2008a): Modellrechnungen zur Ausbreitung von Maispollen unter Worst-Case-Annahmen mit Vergleich von Freilandmeßdaten. Kurzgutachten. http://www.bfn.de/0301_veroe.html.

HOFMANN, F., EPP, R., KALCHSCHMID, A. et al. (2008b): GVO-Pollenmonitoring zum Bt-Maisanbau im Bereich des NSG/FFH-Schutzgebietes Ruhlsdorfer Bruch. Umweltwiss. Schadst. Forsch. 20: 275-289.

HOLLAND, J.M., FRAMPTON, G.K., VAN DEN BRINK, P.J. (2002): Carabids as indicators within temperate arable farming systems: Implications from SCARAB and LINK Integrated Farming System Projects. 251-277. In J. M. HOLLAND (Hrsg.), The agroecology of carabid beetles. Andover, UK.

HOLTER, P. (1979): Effect of dung-beetles (Aphodius spp.) and earthworms on the disappearance of cattle dung. Oikos 32: 393-402.

ICOZ, I., STOTZKY, G. (2008): Cry3Bb1 protein from *Bacillus thuringiensis* in root exudates and bio-mass of transgenic corn does not persist in soil. Transgenic Res. 17: 609-620.

ICOZ, I., SAXENA, D., ANDOW, D.A., ZWAHLEN, C., STOTZKY, G. (2008): Microbial Populations and Enzyme Activities in Soil In Situ under Transgenic Corn Expressing Cry Proteins from Bacillus thuringiensis. J Environ Qual. 37: 647-662.

IFAG – INSTITUT FÜR ANGEWANDTE GEODÄSIE (Hrsg.) (1979): Karte der Bundesrepublik Deutschland 1:1.000.000 - Landschaften (Namen und Abgrenzungen). Frankfurt/Main (Selbstverlag).

ISO (INTERNATIONAL ORGANIZATION FOR STANDARDIZATION) (2008a): Soil quality — Guidance for the selection and application of methods for the assessment of bioavailability of contaminants in soil and soil materials. ISO 17402. Geneva, Switzerland.

ISO (INTERNATIONAL ORGANIZATION FOR STANDARDIZATION) (2008b): ISO 17512-1. Soil quality -- Avoidance test for determining the quality of soils and effects of chemicals on behaviour -- Part 1: Test with earthworms (*Eisenia fetida* and *Eisenia andrei*). Geneva, Switzerland.

ISO (INTERNATIONAL ORGANIZATION FOR STANDARDIZATION) (2010): ISO 17512-2. Soil quality -- Avoidance test for determining the quality of soils and effects of chemicals on behaviour -- Part 2: Test with collembolans (*Folsomia candida*). Geneva, Switzerland.

ISO (INTERNATIONAL ORGANIZATION FOR STANDARDIZATION) (2011): Soil quality – Guidance on the establishment and maintenance of monitoring programmes. ISO 16133. Geneva, Switzerland.

JAGER, T., ALBERT, C., PREUSS, T.G., ASHAUER, R. (2011): General Unified Threshold Model of Survival - a Toxicokinetic-Toxicodynamic Framework for Ecotoxicology. Environmental Science & Technology 45:2529-2540.

JÄNSCH, S., RÖMBKE, J., HILBECK, A., WEIß, G., TEICHMANN, H., TAPPESER, B. (2011): Assessing the potential risks of transgenic plants for non-target invertebrates in Europe: a review of classification approaches of the receiving environment. BioRisk, 6: 19-40.

LAVELLE, P., SPAIN, A.V. (2001): Soil Ecology. Kluwer Academic Publishers, Dordrecht, The Netherlands. 654 S.

LENNARTZ, G. (2003): Der biozönologische-soziologische Klassifikationsansatz und dessen Anwendung in der Naturschutzpraxis -dargestellt am Beispiel der Borstgrasrasen (Violion) der Eifel unter Berücksichtigung der Laufkäfer, Spinnen, Heuschrecken, Tagfalter und Schwebfliegen. Dissertation RWTH Aachen. Akademische Edition Umweltforschung. Aachen.

LOZZIA, G.C. (1999): Biodiversity and structure of ground beetle assemblages (Coleoptera: Carabidae) in Bt corn and its effects on nontarget insects. B. Zool. Agr. Bachicol. 31: 37-58.

LUDY, C., LANG, A. (2006): A 3-year field-scale monitoring of foliage-dwelling spiders (Araneae) in transgenic Bt maize fields and adjacent field margins. Biocontrol 38: 314- 324.

MARQUARD, E., DURKA, W. (2005): Auswirkungen des Anbaus gentechnisch veränderter Pflanzen auf Umwelt und Gesundheit: Potenzielle Schäden und Monitoring. UFZ-Umweltforschungszentrum Leipzig-Halle.

MCELROY, A.E., BARRON, M.G., BECKVAR, N. et al. (2010): A Review of the Tissue Residue Approach for Organic and Organometallic Compounds in Aquatic Organisms. Integrated Env. Assess. And Management 7/1. 50-74.

MESSÉAN, A., ANGEVIN, F., GOMEZ-BARBERO, M. et al. (2006): New case studies on the coexistence of GM and non-GM crops in European agriculture. European Commission, Joint Research Centre.

MEYNEN, E., SCHMIDTHÜSEN, J., GELLERT, J. et al. (Hrsg.) (1953-62): Handbuch der naturräumlichen Gliederung Deutschlands, Bd. 1-9. - Remagen, Bad Godesberg.

MIDDELHOFF, U., HILDEBRANDT, J., BRECKLING, B. (2006): Die Ökologische Flächenstichprobe als Instrument eines GVO-Monitoring. BfN-Skriptum 172. Ergebnisse des gleichnamigen F+E-Vorhabens im Auftrag des Bundesamtes für Naturschutz, BfN, Bonn.

NAIDU, R., SEMPLE, K.T., MEGHARAJ, M. et al. (2008): Bioavailability, definition, assessment and implications for risk assessment. In: NAIDU, R. et al (Eds.) Chemical bioavailability in terrestrial environment. Elsevier, Amsterdam, The Netherlands. pp 39-52.

NATAL-DA-LUZ, T., RÖMBKE, J., SOUSA, J.P. (2008): Avoidance tests in site-specific assessment – influence of soil properties on the avoidance response of Collembola and earthworms. Envir. Toxicol. Chem. 27: 1112-1117.

OECD (ORGANISATION FOR ECONOMIC CO-OPERATION AND DEVELOPMENT) (2010): Guidance document on the determination of the toxicity of a test chemical to the dung beetle *Aphodius constans*. Paris, France: OECD Environmental Health and Safety Publications No. 122. Series on testing and assessment.

PAULUS, R., RÖMBKE, J., RUF, A., BECK, L. (1999): A comparison of the litterbag-, minicontainer- and bait-lamina-methods in an ecotoxicological field experiment with diflubenzuron and btk. Pedobiologia 43: 120-133.

PEIJNENBURG, W., JENSEN, J., KULA, C. et al. (2012): Evaluation of exposure metrics for effect assessment of soil invertebrates. Critical Reviews in Environmental Science and Technology 42: 1862-1893.

PLACHTER, H., BERNOTAT, D., MÜSSNER, R. & RIECKEN, U. (2002): Entwicklung und Festlegung von Methodenstandards im Naturschutz. Schr.R. f. Landschaftspfl. u. Naturschutz 70: 566 S.

PREUSS, T.G., HOMMEN, U., ALIX, A. ET AL. (2009): Mechanistic effect models for ecological risk assessment of chemicals (MEMoRisk)-a new SETAC-Europe Advisory Group. Environ Sci Poll Res 16(3): 250-252.

PRIESNITZ, K. (2011): Potential impact of Diabrotica resistant Bt-maize expressing Cry3Bb1 on ground beetles (Coleoptera: Carabidae). Dissertation RWTH-Aachen.

PRIESTLEY, A.L., BROWNBRIDGE, M. (2008): Field trials to evaluate effects of Bt-transgenic silage corn expressing the Cry1Ab insecticidal toxin on non-target soil arthropods in northern New England, USA. Transgenic Res.

RIECKEN, U., FINCK, P., RATHS, U., SCHRÖDER, E., SSYMANK, A. (2003): Standard-Biotoptypenliste für Deutschland. 2. Fassung. Schr.R. f. Landschaftspfl. u. Natursch. 75: 66 S.

RÖMBKE, J. (2008): Bioavailability in soil: The role of invertebrate behaviour. In: NAIDU, R., BOLAN, N.S., MEGHARAJ, M. et al. (Eds.): Chemical Bioavailability in Terrestrial Environments. Developments in Soil Science 32. Elsevier Publishers, Amsterdam, The Netherlands. Pp. 245-260.

RÖMBKE, J., JÄNSCH, S., MEIER, M., HILBECK, A., TEICHMANN, H., TAPPESER, B. (2010): General recommendations for soil ecotoxicological tests suitable for the Environmental Risk Assessment (ERA) of Genetically Modified Plants (GMPs). IEAM 6: 287-300.

RÖMBKE, J., JÄNSCH, S., ROSS-NICKOLL, M. et al. (2012): Erfassung und Analyse des Bodenzustands im Hinblick auf die Umsetzung und Weiterentwicklung der Nationalen Biodiversitätsstrategie. UBA-Texte 33/2012, 386 S.

ROMEIS, J., BATTINI, M., BIGLER, F. (2003): Transgenic wheat with enhanced fungal resistance causes no effects on *Folsomia candida* (Collembola: Isotomidae). Pedobiologia 47: 141-147.

ROß-NICKOLL, M., LENNARTZ, F., FÜRSTE, A. et al. (2004): Die Arthropodenfauna von grasigen Feldrainen (off crop) und die Konsequenzen für die Bewertung der Auswirkungen von Pflanzenschutzmitteln auf den terrestrischen Bereich des Naturhaushaltes. UBA Texte Berlin.

RUF A., BEYLICH, A., BLICK, T. et al. (2013): Soil organisms as an essential element of a monitoring plan to identify the effects of GMO cultivation. Requirements – Methodology – Standardisation. BioRisk 8: 73–87. doi: 10.3897/biorisk.8.3255

SAXENA, D., STOTZKY, G. (2001a): *Bacillus thuringiensis* (Bt) toxin released from root exudates and biomass of Bt corn has no apparent effect on earthworms, nematodes, protozoa, bacteria and fungi in soil. - Soil Biology and Biochemistry 33: 1225-1230.

SAXENA, D., STOTZKY, G. (2001b): Bt corn has a higher lignin content than non-Bt corn. - American Journal of Botany 88: 1704-1706.

SCHÄFFER, A., VAN DEN BRINK, P.J., HEIMBACH, F. et al. (2010): Guidance from the SETAC Europe Workshop: Semi-field Methods for the Environmental Risk Assessment of Pesticides in Soil (PERAS). CRC Press, Boca Raton, USA. 105 pp.

SCHRÖDER, W., SCHMIDT, G. (2000): Raumgliederung für die Ökologische Umweltbeobachtung des Bundes und der Länder. Umweltwissenschaften und Schadstoff-Forschung - Zeitschrift für Umweltchemie und Ökotoxikologie 12: 237-243.

SEMPLE, K.T., DOICK, K.J., JONES, K.C., BURAUEL, P., CRAVEN, A., HARMS, H. (2004): Defining bioavailability and bioaccessibility of contaminated soil and sediment is complicated. Environ. Sci. Technol. 38: 228A–231A.

SQUIRE, G.R., BROOKS, D.R., BOHAN, D.A. et al. (2003): On the rationale and interpretation of the Farm Scale Evaluations of genetically modified herbicide-tolerant crops. Philosophical Transactions of the Royal Society B: Biological Sciences 358: 1779 –1799.

THEIßEN, B., RUSSELL, D.J. (2009): Zur Bedeutung von Collembolen im GVO-Monitoring. Gefahrstoffe – Reinhaltung der Luft 69: 391-394.

TOSCHKI, A., HOTHORN, L.A., ROß-NICKOLL, M. (2007): Effects of cultivation of genetically modified Bt maize on epigeic arthropods (Araneae; Carabidae). Environ. Entomol. 36: 966-980.

TOSCHKI, A. (2008): Eignung unterschiedlicher Monitoring-Methoden als Grundlage zum Risk Assessment für Agrarsysteme am Beispiel einer biozönologischen Reihenuntersuchung und einer Einzelfallstudie. Dissertation RWTH Aachen, Institut für Umweltforschung.

TURBÉ, A., DE TONI, A., BENITO, P. et al. (2010): Soil biodiversity: functions, threats, and tools for policy makers. BioIntelligence Service, IRD, and NIOO, Report for European Commission (DG Environment), Brussels, Belgium. 250 S.

UBA (UMWELTBUNDESAMT) (2011): http://www.umweltbundesamt.de/boden-und-altlasten/boden/bodenschutz/dauerbeobachtung.htm. Letzte Änderung: 29.12.2011.

VDI (VEREIN DEUTSCHER INEGNIEURE) (2012): Monitoring der Wirkungen von gentechnisch veränderten Organismen (GVO) – Wirkungen auf Bodenorganismen. VDI 4331 Blatt 1 (Entwurf), 48 S.

VERCESI, M.L., KROGH, P.H., HOLMSTRUP, M. (2006): Can *Bacillus thuringiensis* (Bt) corn residues and Bt-corn plants affect life-history traits in the earthworm *Aporrectodea caliginosa*? Applied Soil Ecology 32: 180-187.

WEIGMANN, G. (2006): Hornmilben (Oribatida). In: Dahl, Tierwelt Deutschlands 76., Keltern (Goecke & Evers). 520 S.

YU, L., BERRY, R. E. & CROFT, B. A. (1997): Effects of Bacillus thuringiensis toxins in transgenic cotton and potato on *Folsomia candida* (Collembola: Isotomidae) and *Oppia nitens* (Acari: Orbatidae). Journal of Economic Entomology 90: 113-118.

ZÜGHART, W., BRECKLING, B. (2003): Konzeptionelle Entwicklung eines Monitoring von Umweltwirkungen transgener Kulturpflanzen Teil 1. UBA Text Berlin.

ZÜGHART, W., BENZLER, A., BERHORN, F., SUKOPP, U., GRAEF, F. (2008): Determining indicators, methods and sites for monitoring potential adverse effects of genetically modified plants to the environment: the legal and conceptional framework for implementation. Euphytica 164: 845-852.

ZÜGHART, W., RAPS, A., WUST-SAUCY, A.-G., DOLEZEL, M., ECKERSTORFER, M. (2011): Monitoring of genetially modified organisms. A policy paper representing the view of the National Environment Agencies in Austria and Switzerland and the Federal Agency for Nature Conservation in Germany. Umweltbundesamt GmbH, Vienna, Austria, 56 p.

ZWAHLEN, C., HILBECK, A.P., NENTWIG, W. (2007): Field decomposition of transgenic Bt maize residue and the impact of non-target soil invertebrates. Plant & Soil 300: 245-257.

6 Zusammenfassung

Einleitung

Ziel dieses Vorhabens war die Prüfung der Frage, ob bzw. wie das durch die Bundesländer betriebene Programm der Boden-Dauerbeobachtung in das durch die Freisetzungsrichtlinie 2001/18/EG (EC 2001) verlangte Monitoring-Programm zur Überwachung der Umweltwirkungen gentechnisch veränderter Organismen (GVO) eingebunden werden kann (MIDDELHOF et al. 2006; ZÜGHART et al. 2011). Im Einzelnen ging es um die:

1. Erarbeitung von fachlichen Anforderungen an ein aussagefähiges und wissenschaftlich fundiertes Monitoring der Auswirkungen von GVO auf den Lebensraum Boden (speziell an Ackerstandorten) und insbesondere die Erstellung einer Übersicht über potenzielle Wirkungspfade beim Anbau von GVO auf den Boden mittels einer Literaturrecherche.
5. Fachliche Bewertung der Möglichkeiten und Grenzen der Nutzung der Boden-Dauerbeobachtungsflächen (BDF) der Länder für das GVO-Monitoring, inklusive einer Beschreibung des Stands der Methodenharmonisierung und Datenhaltung sowie der Identifikation von aussagefähigen Indikatoren.
6. Entwicklung konkreter Erweiterungs- oder Anpassungsmöglichkeiten der Boden-Dauerbeobachtung der Länder und/oder ergänzender Monitoringmodule für das GVO-Monitoring auf der Grundlage von Ergebnissen zum bisherigen bzw. potentiellen Anbau von MON810 in Deutschland.
7. Prüfung der Datenverfügbarkeit sowie von Möglichkeiten der Datenauswertung für das GVO-Monitoring sowie die Entwicklung eines Modells für den Datenfluss und die Datenhaltung sowie Erarbeitung eines Vorschlags für ein Kooperationsmodell.

Die Exposition von Bodenorganismen (Literaturrecherche)

Expositionspfade von GVOs

Die Definition sowie die Untersuchung von Expositionspfaden dienen dem Zweck, ob bzw. in welchem Ausmaß Bodenorganismen (einzelne Populationen einer Art oder ganze Organismengemeinschaften) während des GVO-Anbaus mit einer der nachfolgend aufgeführten Auswirkungen konfrontiert werden (ISO 2008a; PEIJNENBURG et al. 2012):

- den GVO-spezifischen Wirkstoffen (z.B. *Bacillus thuringiensis* Cry-Proteine) oder seinen Metaboliten;
- physiologisch GV-veränderten Pflanzenbestandteilen, z.B. dem geänderten Stärkegehalt;
- landwirtschaftlichen Maßnahmen, die ohne GVO-Anbau nicht im gleichen Ausmaß erfolgen würden (z.B. einer erhöhten Herbizidausbringung).

Die möglichen Expositionspfade können auf verschiedene Arten klassifiziert werden.

- Organismen-bezogener Ansatz (z.B. HILBECK et al. 2008): direkte Exposition gegenüber lebenden oder toten GVO-Materialien; diese Einschätzung beruht vor allem auf den Erfahrungen mit GV-Mais bzw. dem Bt-Toxin.

- GVO-bezogener Ansatz (ESCHENBACH & WINDHORST 2009): Exposition durch Transport von GVO-Material. Da die komplexen Expositionsbedingungen im Freiland bisher wenig untersucht wurden (SQUIRE et al. 2003), wurden theoretische Überlegungen einbezogen: Horizontaler Gentransfer (EFSA 2005), Auswilderung und Durchwuchs (ZÜGHART & BRECKLING 2003) sowie Pollenausbreitung (HALSEY et al. 2005).

Exposition von Boden-Dauerbeobachtungsflächen (BDF) gegenüber GV-Pflanzen (GVP)

BDF sind potentiell gegenüber GVP exponiert, was deren Nutzen in einem Monitoring-Programm entscheidend beeinflussen kann. Im diesem Vorhaben wurde nur der Pollenflug als Pfad mit möglichem erheblichem Einfluss berücksichtigt, da andere Pfade aufgrund der räumlich und zeitlich begrenzten Ausbringung sowie der starken Veränderungen (z.B. Abbau) des GVP-Materials in Dung oder Gülle als weniger relevant eingeschätzt werden. Hinsichtlich der Ausbreitung von Pollen variieren die Literaturangaben von wenigen Metern bis hin zu mehreren Kilometern (BIOSICHERHEIT 2010, HOFMANN et al. 2008a). In diesem Vorhaben wurden drei Distanzen (50, 150, 1000 m) als Ausbreitungs- und Wirkszenarien berücksichtigt.

Bodenorganismen, die für die Beobachtung von GVO-Wirkungen relevant sind

Bisher gibt es nur wenige Erfahrungen zur Erfassung der Wirkungen von GVOs im Freiland (CANDOLFI et al. 2004, CORTET et al. 2006, LUDY & LANG 2006, TOSCHKI et al. 2007, PRIESTLEY & BROWNBRIDGE 2008); siehe dazu auch die Übersicht von THEIßEN & RUSSELL (2009). Ausgehend von bis hierher skizzierten Erfahrungen und Überlegungen hat ein Fachausschuss des Vereins Deutscher Ingenieure (VDI) eine Richtlinie mit Vorschlägen für die Auswahl von Organismengruppen im Rahmen des GVO-Monitorings erarbeitet (VDI 2012; RUF et al. 2013). Um den Bodenzustand in fachlich angemessener Weise bewerten zu können, sollten für ein GVO-Monitoring Taxa unter Verwendung folgender Kriterien ausgewählt werden:

- Wichtige ökologische Funktion im Ökosystem, Repräsentanz für eine trophische Ebene;
- Enge Anbindung an den Mineralboden oder die Streuschicht;
- Ausreichend hohe Diversität (Artenvielfalt), um Standorte differenzieren zu können;
- Gute taxonomische und ökologische Kenntnisse;
- Weite Verbreitung in Mitteleuropa;
- Vorhandensein von standardisierten Erfassungsmethoden;
- Potenzial zum Routineeinsatz, z.B. in Hinsicht auf eine vereinfachte Determination;
- Verfügbarkeit von Daten aus bestehenden Monitoring-Systemen.

Es sollten mindestens vier Taxa verwendet werden, die es ermöglichen unterschiedliche trophische (epigäisch-endogäisch) sowie funktionelle Ebenen (Ernährungstyp) einzubeziehen.

Fachliche Bewertung der Möglichkeiten und Grenzen einer Nutzung der BDF der Länder für das GVO-Monitoring

Das Boden-Dauerbeobachtungsprogramm: Einleitung

Das Ziel der Boden-Dauerbeobachtung ist es, den aktuellen Zustand der Böden zu erfassen und langfristig zu überwachen (UBA 2011). Dafür gibt es 795 BDFs in Deutschland, die allerdings nicht gleichmäßig verteilt sind (siehe Abbildung).

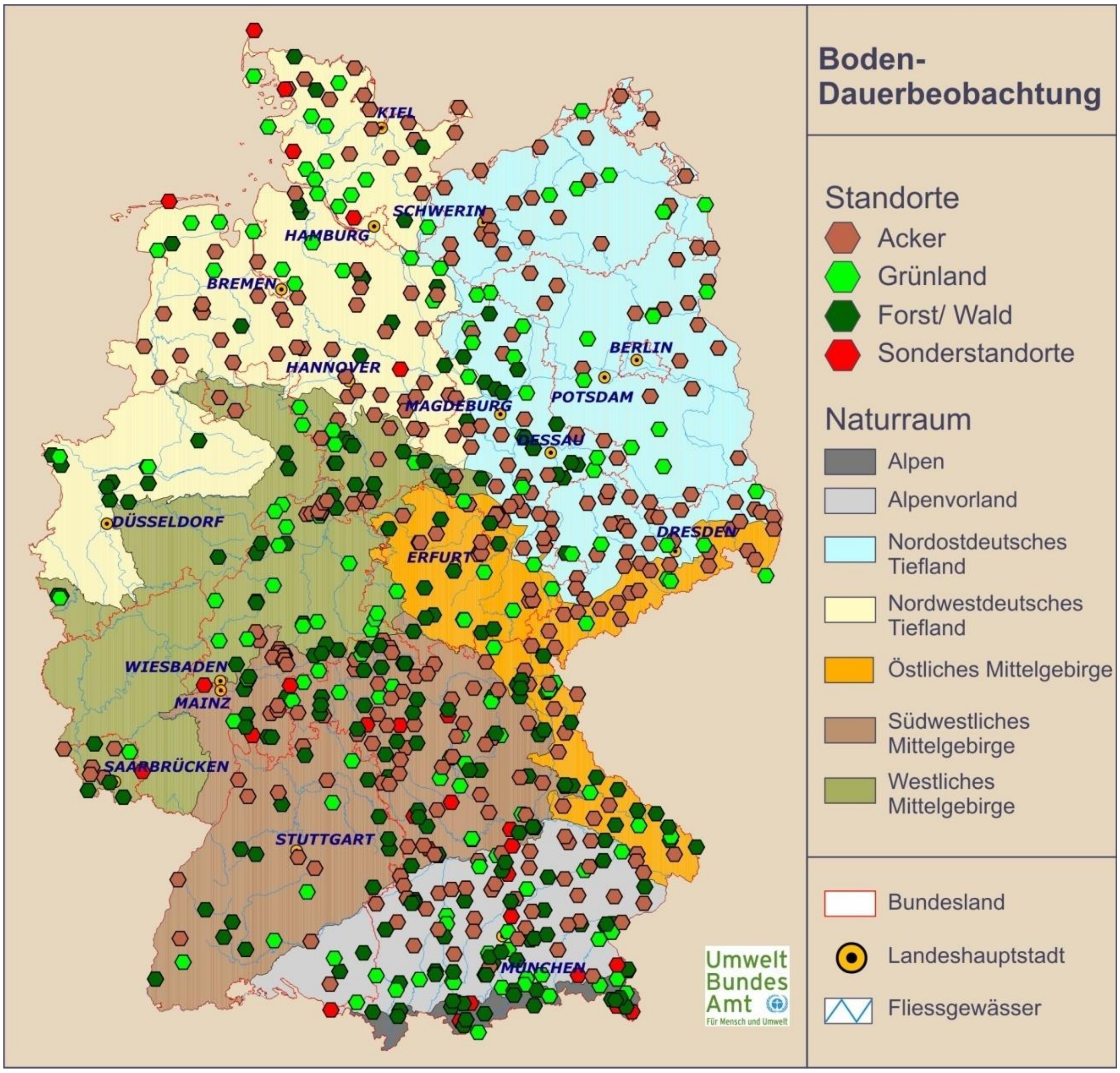

Boden-Dauerbeobachtungsflächen (BDF) in den verschiedenen deutschen Naturräumen, differenziert nach Landnutzung (RÖMBKE et al. 2012).

Bislang konzentriert sich die Boden-Dauerbeobachtung der Bundesländer auf physikalische und chemische Messgrößen, die dann im Rahmen der Bund-Länder-Verwaltungsvereinbarung über den Datenaustausch im Umweltbereich in der UBA-Datenbank „BDF – Bodendauerbeobachtungsflächen" zusammengeführt werden. Nur in wenigen Fällen werden standardisiert biologische Daten erhoben. Die Lage der BDF sowie deren jeweilige Eigenschaften (z.B. Biotoptyp, Nutzung, Bodenart etc.) sowie die

vorhandenen biologischen Daten wurden in der Datenbank Bo-Info zusammengestellt (RÖMBKE et al. 2012).

Repräsentativität der BDF

Um die Repräsentativität der BDF als Grundlage für ein Monitoring der Auswirkungen von GVO auf die Bodenlebensgemeinschaft einschätzen zu können wurden die Biotoptypen als Bewertungsgrundlage genutzt (RIECKEN et al. 2009). Biotoptypen differenzieren synökologisch unterschiedliche Boden-Lebensgemeinschaften und integrieren zusätzlich die relevanten Faktoren (speziell Bodentyp, Feuchte und Nährstoffversorgung), die für ihre Differenzierung ausschlaggebend sind (ROß-NICKOLL et al. 2004; TOSCHKI 2008). Bis dato werden im Wesentlichen nur drei Biotoptypen durch eine ausreichende Menge BDF-Standorte abgedeckt. Dies sind Äcker (351), Grünland (102) und Laubwälder (242).

Exposition von BDF

Um eine nachhaltige Nutzung des BDF-Programmes für das Monitoring von GVO zu gewährleisten sollte die Frage beantwortet werden, wie stark bestehende BDF in der Vergangenheit bereits gegenüber GVO exponiert waren bzw. in welchem Ausmaß eine Exposition bei einem zukünftigen Anbau zu erwarten ist. Als Beispiel-GVO wurde die insektenresistente Maissorte MON810 als bislang einzige in Deutschland auf größerer Fläche angebaute GVO ausgewählt, bei angenommenen Ausbreitungsradien (Expositionsdistanzen) für Pollen von 50, 150 und 1000 m. Diese Radien wurden in Anlehnung an unterschiedliche Abstandsregelungen in der EU zu konventionellen Anbauflächen (z.B. Spanien: 50 m, Deutschland: 150 m; GENTPFLEV 2008) sowie Empfehlungen aus Feldversuchen zur Maispollenausbreitung festgelegt.

Die folgenden Expositionsszenarien sind prinzipiell denkbar: 1) auf der BDF wurde der GVO angebaut; 2) die BDF lag nicht im Einflussbereich des GVO; 3) kein Anbau des GVO auf der BDF aber BDF lag im direkten Einflussbereich des GVO basierend auf den drei Pollen-Ausbreitungsradien. Diese Szenarien wurden beispielhaft für die Bundesländer Brandenburg, Hessen, Niedersachsen sowie Schleswig-Holstein untersucht. Die weitere Darstellung in dieser Zusammenfassung wird sich auf das Beispiel Brandenburg mit dem bislang stärksten MON 810 Anbau 2007 (ca. 1350 ha) konzentrieren.

Methodik

Für die vier ausgewählten Bundesländer konnten die InVeKoS (Integriertes Verwaltungs- und Kontrollsystem) Daten, mit denen landwirtschaftlich genutzte Parzellen GIS-basiert identifiziert werden, und die Koordinaten der Acker-BDF genutzt werden. Zur Frage, inwieweit die BDF gegenüber GVO exponiert waren, wurden die BDF-Standorte und MON810-Anbauflächen in einer gemeinsamen Karte dargestellt und die oben genannten drei Pollen-Ausbreitungsradien als Pufferabstände um die den MON810-Anbauflächen zugeordneten Feldblöcke gelegt. Das Ergebnis ist dabei mit einer Restunsicherheit behaftet, da nicht zu jeder MON810-Anbaufläche zweifelsfrei der dazu gehörige Feldblock identifiziert werden konnte. Als Beispiel ist in der folgenden Abbildung die Exposition des Feldblocks der Acker-BDF Gusow (blau) im Landkreis Märkisch-Oderland (Brandenburg) gegenüber MON810-Anbau (dunkelgrün) unter Annahme einer Pollenausbreitung von

50, 100 und 1000 m (rote Puffer) dargestellt (betrachteter Zeitraum: 2005 – 2008). Aus diesem und weiteren Beispielen geht hervor, dass bei einer möglicherweise künftigen Intensivierung des GVO-Anbaus in Deutschland weitere Acker-BDF gegenüber GVO exponiert sein werden.

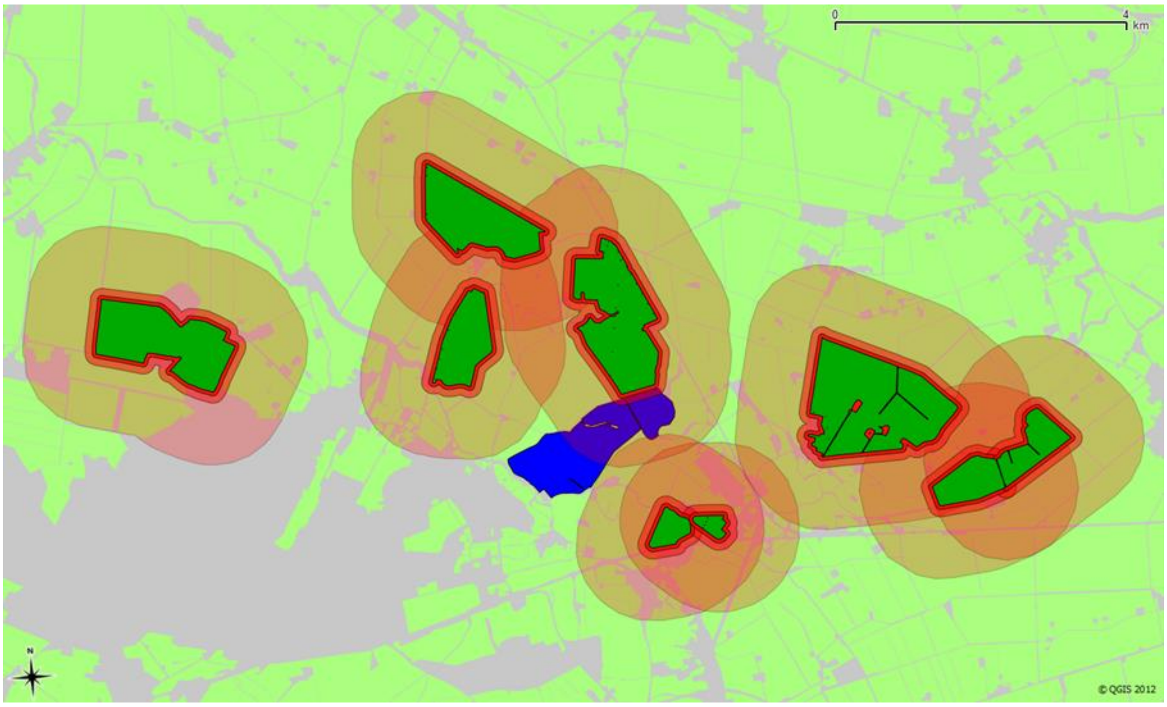

Exposition des Feldblocks der Acker-BDF Gusow (blau) im Landkreis Märkisch-Oderland (Brandenburg) gegenüber MON810-Anbau (dunkelgrün) unter Annahme einer Pollenausbreitung von 50, 100 und 1000 m (rote Puffer) 2005-2008.

Fazit: Repräsentativität der BDF, speziell in Hinsicht auf deren Nutzung für bodenbiologische Monitoringprogramme

Insgesamt kann nach derzeitigem Stand von einer guten Repräsentanz der BDF in Deutschland für Ackerstandorte ausgegangen werden. Dies bezieht sich jedoch lediglich auf den allgemein als Acker und Ackerbrachen bezogenen Basis-Biotoptyp. Für die Bodenorganismen sind aufgrund ihrer Lebensweise weitere standortspezifische Merkmale (Bodenparameter, Nährstoffversorgung, Feuchte etc.) für ihre Verteilung relevant, die sich in der tiefer gehenden Gliederung der Biotoptypen (2. und 3. Ebene) widerspiegeln. Dafür fehlen häufig genaue Angaben, z.B. zum Untergrund (Kalk, Silikat, Sand etc.) bzw. zur allgemeinen Nährstoffversorgung (extensiv artenreich, intensiv, nährstoffreich, artenarm, etc.). Eine standardisierte und möglichst genaue Datenerhebung dieser Parameter ist anzustreben, da die Verteilung von Bodentieren auf der untersten Klassifikationsebene die stärksten Korrelationen zeigt (vgl. TOSCHKI 2008, ROß-NICKOLL et al. 2004).

Die BDF-Datensätze enthalten wenig bodenbiologische Daten, und der Datenbestand weist hinsichtlich der Vollständigkeit der Daten pro Tiergruppe folgende gravierende Lücken auf:

- Lumbricidae (bestes Datenpaket): 97 BDF-Standorte (von 795), repräsentativ für Grünland, Äcker und Wälder, aber Zuordnung zu tieferen Ebenen der Biotoptypenliste nur in Ansätzen möglich, in einigen Bundesländern lückenhaft;
- Enchytraeidae: Daten aus 60 BDF vorhanden; regionale Repräsentativität ist aber nicht gegeben (z.B. wenige Daten aus Ostdeutschland, Bayern oder Rheinland-Pfalz;
- Collembola, Oribatida, sowie Bodeninvertebraten generell: keine Datensätze aus BDF
- Mikroben: bisher keine Daten, die zur Beurteilung von Diversität geeignet sind.

Zusammenfassend legen die bisherigen Ergebnisse nahe, dass:

- die Datenlage zum Vorkommen der wichtigsten Gruppen der Bodenorganismen auf den BDF nicht ausreichend ist, um diese für ein GVO-Monitoring zu nutzen;
- alle BDF unter Verwendung einer einheitlichen Biotoptypenliste einzustufen sind;
- weitere Probenahmen auf repräsentativ ausgesuchten BDF sind zu empfehlen;
- unter Verwendung neuer, speziell genetischer Determinationsmethoden (z.B. Barcoding), die taxonomische Bearbeitung vereinfacht und zugleich transparenter wird;
- die Grundlagen für ein Referenzsystem geschaffen werden müssen, um die beim GVO-Monitoring erhobenen Daten auch beurteilen zu können.

Entwicklung von Erweiterungs- oder Anpassungsmöglichkeiten der Boden-Dauerbeobachtung der Länder und / oder ergänzender Module für das GVO-Monitoring

In dem Versuch, den Mangel an bodenbiologischen Daten von BDF auszugleichen wurde auf die in der Datenbank Bo-Info gesammelten Angaben zurückgegriffen (Inhalt Stand heute: 1.744 Standorte mit über 42.000 Bodentier-Datensätzen, RÖMBKE et al. 2012).

Um ökologische Tier-Daten auf Landschaftsebene nutzbar zu machen, bedarf es eines einheitlichen Bezugsystems. Für eine Bewertung von Veränderungen im Rahmen des GVO-Monitoring erscheint es deshalb essentiell, standortbezogen sogenannte Referenzen (Soll-Werte, Summe der Referenzen = Referenzsystem) zu erstellen. Die Kenntnis dieser Referenzen erlaubt eine Bewertung des ökologischen Zustandes eines Standorts, die (weitgehend) unabhängig von der absoluten Diversität der dort vorkommenden Artengemeinschaft bzw. deren Individuendichte oder –anzahl ist. Erst durch die Kenntnis von biotopspezifischen Soll-Werten wird die Ableitung von Schwellenwerten, die erhebliche Veränderungen der Lebensgemeinschaft anzeigen, möglich (siehe Abbildung).

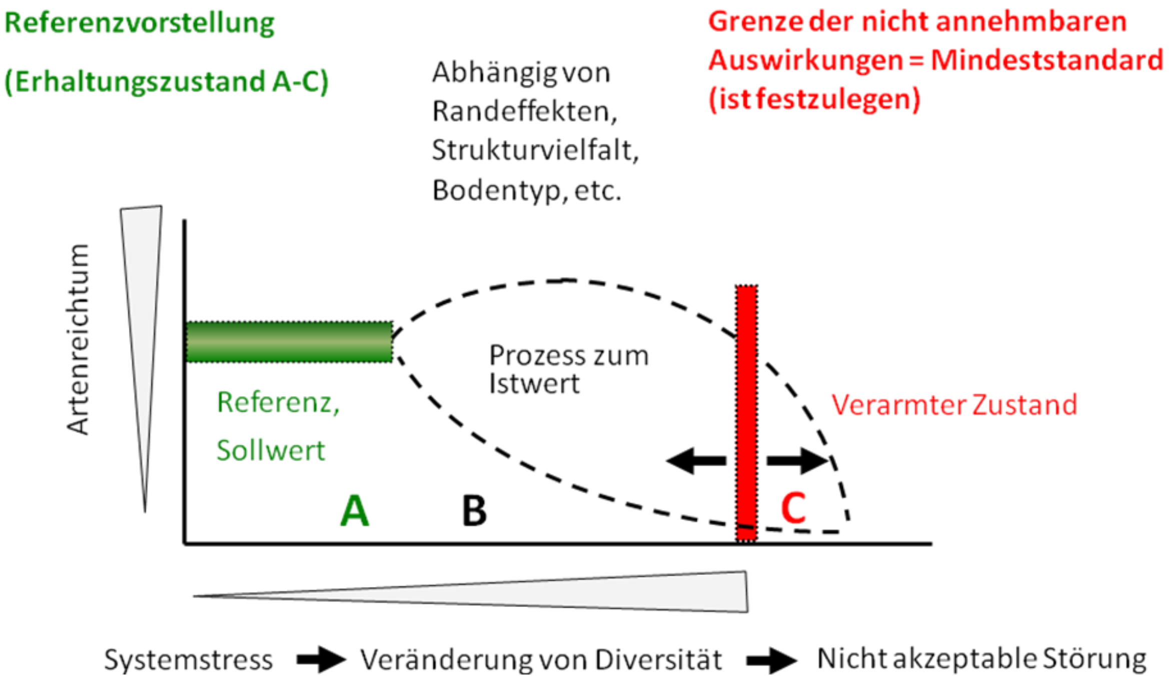

Ableitung von Schwellenwerten in Bezug auf Referenzzustände (RÖMBKE et al. 2012): A, B und C entsprechen verschiedenen Erhaltungszuständen in Bezug zum Systemstress (EU 1992)

Ein Referenzsystem zum Umgang mit der standortbezogenen Diversität von Bodenorganismen besteht dementsprechend aus:

- Listen von Arten und Spannen von Abundanzen, die an einem Standort mit spezifischen Bedingungen (z.B. Klima, Bodenfaktoren, Region usw.) auftreten sollten;
- einer Vorstellung, ab wann eine Abweichung von dieser Erwartung als negativ zu beurteilen ist.

Um eine basale räumlich-ökologische Bezugsgröße zur Bewertung von Effekten zu schaffen, ist eine entsprechende Typisierung der untersuchten Lebensräume unabdingbar. Die Nutzung der Biotoptypenliste für Deutschland (RIECKEN et al. 2009) stellt die Kompatibilität mit anderen Monitoringansätzen, des Naturschutz-Managements und voraussichtlich auch der Pestizidregistrierung sicher. In den Analysen der Organismengruppen zeigten sich klare Korrelationen zwischen dem Vorkommen von Arten und der jeweiligen hierarchischen Ebene im System der Biotoptypen-Liste (RÖMBKE et al. 2012). Die weitergehende Analyse zeigt, dass die Zusammensetzung von Gemeinschaften von den jeweiligen Standortfaktoren abhängig ist. Eine umfassende ökologische Beurteilung von Standorten erfordert die Integration verschiedener relevanter Organismengruppen auf Artebene, wodurch zugleich auch deren Funktionen (z.B. im Rahmen des Streuabbaus) abgedeckt werden.

Zusammenfassend lässt sich festhalten, dass der Ansatz, mögliche Wirkungen von anthropogenen Stressoren (inklusive GVOs) auf Organismengemeinschaften an ausgewählten Ackerstandorten zu erkennen, möglich ist. Dabei beruht die Beurteilung nicht auf dem direkten Vergleich zwischen einer bestimmten GVO-Fläche und einer benachbarten BDF, sondern auf einem Vergleich zwischen dieser GVO-Fläche und einer Referenz, die wie-

derum von (u.a.) BDF-Flächen abgeleitet wurde. Dieser Ansatz wird u.a. in Holland für die Beurteilung der Bodenqualität vorgeschlagen (BISQ), wobei strukturelle und funktionale Endpunkte eingesetzt werden (u.a. zu mehreren Organismengruppen) (RUTGERS et al. 2008).

Vorschläge zur Weiterentwicklung des Monitoring

Für die Etablierung eines GVO-Monitoring der Bodenbiodiversität ist es erforderlich, die Datengrundlage für eine Referenzwertentwicklung zu schaffen. Dies umfasst, neben der Erfassung der relevanten Biotoptypen, die Erhebung repräsentativer bodenbiologischer Daten. Aufgrund unserer Analyse kann gesagt werden, dass BDF dazu ein geeignetes Grundraster darstellen. Um den Einfluss von GVO auf die Bodenlebensgemeinschaft einzuschätzen ist die Entwicklung von Referenzen auf nicht von GVO beeinflussten Flächen notwendig. Sollen zu diesem Zwecke BDF genutzt werden, wäre es erforderlich, diese vor der Beeinflussung von GVO zu schützen.

Die im Folgenden aufgelisteten Empfehlungen gelten in weiten Teilen generell für das Monitoring und die Beurteilung der Bodenbiodiversität in Deutschland (vgl. RÖMBKE et al. 2012) und sind somit auch relevante Anforderungen an die Boden-Dauerbeobachtung bzw. das GVO-Monitoring. Empfehlungen für ein Minimalprogramm zum GVO-Monitoring von Bodenorganismen beinhalten im Wesentlichen fünf Aspekte:

1. Empfehlungen eines Mindestflächensets
2. Repräsentativität (z.B. des Biotoptyps, der Bundesländer, Naturräume etc.) eines Flächenrasters sowie die Einbindung weiterer Flächenspezifika, wie etwa der Flächen-Historie oder spezifischen Vorbelastungssituationen
3. Empfehlung eines Mindestdatensatzes zur Standortcharakterisierung, wobei alle Messungen nach ISO-Richtlinien (oder entsprechenden Vorgaben) durchzuführen sind (vgl. ISO (2011), TURBÉ et al. (2010), ENVIRONMENT CANADA (2012));
4. Empfehlungen eines Methodenstandards zum biologischen Monitoring generell, z.B. zur Organismenauswahl für eine ausreichende Darstellung des Schutzgutes Biodiversität sowie räumlich und zeitlich repräsentativen Erfassungsmethoden (VDI 2012)
5. Empfehlung eines Methodenstandards zum biologischen Monitoring auf BDF in Bezug auf die Nutzung im GVO Monitoring;
6. Empfohlene Merkmale eines abgestimmten Auswertungskonzeptes
7. Standards zur Datenhaltung und Möglichmachung von standardisierten Auswertungen.

Bezüglich der Datenauswertung bedarf der Begriff der „schädlichen Bodenveränderung" im Kontext des GVO-Monitoring zunächst der Erläuterung und Konkretisierung. Bislang gibt es keine Vorstellung von Lebensraum spezifischen Referenzwerten für einzelne Organismen bzw. Lebensgemeinschaften. Diese gilt es anhand des oben skizzierten Monitoring-Konzepts zu entwickeln. Eine Abweichung zu diesen Referenzen muss dann anhand von vorab zu definieren Schwellen beurteilt werden. Es gibt bislang keine Regelungen bezüglich Effekten auf die Bodenbiozönose im BBodSchG oder anderen Gesetzen,

sondern nur Vorsorge-, Prüf- und Maßnahmenwerte für stoffliche Parameter (d.h. bestimmte Konzentrationen einzelner Chemikalien dürfen in einem Boden bestimmter Nutzung nicht überschritten werden (BBodSchV 1999)).

Prüfung der Datenverfügbarkeit sowie von Möglichkeiten der Datenauswertung für das GVO-Monitoring

Die BDFs und damit die auf ihnen erhobenen Daten unterliegen der Hoheit der Bundesländer. Die fachliche Koordinierung erfolgt im Rahmen der Bund-Länder-Arbeitsgruppe Bodenschutz (LABO, https://www.labo-deutschland.de/Kontakt.html). Es gilt zunächst auf Bund-Länder-Ebene die Frage zu beantworten, ob die oben geforderte Einrichtung von Schutzzonen (kein GVO-Anbau auf und im Umfeld von BDF) mit dem eigentlichen BDF-

Konzept vereinbar ist. Die Betrachtung der Bodenbiologie und die Ableitung von Referenzwerten zur Beurteilung eventueller Effekte durch GVO als konkrete Belastungsquelle stellen allerdings eine neue Fragestellung für die BDF dar.

Eine Nutzbarkeit der Daten für ein bundesweites GVO-Monitoring erfordert eine standardisierte, für alle Länder gleiche bzw. vergleichbare Datenerhebung und Datenführung, so dass Ergebnisse zentral (z.B. naturräumlich, kulturabhängig) ausgewertet werden können (Plachter et al. 2002; Toschki 2008; Züghart et al. 2008). Hierbei ist zu beachten, dass:

1. die verschiedenen Länder-Programme derzeit unterschiedliche Strukturen hinsichtlich der Datenerhebungen, Datenhaltung/Datenverfügbarkeit und Datenauswertung besitzen;

2. die Daten erst über langfristige, vergleichbare Erhebungen Wert bekommen. Wichtig ist daher eine Kontinuität bei der Datenhaltung zu schaffen, was durch eine koordinierende zentrale Stelle (am besten an einer langfristig bestehenden Institution, z.B. einem Museum oder einem staatlichen Forschungsinstitut) gewährleistet werden kann,

3. einheitliche und vergleichbare Daten, die für die Bewertung in einem GVO-Monitoring notwendig sind, zu analysieren und zu diskutieren;

4. Mindeststandards für die Datenweitergabe und -haltung vorgegeben werden müssen.

Hinsichtlich der eigentlichen Durchführung einer länderübergreifenden Datenauswertung ist die Benennung kompetenter und unabhängiger Gremien erforderlich. Hierbei könnte die bewertende Behörde z.B. auf die bereits bestehende Arbeitsgruppe zur Auswertung der BDF-Daten, bestehend aus Vertretern von Bundes- und Landesbehörden, zurückgreifen. Für länderübergreifende Auswertungen ist das Umweltbundesamt (UBA) Ansprechpartner (Fachgebiet II 2.7 "Bodenzustand, Bodenmonitoring"; Dr. Frank Glante, Wörlitzer Platz 1, D06844 Dessau-Roßlau; Tel: 0340 2103 3434; e-mail: frank.glante@uba.de; http://www.umweltbundesamt.de/boden-und-altlasten/index.htm).

Im Auswertungskonzept sollten auch andere Nutzungsoptionen wie z.B. für die Risikobewertung von Pestiziden, die gleiche oder ähnliche Schutzziele verfolgen, in die Betrachtung eingebunden werden. Eine sinnvolle Verknüpfung von prospektiver und retrospektiver Risikobewertung sollte dabei angestrebt werden, um auch zukünftige Optionen entsprechend ihres Potentials anzusprechen. Eine wesentliche Rolle wird dabei aller Vo-

raussicht nach die zunehmende Verwendung von computergestützten Modellen zur Risikoanalyse spielen (EFSA 2010, GALIC et al. 2010, GRIMM et al. 2009, PREUSS et al. 2009).

Literatur

BBODSCHV (1999): Bundes-Bodenschutz- und Altlastenverordnung. Verordnung zur Durchführung des Bundes-Bodenschutzgesetzes. BGBl I, 36, S. 1554 – 1582 vom 16.06.1999.

BIOSICHERHEIT (2010): Coexistence of genetically modified and nongenetically modified maize: Making the point on scientific evidence and commercial experience. http://www.pgeconomics.co.uk/pdf/Co-existence_maize_10october2006.pdf, (28.05.2010).

CANDOLFI, M. P., BROWN, K., GRIMM, C., REBER, B., SCHMIDLI, H. (2004): A faunistic approach to assess potential side effects of genetically modified Bt-corn on nontarget athropods under field conditions. Biocontrol Sci. Techn. 14: 129-170.

CHAMBERLAIN, P.M., BULL, I.D., BLACK, H.J., INESON, P., EVERSHED, R.P. (2006): Collembolan trophic preferences determined using fatty acid distributions and compound-specific stable carbon isotope values. Soil Biol. Biochemistry 38: 1275-1281.

CORTET, J., ANDERSEN, M.N., CAUL, S. et. al (2006): Decomposition processes under Bt (Bacillus thuringiensis) maize: Results of a multi-site experiment. Soil Biology and Biochemistry 38: 195-199.

EC (EUROPEAN COMMISSION). (2001): EU Directive 2001/18/EC of 12 March 2001 on the deliberate release into the environment of genetically modified organisms and repealing Council Directive 90/220/EC. Official Journal of the European Communities L 106/1-38 (17.4.2001).

ENVIRONMENT CANADA (2012): Guidance Document on the Sampling and Preparation of Contaminated Soil for Use in Biological Testing. EPS 1/RM/53, 222 p.

EFSA (EUROPEAN FOOD SAFETY AUTHORITY) (2005): Guidance document of the scientific panel on genetically modified organisms for the risk assessment of genetically modified plants and derived food and feed. The EFSA Journal 99: 1-94.

EFSA (EUROPEAN FOOD SAFETY AUTHORITY) (2010): Scientific Opinion on the development of specific protection goal options for environmental risk assessment of pesticides, in particular in relation to the revision of the Guidance Documents on Aquatic and Terrestrial Ecotoxicology (SANCO/3268/2001 and SANCO/10329/2002). EFSA Journal. (PPR). 8(10).

ESCHENBACH, C., WINDHORST, W. (2009): Indikatoren für die nationale Strategie zur biologischen Vielfalt: Gentechnik in der Landwirtschaft. Bericht für das Bundesamt für Naturschutz, Bonn, 141 S.

EU (EUROPEAN UNION) (1992): Council Directive 92/43/EEC 1992 on the conservation of natural habitats and of wild fauna and flora.

GALIC, N., HOMMEN, U., BAVECO, J.M., VAN DEN BRINK, P.J. (2010): Potential application of population models in the European ecological risk assessment of chemicals II: Review of models and their potential to address environmental protection aims. Integrated Environmental Assessment and Management 6: 338-360.

GENTPFLEV (2008): Verordnung über die gute fachliche Praxis bei der Erzeugung gentechnisch veränderter Pflanzen. Gentechnik-Pflanzenerzeugungsverordnung, BGBl. I: 655.

GRIMM, V., ASHAUER, R., FORBES, V.E. et. al. (2009): CREAM: a European project on mechanistic effect models for ecological risk assessment of chemicals. Environ Sci Poll Res 16(6): 614-617.

HALSEY M.E., REMUND K.M., DAVIS C.A. et. al. (2005): Isolation of maize from pollen-mediated gene flow by time and distance. Crop Science Society of America 45: 2172-2185.

HILBECK, A., JÄNSCH, S., MEIER, M., RÖMBKE, J. (2008): Analysis and validation of present ecotoxicological test methods and strategies for the risk assessment of genetically modified plants. BfN-Skripten 236. 287 S.

HOFMANN, F., JANICKE, U., JANICKE, L., WACHTER, R., KUHN, U. (2008a): Modellrechnungen zur Ausbreitung von Maispollen unter Worst-Case-Annahmen mit Vergleich von Freilandmeßdaten. Kurzgutachten. http://www.bfn.de/0301_veroe.html.

ISO (INTERNATIONAL ORGANIZATION FOR STANDARDIZATION) (2008a): Soil quality — Guidance for the selection and application of methods for the assessment of bioavailability of contaminants in soil and soil materials. ISO 17402. Geneva, Switzerland.

ISO (INTERNATIONAL ORGANIZATION FOR STANDARDIZATION) (2011): Soil quality – Guidance on the establishment and maintenance of monitoring programmes. ISO 16133. Geneva, Switzerland.

LUDY, C., LANG, A. (2006): A 3-year field-scale monitoring of foliage-dwelling spiders (Araneae) in transgenic Bt maize fields and adjacent field margins. Biocontrol 38: 314- 324.

MIDDELHOFF, U., HILDEBRANDT, J., BRECKLING, B. (2006): Die Ökologische Flächenstichprobe als Instrument eines GVO-Monitoring. BfN-Skriptum 172. Ergebnisse des gleichnamigen F+E-Vorhabens im Auftrag des Bundesamtes für Naturschutz, BfN, Bonn.

PEIJNENBURG, W., JENSEN, J., KULA, C. et. al. (2012): Evaluation of exposure metrics for effect assessment of soil invertebrates. Critical Reviews in Environmental Science and Technology 42: 1862-1893. .

PLACHTER, H., BERNOTAT, D., MÜSSNER, R. & RIECKEN, U. (2002): Entwicklung und Festlegung von Methodenstandards im Naturschutz. Schr.Reihe f. Landschaftspflege u. Naturschutz 70: 566 S.

PREUSS, T.G., HOMMEN, U., ALIX, A. et. al. (2009): Mechanistic effect models for ecological risk assessment of chemicals (MEMoRisk)-a new SETAC-Europe Advisory Group. Environ Sci Poll Res 16(3): 250-252.

PRIESTLEY, A.L., BROWNBRIDGE, M. (2008): Field trials to evaluate effects of Bt-transgenic silage corn expressing the Cry1Ab insecticidal toxin on non-target soil arthropods in northern New England, USA. Transgenic Res.

RIECKEN, U., P. FINCK, U. RATHS, E. SCHRÖDER & A. SSYMANK (2009): German Red Data Book on endangered habitats (short version. July 2009). German Federal Agency for Nature Protection. Bonn-Bad Godesberg. Germany.

RÖMBKE, J., JÄNSCH, S., ROSS-NICKOLL, M. et.al. (2012): Erfassung und Analyse des Bodenzustands im Hinblick auf die Umsetzung und Weiterentwicklung der Nationalen Biodiversitätsstrategie. UBA-Texte 33/2012, 386 S.

ROß-NICKOLL, M., LENNARTZ, F., FÜRSTE, A. et. al (2004): Die Arthropodenfauna von grasigen Feldrainen (off crop) und die Konsequenzen für die Bewertung der Auswirkungen von Pflanzenschutzmitteln auf den terrestrischen Bereich des Naturhaushaltes. UBA-Texte Berlin.

RUF A., BEYLICH, A., BLICK, T. et. al. (2013): Soil organisms as an essential element of a monitoring plan to identify the effects of GMO cultivation. Requirements – Methodology – Standardisation. BioRisk 8: 73–87. doi: 10.3897/biorisk.8.3255

RUTGERS, M., MULDER, C., SCHOUTEN, A.J. et. al (2008): Soil ecosystem profiling in the Netherlands with ten references for biological soil quality. - RIVM-Report 607604009. 85 pp.

SQUIRE, G.R., BROOKS, D.R., BOHAN, D.A. et. al(2003): On the rationale and interpretation of the Farm Scale Evaluations of genetically modified herbicide-tolerant crops. Philosophical Transactions of the Royal Society B: Biological Sciences 358: 1779 –1799.

THEIßEN, B., RUSSELL, D.J. (2009): Zur Bedeutung von Collembolen im GVO-Monitoring. Gefahrstoffe – Reinhaltung der Luft 69: 391-394.

TOSCHKI, A., HOTHORN, L.A., ROß-NICKOLL, M. (2007): Effects of cultivation of genetically modified Bt maize on epigeic arthropods (Araneae; Carabidae). Environ. Entomol. 36: 966-980.

TOSCHKI, A. (2008): Eignung unterschiedlicher Monitoring-Methoden als Grundlage zum Risk Assessment für Agrarsysteme am Beispiel einer biozönologischen Reihenuntersuchung und einer Einzelfallstudie. Dissertation RWTH Aachen, Institut für Umweltforschung.

TURBÉ, A., DE TONI, A., BENITO, P. et. al (2010): Soil biodiversity: functions, threats, and tools for policy makers. BioIntelligence Service, IRD, and NIOO, Report for European Commission (DG Environment), Brussels, Belgium. 250 S.

UBA (UMWELTBUNDESAMT) (2011): http://www.umweltbundesamt.de/boden-und-altlasten/boden/bodenschutz/dauerbeobachtung.htm. Letzte Änderung: 29.12.2011.

VDI (VEREIN DEUTSCHER INGENIEURE) (2012): Monitoring der Wirkungen von gentechnisch veränderten Organismen (GVO) – Wirkungen auf Bodenorganismen. VDI 4331 Blatt 1 (Entwurf), 48 S.

ZÜGHART, W., BRECKLING, B. (2003): Konzeptionelle Entwicklung eines Monitoring von Umweltwirkungen transgener Kulturpflanzen Teil 1. UBA-Texte Berlin.

ZÜGHART, W., RAPS, A., WUST-SAUCY, A.-G., DOLEZEL, M., ECKERSTORFER, M. (2011): Monitoring of genetially modified organisms. A policy paper representing the view of the National Environment Agencies in Austria and Switzerland and the Federal Agency for Nature Conservation in Germany. Umweltbundesamt GmbH, Vienna, Austria, 56 p.

7 English Summary

Possibilities of using the German diurnal butterfly monitoring program (TMD) and the permanent soil monitoring program of the German federal states for the monitoring of GMO

Subproject II: Permanent soil monitoring of the federal states

UFOPLAN 2010: R&D 3509-89-0100 (ECT Project-No. P10035)

Sponsor: Federal Agency for Nature Conservation

Project advisor: Dr. Wiebke Züghart

Contractor: Consortium consisting of:

ECT Oekotoxikologie GmbH (coordination), Böttgerstr. 2-14, D-65439 Flörsheim

Dr. Jörg Römbke**,** Dr. Stephan Jänsch

RWTH Aachen University, Institute for Environmental Research, Worringer Weg 1, D-52074 Aachen

Dr. Martina Roß-Nickoll

gaiac Research institute for ecosystem analysis and assessment, Kackerstr. 10, D-52072 Aachen

Dr. Andreas Toschki

Introduction

The overall aim of this project was to assess the question whether it is possible to embed the permanent soil monitoring program of the German federal states into the monitoring program required by Directive 2001/18/EC on the deliberate release into the environment of genetically modified organisms (GMO; MIDDELHOF et al. 2006; ZÜGHART et al. 2011). The individual aims were:

1. Formulation of technical requirements of a meaningful and scientifically sound monitoring of the effects of GMO on the soil habitat (especially at agricultural sites) and in particular the compilation of potential effect pathways to soil through GMO cultivation by means of a literature research.
2. Technical evaluation of possibilities and limitations of the use of permanent soil monitoring sites (Boden-Dauerbeobachtungflächen; BDF) of the German federal states for GMO-monitoring including a description of the status of method harmonization and data management as well as identification of meaningful indicators.
3. Formulation of the possibilities to expand or adapt the federal permanent soil monitoring and/or complementary monitoring modules for the GMO-monitoring based on results of previous or potential cultivation of MON810 in Germany.
4. Evaluation of data availability and possibilities of data analysis for the GMO-monitoring, development of a model for data flow and management and formulation of a proposal for a cooperation model.

The exposure of soil organisms (literature research)

Exposure pathways of GMO

The definition and assessment of exposure pathways serve the purpose to estimate, whether or to what extent soil organisms (individual populations of a single species or whole communities) are confronted with any of the following impacts during GMO cultivation (ISO 2008a; PEIJNENBURG et al. 2012):

- GMO-specific active substances (e.g., *Bacillus thuringiensis* Cry proteins) or their metabolites;
- physiologically altered GM plant components (e.g., a modified starch content);
- agricultural management practices that wouldn’t be performed to the same extent without GMO cultivation (e.g., an increased application of herbicides).

The possible exposure pathways can be classified in various ways:

- organism-related approach (e.g., HILBECK et al. 2008): direct exposure towards living or dead GMO materials; this classification is mainly based on experience with GM maize and the Bt toxin.
- GMO-related approach (ESCHENBACH & WINDHORST 2009): exposure through transport of GMO materials. Since the complex exposure conditions in the field have rarely been assessed so far (SQUIRE et al. 2003), theoretical considerations were in-

cluded: horizontal gene transfer (EFSA 2005), unintentional release and second growth (ZÜGHART & BRECKLING 2003) as well as pollen dispersal (HALSEY et al. 2005).

Exposure of BDF towards GMO

BDF are potentially exposed towards GMO, possibly influencing their usability within a monitoring program. Within this project only pollen dispersal was considered as a pathway with a profound impact potential while other pathways were regarded as being less relevant due to spatially and temporarily limited GMO release and the strong alteration (e.g., decomposition) of GMO material in dung or manure. Literature data regarding the dispersal of pollen vary from a few meters to several kilometres (BIOSICHERHEIT 2010, HOFMANN et al. 2008a). In this project three distances (50, 150, 1000 m) were considered as dispersal and effect scenarios.

Soil organisms relevant to the monitoring of GMO related effects

Up to now there is little experience regarding the assessment of GMO related effects in the field (CANDOLFI et al. 2004, CORTET et al. 2006, LUDY & LANG 2006, TOSCHKI et al. 2007, PRIESTLEY & BROWNBRIDGE 2008); see also the overview compiled by THEIßEN & RUSSELL (2009). Based on the experience and considerations presented so far a technical committee of the Association of German Engineers (Verein Deutscher Ingenieure; VDI) developed a guideline with proposals for choosing organism groups within the scope of GMO-monitoring (VDI 2012; RUF et al. 2013). In order to assess the biological soil quality in technically appropriate fashion organism taxa should be selected according to the following criteria:

- important ecological function within the ecosystem, representativeness for a trophic level;
- close association with the mineral soil or the litter layer;
- - sufficient species diversity to differentiate between sites;
- good taxonomical and ecological knowledge;
- wide distribution in Central Europe;
- existing standardized sampling methods;
- potential for routine use, e.g. regarding simplified determination methods;
- availability of data from existing monitoring programs.

At least four different taxa should be used that facilitate the inclusion of different trophic (epigeic-endogeic) as well as functional (feeding type) levels.

Technical evaluation of possibilities and limitations of the use of BDF of the German federal states for GMO-monitoring

The German permanent soil monitoring program: Introduction

The aim of the German permanent soil monitoring program is to assess the current state of soils and its monitoring in the long term (UBA 2011). To this end, there are 795 German BDF that are, however, unevenly distributed (see below figure)

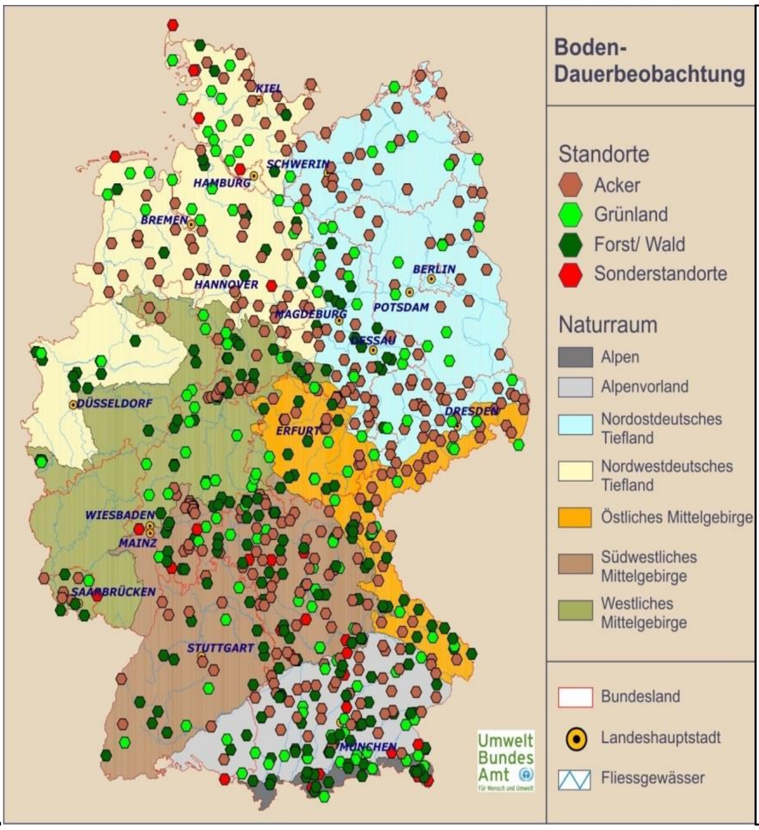

Permanent soil monitoring

Sites:

- Crop sites
- Grassland
- Forests
- Specific sites

Natural region:

- Alps
- Alpine foothills
- North-eastern lowlands
- North-western lowlands
- Eastern highlands
- South-western highlands
- Western highlands

Federal state

Capital of a federal state

Rivers

Currently, the permanent soil monitoring program of the German federal states focuses on physical and chemical measurements that are transferred into the database „BDF – Bodendauerbeobachtungsflächen“ at the German Federal Environmental Agency within the scope of the administrative agreement of federal and state agencies regarding data exchange. In only a few cases standardized biological data are collected. The position of the BDF, their properties (e.g., biotope type, land use, texture, etc.) and available biological data were compiled in the database Bo-Info (RÖMBKE et al. 2012).

Representativeness of BDF

In order to assess the representativeness of BDF for a monitoring of effects of GMO on the soil biocoenosis the classification of biotope types according to RIECKEN et al. (2009) was used as a basis of valuation. Biotope types harbour synecologically different soil animal communities and additionally integrate the factors (in particular soil type, moisture and nutrient supply) relevant for this differentiation of communities (ROß-NICKOLL et al. 2004; TOSCHKI 2008). To date, mainly only three biotope types are represented by a sufficient number of BDF: arable land (351), grassland (102) and deciduous forests (242).

Exposure of BDF

In order to ensure a sustainable use of the BDF program for the monitoring of GMO the question had to be answered to what extent existing BDF had already been exposed towards GMO and what intensity of exposure may be expected from future cultivation of GMO. The insect resistant maize variety MON810 was chosen as an example GMO (the only GMO cultured on a broader scale in Germany so far) and assuming pollen dispersal radii of 50, 150 and 1000 m. These distances were chosen in dependence on different

European buffer zone regulations towards conventional fields (e.g., Spain: 50 m, Germany: 150 m; GENTPFLEV 2008) and recommendations from field trials on maize pollen dispersal.

The following exposure scenarios are generally possible: 1) GMO cultivation on the BDF; 2) the BDF located outside the area of GMO influence; 3) no GMO cultivation on the BDF itself but BDF within the area of direct influence of the GMO based on the three pollen dispersal radii. These scenarios were exemplarily investigated for the federal states of Brandenburg, Hesse, Lower Saxony and Schleswig-Holstein. The further presentation in this summary will focus on the example of Brandenburg where the most intense MON810 cultivation in Germany occurred so far (ca. 1,350 ha in 2007).

Methodology

For the four exemplary federal states the InVeKoS (integrated administration and control system; Integriertes Verwaltungs- und Kontrollsystem) data that identify (GIS based) agriculturally managed lots of land, and the coordinates of BDF on arable land could be used. The BDF sites and the area under MON810 cultivation were plotted in a common map to answer the question to what extent BDF had already been exposed towards MON810. The above mentioned pollen dispersal radii were projected as buffer zones around the lots of land under MON810 cultivation. The result has a residual uncertainty since not every lot of land under MON810 cultivation could be exactly identified beyond doubt. The exposure of the lot of land containing the BDF "Gusow" (blue) within the administrative district of "Märkisch-Oderland" (Brandenburg) towards MON810 cultivation (2005-2008; dark green) assuming a pollen dispersal of 50, 100 and 1000 m (reddish buffer) is depicted below as an example. From this and other examples it became clear that with an intensification of GMO cultivation in Germany more BDF on arable land will likely become exposed towards GMO.

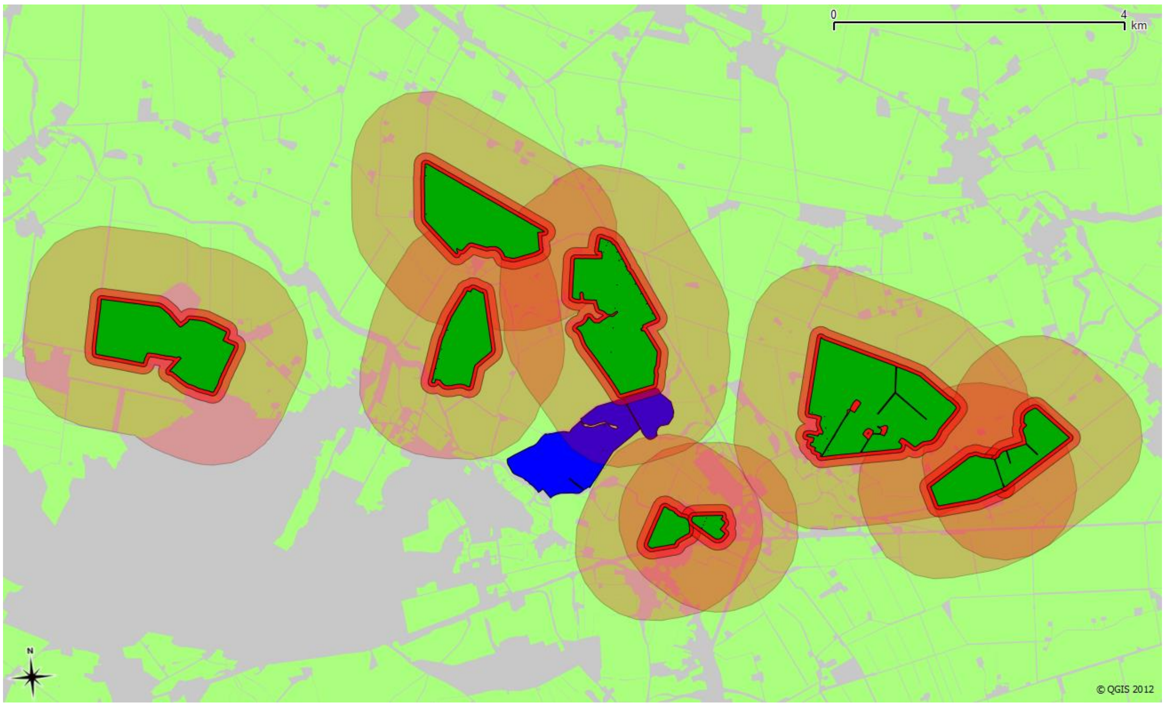

Conclusion: Representativeness of BDF, especially regarding their use for soil biological monitoring programs

There is a generally good representativeness of German BDF for arable land. However, this statement is limited to the basic biotope type "arable and fallow land" (RIECKEN et al. 2009). Due to their life-form, for soil organisms additional site specific parameters (soil properties, nutrient supply, moisture, etc.) are relevant for their distribution. These parameters are reflected in the further subdivision of biotope types (2nd and 3rd level). For an allocation of BDF to certain biotope types often detailed data are missing, e.g. regarding bedrock (lime, silicate, sand, etc.) or general nutrient availability (extensive species-rich, intensive, nutrient-rich, species-poor, etc). A standardized and detailed data collection should be pursued since the distribution of soil animals shows the strongest correlation at lower levels of site classification (see also TOSCHKI 2008, ROß-NICKOLL et al. 2004).

The data sets originating from BDF contain little soil biological data and the data inventory fundamentally lacks comprehensive data for most organism groups:

- Lumbricidae (best data packet): 97 BDF sites (of 795), representative for grassland, agricultural sites and forests but allocation of BDF to further levels of biotope types only rudimentarily possible, gaps for some federal states;
- Enchytraeidae: data from 60 BDF sites; no regional representativeness (e.g., little data from Eastern Germany, Bavaria or Rhineland-Palatinate);
- Collembola, Oribatida, and other soil invertebrates in general: no data sets from BDF;
- Microbes: thus far no suitable data for biodiversity assessment.

In summary, the results so far indicate that:

- the data basis on the occurrence of the most important soil organism groups is not sufficient to be used for a monitoring of GMO;
- all BDF need to be classified according to a standardized list of biotope types;
- additional sampling on representative BDF is recommended;
- the taxonomical determination of soil organisms needs to become simpler and more transparent by using novel (especially genetic) determination methods (e.g., DNA-barcoding);
- the basis for a reference system needs to be created in order to be able to evaluate the data generated within the monitoring of GMO.

Formulation of possibilities to expand or adapt the federal permanent soil monitoring and/or complementary monitoring modules for the GMO-monitoring

In the attempt to tackle the lack of soil biological data from BDF sites the data collected in the database Bo-Info were utilized (current content: 1,744 sites with more than 42,000 soil animal records, RÖMBKE et al. 2012).

In order to facilitate the use of biocoenotical data at the landscape level, a standard frame of reference is needed. For the evaluation of observed changes within the scope of GMO-monitoring a site-specific reference system needs to be developed. It can be described as comprising reference values for the biocoenosis at certain habitat types by evaluating biocoenosis-site-relationships and will ultimately lead to the identification of threshold values with which a significant change of the biocoenosis can be indicated (see figure).

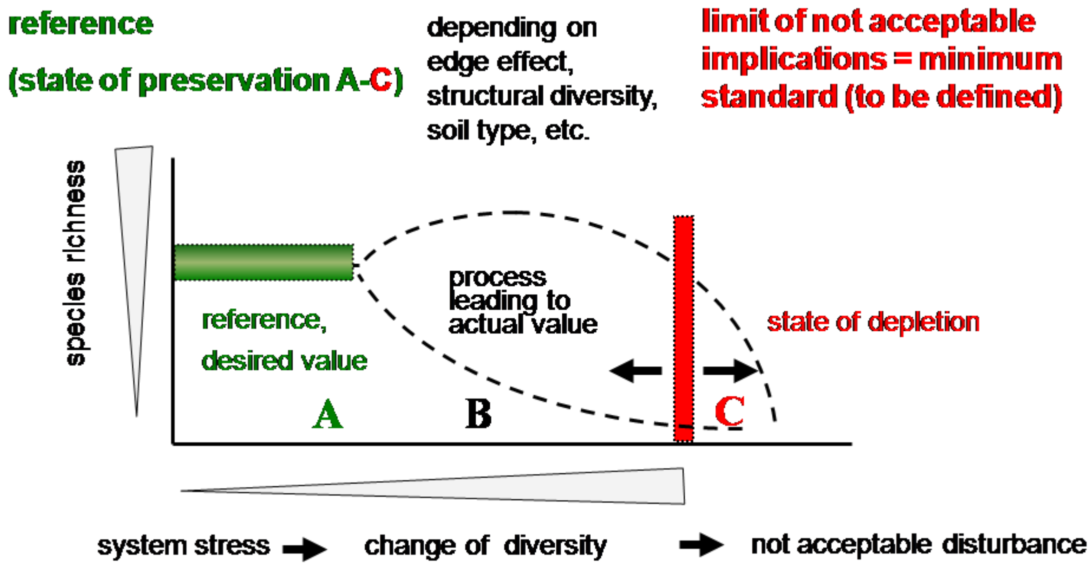

Derivation of threshold values regarding states of preservation (A, B, C; RÖMBKE et al. 2012) related to system stress (EU 1992).

Thus, a reference system for the site-specific diversity of soil organisms consists of:

- Reference values: lists of species expected to occur at a certain site with its specific conditions (e.g., climate, soil factors, region etc.);
- A quantification of deviations from these reference values that indicate impacted habitat function.

In order to develop reference values that link soil and site parameters with the occurrence of soil organisms, the landscape had to be classified into a limited number of “site categories”. The use of the habitat classification concept compiled in the German Red Data Book on endangered habitats (RIECKEN et al. 2009) ensures the compatibility with other monitoring approaches, nature conservation management, and prospectively also pesticide registration. When analyzing different organism groups, correlations between the occurrence of species and the corresponding hierarchical level within the system of biotope types became apparent (RÖMBKE et al. 2012). Further analysis demonstrated that the composition of communities depends on site properties. A comprehensive ecological assessment of sites requires the integration of different relevant organism groups on the species level thus at the same time covering their function (e.g., organic matter decomposition).

In summary, it can be concluded that this approach to assess the impact of anthropogenic stressors (including GMO) at selected arable sites is possible. The assessment is not based on a direct comparison of a certain GMO-field with a neighbouring BDF, but with reference values that are, among others, derived from other BDF sites. This approach is proposed for the assessment of soil quality, e.g. in the Netherlands (BISQ), where both structural and functional endpoints are utilized for various organism groups (RUTGERS et al. 2008).

Proposals for a future development of the monitoring

The establishment of a biodiversity monitoring of GMO requires generating a data basis representative for Germany, to enable the derivation of reference values for relevant biotope types. Based on our analysis it can be stated that the BDF could provide a suitable basic grid. The development of references on GMO uninfluenced sites is necessary for the assessment of the impact of GMO on the soil biocoenosis. If BDF are to be used for this purpose it would be necessary to protect them from exposure towards GMO.

The following recommendations are mostly valid for the monitoring and soil biodiversity assessment in Germany in general (see also RÖMBKE et al. 2012), and thus also for the permanent soil monitoring program and the monitoring of GMO. Recommendations for a minimum scheme for the GMO-monitoring mainly comprise five topics:

1. Recommendation of a minimum set of sites: representativeness (e.g., for biotope types, federal states, landscape, etc.) of a site grid and integration of additional site specifics like site history, e.g. regarding previous anthropogenic impacts
2. Recommendation of a minimum data set for site characterization: all measurements are to be performed according to ISO guidelines (or equivalent standards) (see also ISO 2011, TURBÉ et al. 2010, ENVIRONMENT CANADA 2012)
3. Recommendations of standard methods for a biological monitoring in general selection of organisms groups for a sufficient representation of the protection goal biodiversity and spatially and temporally representative sampling methods (VDI 2012)
4. Recommendations of standard methods for a biological monitoring on BDF for the purpose of GMO-monitoring
5. Recommended properties of a harmonized evaluation concept. Standards for data management and facilitation of a standardized evaluation

When it comes to data evaluation, the term of a “harmful soil alteration” first needs to be specified to be applicable in the context of GMO-monitoring. Until now there is no definition of habitat specific reference values for single organism groups or communities. These need to be developed using the above described monitoring concept. Any deviation from these reference values needs then to be evaluated according to previously determined threshold values. So far there is no regulation regarding effects on the soil biocoenosis in the German Federal Soil Protection Act or other laws, but only precautionary, trigger and action values for substances in the German Federal Soil Protection Ordinance (i.e., certain concentrations of single chemicals must not be exceeded in soils with a certain land use; BBODSCHV 1999).

Evaluation of data availability and possibilities of data analysis for the GMO-monitoring

German BDF sites and thus the data generated from same are subject to the sovereignty of the German federal states. The technical coordination is performed by a working group on soil protection (LABO, https://www.labo-deutschland.de/Kontakt.html), which was set up by representatives of German federal and state agencies. First the question, whether the installation of protection zones (no cultivation on or close to BDF) as demanded above is compatible with the core concept of the BDF program, needs to be answered on the federal state and national level. The observation of soil biology and the derivation of reference values for the evaluation of potential effects of GMO are aspects new to the BDF program.

The usability of data for a nationwide monitoring of GMO requires a standardized collection and management of data among all federal states to facilitate a central evaluation of the results (e.g., landscape- or culture-based) (PLACHTER et al. 2002; TOSCHKI 2008; ZÜGHART et al. 2008). In this context it has to be noted that:

1. The various federal state BDF programs currently differ in their structure regarding data collection, management and evaluation;
2. The data will become more valuable through long term, comparable measurements. Hence, continuity regarding data management should be established through a centralized coordination, e.g. at a long-existing institution such as a museum or a federal research institute;
3. Uniform and comparable data necessary for the evaluation within a GMO-monitoring need to be analyzed and discussed;
4. Minimum standards for data flow and management need to be provided.

Qualified and independent committees need to be nominated for performing nationwide data evaluation. The competent authority could resort to the already existing working group for the evaluation of BDF data, consisting of representatives from both federal and federal state authorities. The Federal Environmental Agency (UBA) is the contact for nationwide data evaluation (Fachgebiet II 2.7 "Bodenzustand, Bodenmonitoring"; Dr. Frank Glante, Wörlitzer Platz 1, D-06844 Dessau-Roßlau; Tel: 0340 2103 3434; e-mail: frank.glante@uba.de; http://www.umweltbundesamt.de/boden-und-altlasten/index.htm).

Within the data evaluation concept other options of use should also be considered, e.g. for the risk assessment of pesticides that pursues the same or similar protection goals. A sensible combination of prospective and retrospective risk assessment should be sought in order to appeal to future options according to their potential. The increasing use of computer-based models for risk analysis will likely play an essential role at this in the future (EFSA 2010, GALIC et al. 2010, GRIMM ET AL. 2009, PREUSS et al. 2009).

References

BBODSCHV (1999): Bundes-Bodenschutz- und Altlastenverordnung. Verordnung zur Durchführung des Bundes-Bodenschutzgesetzes. BGBl I, 36, S. 1554 – 1582 vom 16.06.1999.

BIOSICHERHEIT (2010): Coexistence of genetically modified and nongenetically modified maize: Making the point on scientific evidence and commercial experience. http://www.pgeconomics.co.uk/pdf/Co-existence_maize_10october2006.pdf, (28.05.2010).

CANDOLFI, M. P., BROWN, K., GRIMM, C., REBER, B., SCHMIDLI, H. (2004): A faunistic approach to assess potential side effects of genetically modified Bt-corn on nontarget athropods under field conditions. Biocontrol Sci. Techn. 14: 129-170.

CHAMBERLAIN, P.M., BULL, I.D., BLACK, H.J., INESON, P., EVERSHED, R.P. (2006): Collembolan trophic preferences determined using fatty acid distributions and compound-specific stable carbon isotope values. Soil Biol. Biochemistry 38: 1275-1281.

CORTET, J., ANDERSEN, M.N., CAUL, S. et. al (2006): Decomposition processes under Bt (*Bacillus thuringiensis*) maize: Results of a multi-site experiment. Soil Biology and Biochemistry 38: 195-199.

EC (EUROPEAN COMMISSION). (2001): EU Directive 2001/18/EC of 12 March 2001 on the deliberate release into the environment of genetically modified organisms and repealing Council Directive 90/220/EC. Official Journal of the European Communities L 106/1-38 (17.4.2001).

ENVIRONMENT CANADA (2012): Guidance Document on the Sampling and Preparation of Contaminated Soil for Use in Biological Testing. EPS 1/RM/53, 222 p.

EFSA (EUROPEAN FOOD SAFETY AUTHORITY) (2005): Guidance document of the scientific panel on genetically modified organisms for the risk assessment of genetically modified plants and derived food and feed. The EFSA Journal 99: 1-94.

EFSA (EUROPEAN FOOD SAFETY AUTHORITY (2010): Scientific Opinion on the development of specific protection goal options for environmental risk assessment of pesticides, in particular in relation to the revision of the Guidance Documents on Aquatic and Terrestrial Ecotoxicology (SANCO/3268/2001 and SANCO/10329/2002). EFSA Journal. (PPR). 8(10).

ESCHENBACH, C., WINDHORST, W. (2009): Indikatoren für die nationale Strategie zur biologischen Vielfalt: Gentechnik in der Landwirtschaft. Report for the Federal Agency for Nature conservation, Bonn, 141 pp.

EU (EUROPEAN UNION) (1992): Council Directive 92/43/EEC 1992 on the conservation of natural habitats and of wild fauna and flora.

GALIC, N., HOMMEN, U., BAVECO, J.M., VAN DEN BRINK, P.J. (2010): Potential application of population models in the European ecological risk assessment of chemicals II: Review of models and their potential to address environmental protection aims. Integrated Environmental Assessment and Management 6: 338-360.

GENTPFLEV (2008): Verordnung über die gute fachliche Praxis bei der Erzeugung gentechnisch veränderter Pflanzen. Gentechnik-Pflanzenerzeugungsverordnung, BGBl. I: 655.

GRIMM, V., ASHAUER, R., FORBES, V.E. et. al. (2009): CREAM: a European project on mechanistic effect models for ecological risk assessment of chemicals. Environ Sci Poll Res 16(6): 614-617.

HALSEY M.E., REMUND K.M., DAVIS C.A. et. al. (2005): Isolation of maize from pollen-mediated gene flow by time and distance. Crop Science Society of America 45: 2172-2185.

HILBECK, A., JÄNSCH, S., MEIER, M., RÖMBKE, J. (2008): Analysis and validation of present ecotoxicological test methods and strategies for the risk assessment of genetically modified plants. BfN-Skripten 236. 287 pp.

HOFMANN, F., JANICKE, U., JANICKE, L., WACHTER, R., KUHN, U. (2008a): Modellrechnungen zur Ausbreitung von Maispollen unter Worst-Case-Annahmen mit Vergleich von Freilandmeßdaten. Survey report. http://www.bfn.de/0301_veroe.html.

ISO (INTERNATIONAL ORGANIZATION FOR STANDARDIZATION) (2008a): Soil quality — Guidance for the selection and application of methods for the assessment of bioavailability of contaminants in soil and soil materials. ISO 17402. Geneva, Switzerland.

ISO (INTERNATIONAL ORGANIZATION FOR STANDARDIZATION) (2011): Soil quality – Guidance on the establishment and maintenance of monitoring programmes. ISO 16133. Geneva, Switzerland.

LUDY, C., LANG, A. (2006): A 3-year field-scale monitoring of foliage-dwelling spiders (Araneae) in transgenic Bt maize fields and adjacent field margins. Biocontrol 38: 314- 324.

MIDDELHOFF, U., HILDEBRANDT, J., BRECKLING, B. (2006): Die Ökologische Flächenstichprobe als Instrument eines GVO-Monitoring. BfN-Skripten 172. Ergebnisse des gleichnamigen F+E-Vorhabens im Auftrag des Bundesamtes für Naturschutz, BfN, Bonn.

PEIJNENBURG, W., JENSEN, J., KULA, C. et. al. (2012): Evaluation of exposure metrics for effect assessment of soil invertebrates. Critical Reviews in Environmental Science and Technology 42: 1862-1893.

PLACHTER, H., BERNOTAT, D., MÜSSNER, R. & RIECKEN, U. (2002): Entwicklung und Festlegung von Methodenstandards im Naturschutz. Schr.R. f. Landschaftspfl. u. Naturschutz 70: 566 S.

PREUSS, T.G., HOMMEN, U., ALIX, A. ET. AL. (2009): Mechanistic effect models for ecological risk assessment of chemicals (MEMoRisk)-a new SETAC-Europe Advisory Group. Environ Sci Poll Res 16(3): 250-252.

PRIESTLEY, A.L., BROWNBRIDGE, M. (2008): Field trials to evaluate effects of Bt-transgenic silage corn expressing the Cry1Ab insecticidal toxin on non-target soil arthropods in northern New England, USA. Transgenic Res.

RIECKEN, U., P. FINCK, U. RATHS, E. SCHRÖDER & A. SSYMANK (2009): German Red Data Book on endangered habitats (short version. July 2009). German Federal Agency for Nature Protection. Bonn-Bad Godesberg. Germany.

RÖMBKE, J., JÄNSCH, S., ROß-NICKOLL, M. et. al (2012): Erfassung und Analyse des Bodenzustands im Hinblick auf die Umsetzung und Weiterentwicklung der Nationalen Biodiversitätsstrategie. UBA-Texte 33/2012, 386 pp.

Roß-Nickoll, M., Lennartz, F., Fürste, A. et. al (2004): Die Arthropodenfauna von grasigen Feldrainen (off crop) und die Konsequenzen für die Bewertung der Auswirkungen von Pflanzenschutzmitteln auf den terrestrischen Bereich des Naturhaushaltes. UBA Texte Berlin.

Ruf A., Beylich, A., Blick, T. et. al (2013): Soil organisms as an essential element of a monitoring plan to identify the effects of GMO cultivation. Requirements – Methodology – Standardisation. BioRisk 8: 73–87. doi: 10.3897/biorisk.8.3255

Rutgers, M., Mulder, C., Schouten, A.J. et. al (2008): Soil ecosystem profiling in the Netherlands with ten references for biological soil quality. - RIVM-Report 607604009. 85 pp.

Squire, G.R., Brooks, D.R., Bohan, D.A. et. al. (2003): On the rationale and interpretation of the Farm Scale Evaluations of genetically modified herbicide-tolerant crops. Philosophical Transactions of the Royal Society B: Biological Sciences 358: 1779 –1799.

Theißen, B., Russell, D.J. (2009): Zur Bedeutung von Collembolen im GVO-Monitoring. Gefahrstoffe – Reinhaltung der Luft 69: 391-394.

Toschki, A., Hothorn, L.A., Roß-Nickoll, M. (2007): Effects of cultivation of genetically modified Bt maize on epigeic arthropods (Araneae; Carabidae). Environ. Entomol. 36: 966-980.

Toschki, A. (2008): Eignung unterschiedlicher Monitoring-Methoden als Grundlage zum Risk Assessment für Agrarsysteme am Beispiel einer biozönologischen Reihenuntersuchung und einer Einzelfallstudie. Dissertation RWTH Aachen, Institut für Umweltforschung.

Turbé, A., De Toni, A., Benito, P. et. al. (2010): Soil biodiversity: functions, threats, and tools for policy makers. BioIntelligence Service, IRD, and NIOO, Report for European Commission (DG Environment), Brussels, Belgium. 250 pp.

UBA (Umweltbundesamt) (2011): http://www.umweltbundesamt.de/boden-und-altlasten/boden/bodenschutz/dauerbeobachtung.htm. Letzte Änderung: 29.12.2011.

VDI (Verein Deutscher Inegnieure) (2012): Monitoring der Wirkungen von gentechnisch veränderten Organismen (GVO) – Wirkungen auf Bodenorganismen. VDI 4331 Blatt 1 (Entwurf), 48 S.

Züghart, W., Breckling, B. (2003): Konzeptionelle Entwicklung eines Monitoring von Umweltwirkungen transgener Kulturpflanzen Teil 1. – UBA Texte Berlin.

Züghart, W., Raps, A., Wust-Saucy, A.-G., Dolezel, M., Eckerstorfer, M. (2011): Monitoring of genetially modified organisms. A policy paper representing the view of the National Environment Agencies in Austria and Switzerland and the Federal Agency for Nature Conservation in Germany. Umweltbundesamt GmbH, Vienna, Austria, 56 p.
